# A visible-light-promoted radical reaction system for azidation and halogenation of tertiary aliphatic C–H bonds†
†Electronic supplementary information (ESI) available. See DOI: 10.1039/c5sc04169d


**DOI:** 10.1039/c5sc04169d

**Published:** 2016-01-11

**Authors:** Yaxin Wang, Guo-Xing Li, Guohui Yang, Gang He, Gong Chen

**Affiliations:** a State Key Laboratory and Institute of Elemento-Organic Chemistry , Collaborative Innovation Center of Chemical Science and Engineering (Tianjin) , Nankai University , Tianjin 300071 , China . Email: gongchen@nankai.edu.cn; b Department of Chemistry , The Pennsylvania State University , 104 Chemistry Building , University Park , PA 16802 , USA . Email: guc11@psu.edu

## Abstract

A highly tunable radical-mediated reaction system for the functionalization of tertiary aliphatic C–H bonds was developed. Reactions of various substrates with the Zhdankin azidoiodane reagent **1**, Ru(bpy)_3_Cl_2_, and visible light irradiation at room temperature gave C–H azidated or halogenated products in an easily controllable fashion. These reactions are efficient, selective, and compatible with complex substrates. They provide a potentially valuable tool for selectively labeling tertiary C–H bonds of organic and biomolecules with tags of varied chemical and biophysical properties for comparative functional studies.

## Abstract 2

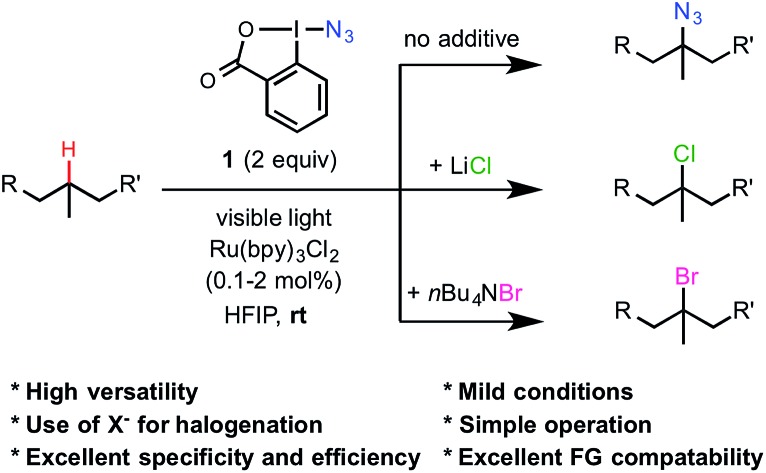
A highly tunable visible-light-promoted reaction system for the radical-mediated functionalization of tertiary aliphatic C–H bonds of complex substrates has been developed.

## Introduction

C–H bonds are the most prevalent chemical bonds on the surface of organic and biomolecules. Better means for the selective and controllable functionalization of C–H bonds could greatly facilitate a broad range of applications such as tagging organic and biomolecules, drug development and mapping ligand–receptor interactions.^1^,^2^ Despite their tremendous potential, the low reactivity of C–H bonds and the difficulty of achieving selectivity pose a significant challenge for the realization of a C–H labeling strategy, especially for more inert aliphatic C–H bonds. Radical reactions could provide a simple yet powerful approach to selectively target specific aliphatic C–H bonds due to their inherent ability to differentiate aliphatic C–H bonds based on the bond dissociation energy.^3^–^5^ While various radical aliphatic C–H functionalization reactions have been well-studied, new methods with better reactivity, versatility and biocompatibility are necessary for the selective and efficient C–H labeling of complex substrates.

**Scheme 1:**
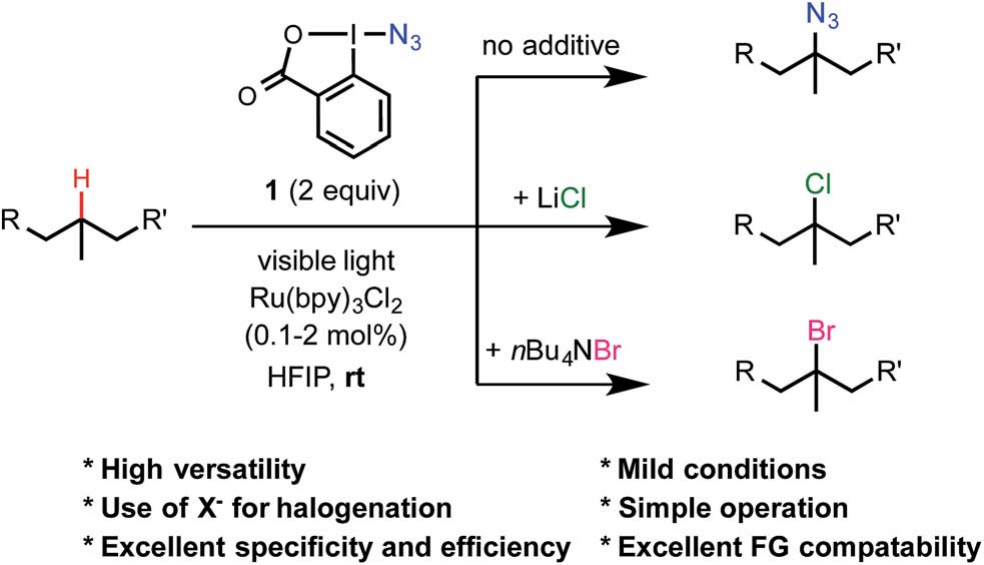
A highly tunable radical-mediated reaction system for the selective functionalization of 3° C(sp^3^)–H bonds under the promotion of visible light.

Among reported aliphatic C–H functionalization reactions, C–H azidation has proven to be particularly useful due to the unique photophysical and chemical reactivity of the azido group.^6^–^14^ In 1996, Zhdankin^8^ reported that the reaction of simple hydrocarbons with azidoiodane **1** in the presence of benzoyl peroxide led to the selective azidation of 3° and activated 2° C–H bonds in moderate to good yield.^9^–^11^ Hartwig recently discovered that Fe/PyBOX catalysts promote aliphatic C–H azidation with **1** under milder conditions, allowing the labeling of complex natural products with high selectivity.^12^,^13^ Herein, we report a new strategy to affect the azidation of 3° C–H bonds of complex substrates using the Zhdankin reagent **1**, a photosensitizer and visible-light irradiation at room temperature (Scheme 1). Furthermore, this visible-light-promoted radical reaction system can be conveniently modulated by the addition of nucleophilic halides to achieve aliphatic C–H chlorination and bromination with high efficiency and chemospecificity.

## Results and discussion

Our research in radical C–H functionalization chemistry stems from our interest in the synthetic and biological studies of peptides.^15^ Compared with α-amino acid (αAA) residues bearing polar or aromatic side chains, the structural and functional roles of hydrophobic aliphatic αAA residues such as leucine and valine in peptides and proteins are much more difficult to probe due to the lack of suitable chemical handles in these residues. Following the report of Hartwig's Fe-catalyzed aliphatic C–H azidation,^12^ we wondered whether this azidation chemistry could potentially provide a long sought-after method for selectively labeling the aliphatic αAA residues of peptides and proteins. As shown in Table 1, we commenced our investigation with the azidation of *N*-phthaloyl leucine methyl ester **2** with **1** under various reported conditions (Table 1). The reaction of **2** under Fe/iPr-PyBOX-catalyzed conditions in CH_3_CN provided the desired azidated product **3** in low yield (entry 1). The reaction under benzoyl peroxide-promoted conditions also gave **3** in moderate yield (entry 4). Interestingly, the azidation of Leu **2** proceeded in good yield without any catalyst or additive at 80 °C in hexafluoroisopropanol (HFIP) under an air atmosphere, forming *ortho*-iodobenzoic acid as the main byproduct (entry 9).

**Table 1:** 3° C–H azidation and chlorination of Leu **2**

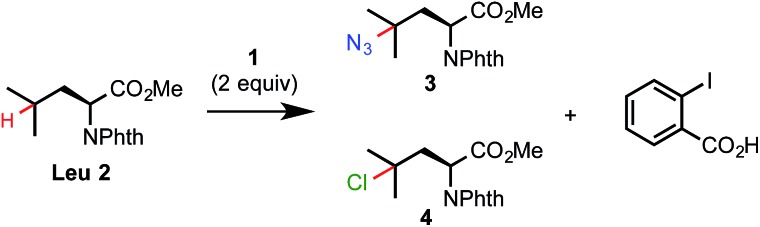						
Entry	Reagents (equiv.)/atmosphere	Solvent	*T* (°C)/time (h)	Yield^*a*^ (%)		
				3		4
1	Fe(OAc)_2_ (0.1), iPr-PyBOX (0.11), air	CH_3_CN	50/24	27	0	
2	Fe(OAc)_2_ (0.1), air	CH_3_CN	50/24	30	0	
3	Air	CH_3_CN	50/24	37	0	
4	Benzoyl peroxide (0.2), air	DCE	50/24	41	0	
5	Benzoyl peroxide (0.2), air	HFIP	rt/24	7	0	
6	Air	CH_3_CN	80/24	42	0	
7	Air	H_2_O	80/24	30	0	
8	Air	DCM/H_2_O (1 : 9)	80/24	51	0	
9	Air	HFIP	80/24	70	0	
10	Ir(ppy)_3_ (0.05), WL^*b*^, Ar	HFIP	rt/24	<2	0	
11	Ru(bpy)_3_Cl_2_ (0.05), VL, Ar	HFIP	rt/24	46	<5	
12	Ru(bpy)_3_Cl_2_ (0.001), VL, Ar	HFIP	rt/24	91 (86^*c*^)	<3	
13	Ru(bpy)_3_Cl_2_ (0.0001), VL, Ar	HFIP	rt/24	70	<3	
14	Ru(bpy)_3_Cl_2_ (0.2), VL, Ar	HFIP	rt/24	15	35	
15	Ru(bpy)_3_Cl_2_ (0.001), MgCl_2_ (2), VL, Ar	HFIP	rt/24	5	30	
16	Ru(bpy)_3_Cl_2_ (0.001), TMSCl (2), VL, Ar	HFIP	rt/24	<2	87	
17	Ru(bpy)Cl_2_ (0.001), LiCl (4), VL, Ar	HFIP	rt/24	<2	95 (77^*c*^)	
18	LiCl (4), VL, Ar	HFIP	rt/24	0	0	
19	Ru(bpy)_3_Cl_2_ (0.001), TEMPO (2), VL, Ar	HFIP	rt/24	<2	0	
20	Ru(bpy)_3_Cl_2_ (0.001), Ar, in darkness	HFIP	rt/24	<2	0	
21	VL, Ar (no Ru(bpy)_3_Cl_2_)	HFIP	rt/24	6	0	

^*a*^Yields are based on ^1^H-NMR analysis on a 0.2 mmol scale.

^*b*^VL: 12 W fluorescent bulb.

^*c*^Isolated yield (see ESI).

In order to achieve better biocompatibility, we next investigated whether the azidation reaction could proceed at room temperature. While the use of various electron transfer reagents such as zinc metal and cerium ammonium nitrate failed, we were delighted to find that the application of a photosensitizer (Ru(bpy)_3_Cl_2_) and visible light irradiation (VL: 12 W household fluorescent light bulb) formed **3** in moderate yield under an Ar atmosphere (entry 11).^16^ Interestingly, the use of 0.1 mol% of Ru(bpy)_3_Cl_2_ gave significantly improved results than when using 5 mol% of Ru(bpy)_3_Cl_2_ (entry 12). Furthermore, we were surprised to find that the use of 20 mol% of Ru(bpy)_3_Cl_2_ produced only a small amount of **3** but instead produced a 35% yield of chlorinated product **4** (entry 14). This result prompted us to evaluate different Cl donors to improve the yield of the chlorinated product (see ESI† for more screening results). A number of nucleophilic Cl sources worked, LiCl or TMSCl gave **4** in the highest yield and selectivity (entries 16 and 17). The addition of 2 equiv. of TEMPO shut down the reaction with little conversion of **2** (entry 19). The reaction in darkness gave little product (entry 20). Interestingly, visible light irradiation in the absence of Ru(bpy)_3_Cl_2_ also gave a small amount of **3** (entry 21).

**Scheme 2:**
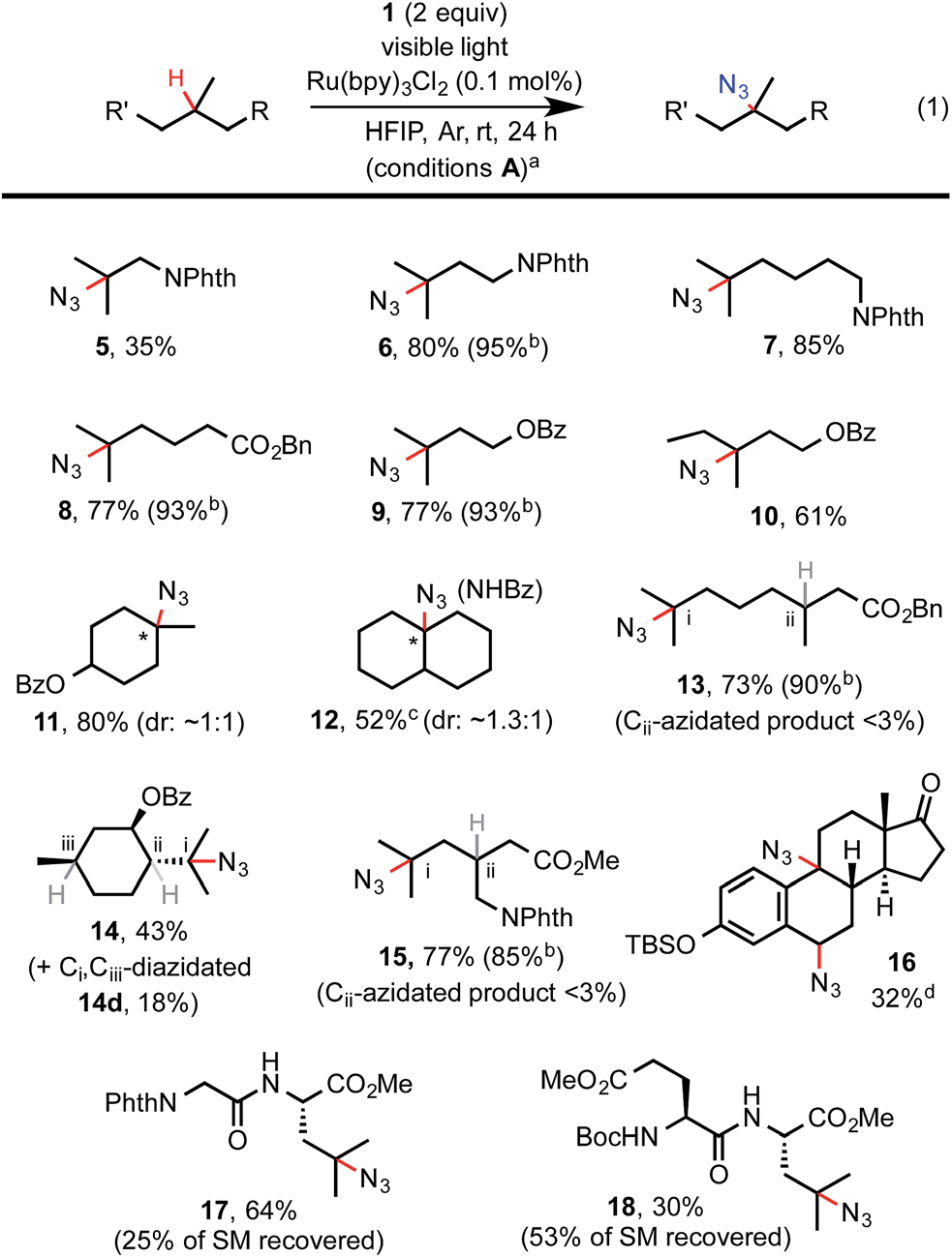
Substrate scope of aliphatic C–H azidation. [a] Isolated yield on a 0.2 mmol scale. [b] ^1^H-NMR yield. Isolated yield was occasionally compromised by the volatility of the product and/or difficulties in purification. [c] Isolated yield of its amide derivative (see ESI†). [d] As the only major product.

As shown in Scheme 2, C–H azidation reactions with azidoiodane **1** worked well for a variety of aliphatic substrates with excellent 3° C–H selectivity under the optimized light-promoted conditions **A**.^17^ Common functional groups *e.g.* Boc, Bn, TBS, ester and amide were well tolerated. Steric hindrance and electron-withdrawing functional groups generally diminish the reactivity of the neighbouring C–H bonds, allowing preferential labeling of certain 3° C–H bonds of complex substrates.1b,e For instance, azidation selectively took place at the more distal 3° carbon of **13** and *N*-phthaloyl pregabalin methyl ester **15**. Moreover, we were delighted to find that the C–H azidation of dipeptides under the standard light-promoted conditions also provided good yield and excellent selectivity (see **17** and **18**).

**Scheme 3:**
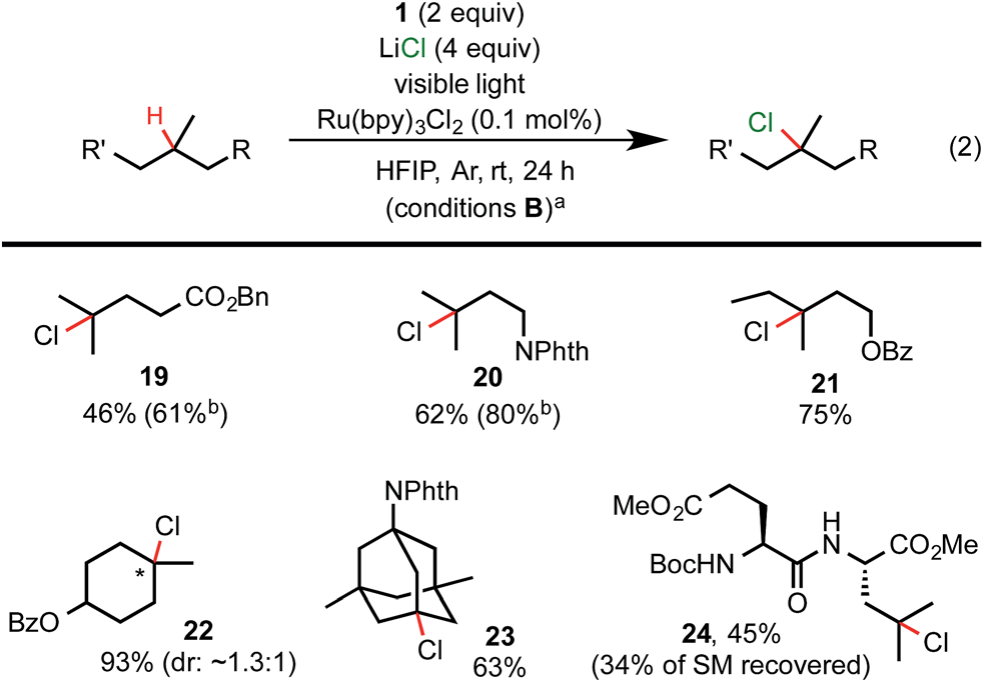
Substrate scope of C–H chlorination. (a) Isolated yield on a 0.2 mmol scale and (b) ^1^H-NMR yield.

**Scheme 4:**
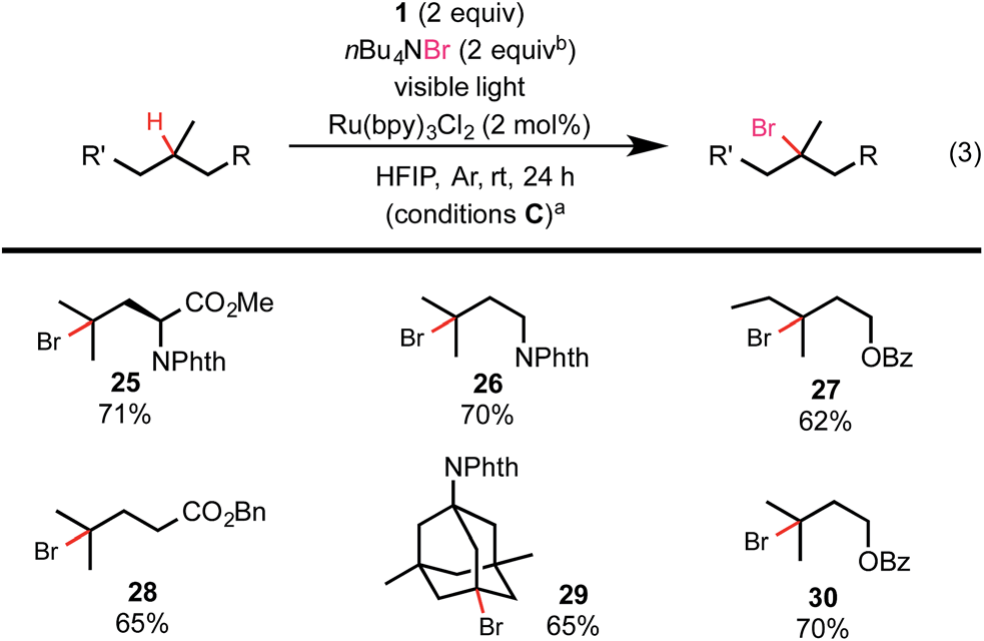
Substrate scope of C–H bromination. (a) Isolated yield on a 0.2 mmol scale; (b) added in 2 portions (1 + 1 equiv.).

As shown in Scheme 3, visible light-promoted 3° C–H chlorination reactions with azidoiodane **1** and LiCl as the Cl donor also demonstrated excellent efficiency, site-selectivity and functional group tolerance.^18^ The reactivity of these C–H chlorination reactions was slightly higher than the corresponding azidation reaction (see **21***vs.***10** and **24***vs.***18**). Additionally, we were pleased to find that this reaction system can be further modulated to form 3° C–H brominated products by the addition of a bromide donor.^19^,^20^ As shown in Scheme 4, the reaction of various aliphatic substrates with 2 equiv. of *n*Bu_4_NBr, 2 equiv. of **1**, and 2 mol% of Ru(bpy)_3_Cl_2_ under visible light irradiation (conditions C) gave 3° C–H brominated products in good yield and with excellent selectivity. It is worth noting that the competing C–H azidation process was completely suppressed in both chlorination and bromination reaction systems.

**Scheme 5:**
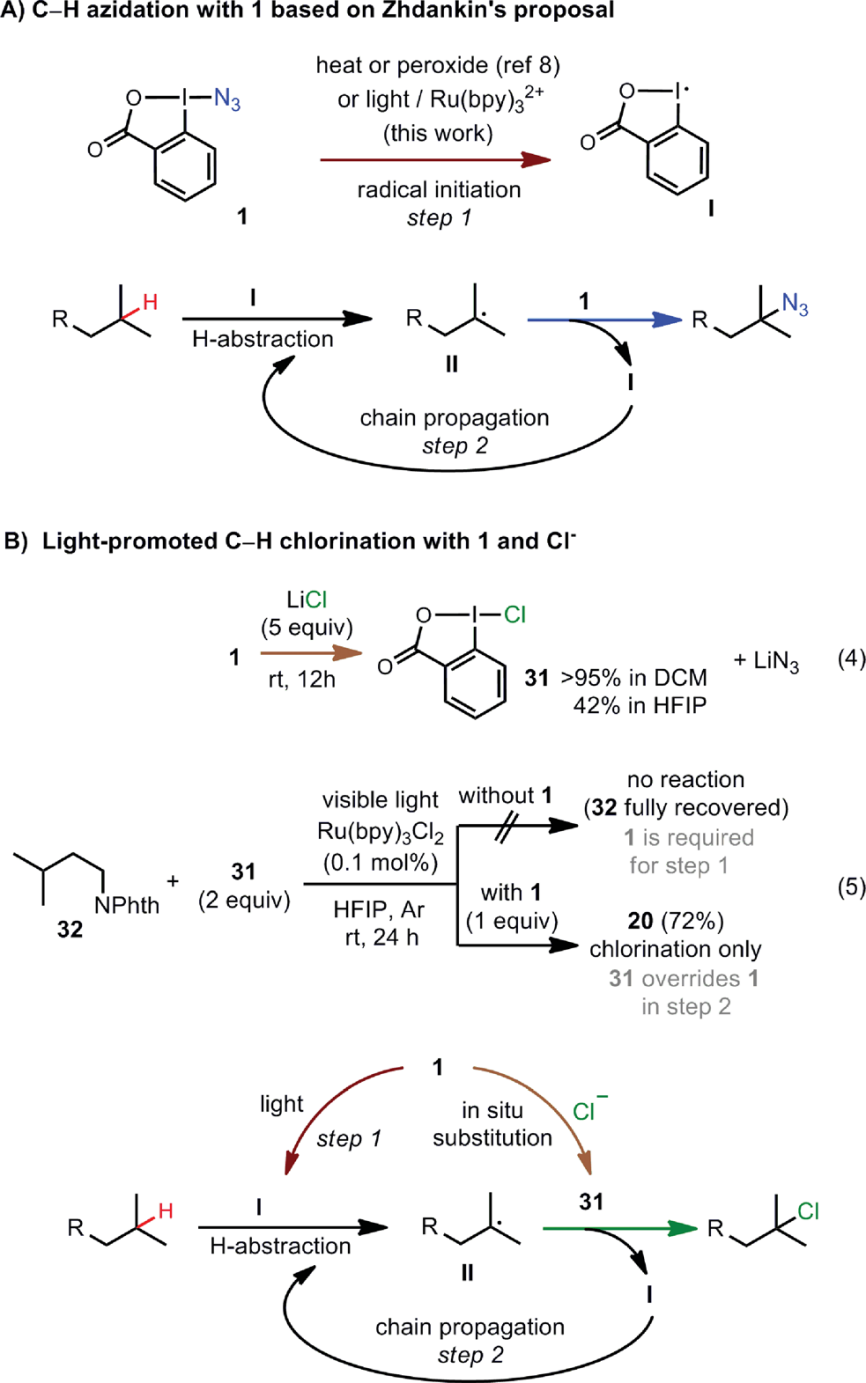
Plausible mechanisms for C–H azidation and chlorination.

The azidation reactions under the newly developed conditions are consistent with the radical chain mechanisms proposed by Zhdankin^8^ (Scheme 5A). The radical chain mechanism is supported by the large quantum yield (*Φ* ∼ 18, measured by Yoon's method^21^) of the C–H azidation reaction of **32** with **1** (see ESI†). Under thermally promoted conditions (see entry 9, Table 1), the weak I–N_3_ bond of **1** presumably undergoes homolytic cleavage to form an iodanyl radical **I** and an azido radical.^8^ Because of the relative weak reactivity the of N_3_ radical,^22^**I** likely serves as the H abstractor^23^ to convert the C–H substrate to a C-centered radical intermediate **II**. **II** then attacks **1** to form the azidated product and regenerates **I**, which propagates the radical chain reaction. Under visible-light irradiation in the presence of Ru(bpy)_3_Cl_2_, **1** may be activated by electron transfer^24^ to generate radical **I**, which can trigger the same chain reaction at rt. The chlorination reactions likely share the same mechanism for the azidoiodane-mediated radical initiation step (formation of **I**) as the azidation reactions. However, chloroiodane **31** 25 formed *via in situ* substitution of **1** with Cl^–^ probably overrides azidoiodane in the subsequent chain propagation step to selectively form the C–H chlorinated products (Scheme 5B). Our control experiments indicated that both **1** and **31** are involved in the chlorination reaction. As shown in eqn 4, mixing of **1** and LiCl in HFIP at rt in the absence of light can form **31** (42% yield in 12 h). Subjecting substrate **32** and 2 equiv. of pre-made **31** to the standard light-promoted conditions did not give any reaction (eqn 5). Interestingly, the addition of 1 equiv. of **1** together with **31** cleanly restored the chlorination reaction (see more details in ESI†).^26^ This suggests that chloride **31** cannot be directly activated to generate the iodanyl radical **I** for the initial H-abstraction, but is more reactive than azidoiodane toward nucleophilic attack by the radical intermediate **II**. C–H bromination with *n*Bu_4_NBr may follow a similar pathway as chlorination.

## Conclusions

In summary, we have developed a uniquely tunable radical-mediated reaction system for the azidation and halogenation of tertiary aliphatic C–H bonds of various substrates with azidoiodane **1** and visible light irradiation at room temperature. These reactions provide a simple and powerful method for selectively labeling tertiary aliphatic C–H bonds of organic molecules with diverse tags. In addition to a mild protocol for radical aliphatic C–H functionalization, this study demonstrated a novel strategy to use more easily available nucleophilic halide reagents for radical C–H halogenation reactions. Further mechanistic studies and expansion of this reaction system to achieve other types of aliphatic C–H functionalization and applications in labeling biomolecules are currently under investigation.

## Supplementary Material

Supplementary information

## References

[cit1] Godula K., Sames D. (2006). Science.

[cit2] Sletten E. M., Bertozzi C. R. (2009). Angew. Chem., Int. Ed..

[cit3] Chen M. S., White M. C. (2007). Science.

[cit4] Xia J.-B., Zhu C., Chen C. (2013). J. Am. Chem. Soc..

[cit5] Zhang K., Shafer B. M., Demars M. D., Stern H. A., Fasan R. (2012). J. Am. Chem. Soc..

[cit6] Brase S., Gil C., Knepper K., Zimmermann V. (2005). Angew. Chem., Int. Ed..

[cit7] Kita Y., Tohma H., Inagaki M., Hatanaka K., Yakura T. (1991). Tetrahedron Lett..

[cit8] Zhdankin V. V., Krasutsky A. P., Kuehl C. J., Simonsen A. J., Woodward J. K., Mismash B., Bolz J. T. (1996). J. Am. Chem. Soc..

[cit9] Magnus P., Lacour J., Weber W. (1993). J. Am. Chem. Soc..

[cit10] Ochiai M., Miyamoto K., Kaneaki T., Hayashi S., Nakanishi W. (2011). Science.

[cit11] Zhdankin V. V., Stang P. (2002). Chem. Rev..

[cit12] Sharma A., Hartwig J. F. (2015). Nature.

[cit13] For a Mn-based catalytic system that uses NaN_3_ as an azido source and PhIO as an oxidant to achieve the azidation of 3° and unactivated 2° C–H bonds of drug molecules at rt: Huang X. Bergsten T. M. Groves J. T., J. Am. Chem. Soc., 2015, 137 , 5300 . 25871027 10.1021/jacs.5b01983

[cit14] Zhang X., Yang H., Tang P. (2015). Org. Lett..

[cit15] Feng Y., Chen G. (2010). Angew. Chem., Int. Ed..

[cit16] Narayanam J. M. R., Stephenson C. R. J. (2011). Chem. Soc. Rev..

[cit17] Our light-promoted C–H azidation reactions showed similar selectivity and reactivity to Hartwig’s Fe-catalyzed reaction system (ref. 12)

[cit18] Liu W., Groves J. T. (2010). J. Am. Chem. Soc..

[cit19] Intermolecular selective aliphatic C–H bromination reactions using a complex substrate as the limiting reagent are still limited. These 3° C–H bromination reactions provide a useful complement to Alexanian’s visible-light promoted 2° C–H selective bromination using *N*-bromoamide reagents: Schmidt V. A. Quinn R. K. Brusoe A. T. Alexanian E. J., J. Am. Chem. Soc., 2014, 136 , 14389 . 25232995 10.1021/ja508469u

[cit20] Similar iodination and fluorination reactions with nucleophilic iodide and fluoride additives have been unsuccessful by far. See ref. 4 for the recent success of light-facilitated aliphatic C–H fluorination. For the acetoxyiodane-mediated 2° C–H iodination of hydrocarbons (used as co-solvent): Barluenga J. Camos-Gomez E. Rodriguez D. Gonzalez-Bobes F. Gonzalez J. M., Angew. Chem., Int. Ed., 2005, 44 , 5851 . 10.1002/anie.200501195 16092138

[cit21] For an excellent paper characterizing chain processes in visible light photoredox catalysis based on the measurements of quantum yield *Φ*: Crismesia M. A. Yoon T. P., Chem. Sci., 2015, 6 , 5426 , . A reaction with *Φ* ≫ 1 could only be consistent with a product-forming chain mechanism. Our “light/dark” experiment (see ESI) showed that the same C–H azidation reaction requires constant irradiation for product formation, which suggests a relatively short lifetime of the radical chain process .26668708

[cit22] Alfassi Z. B., Shuler R. H. (1985). J. Phys. Chem..

[cit23] Ochiai M., Ito T., Takahashi H., Nakanishi A., Toyonari M., Sueda T., Goto S., Shiro M. (1996). J. Am. Chem. Soc..

[cit24] For the related light-induced activation of hydroxybenziodoxole to form the iodanyl radical **I**, see: Huang H. Zhang G. Gong L. Zhang S. Chen Y., J. Am. Chem. Soc., 2014, 136 , 2280 . 24490981 10.1021/ja413208y

[cit25] The synthesis of chloroiodane **31** was recently reported by Togni as an intermediate in the preparation of the corresponding trifluoromethyl aryliodonium compound. Matoušek V. Pietrasiak E. Schwenk R. Togni A., J. Org. Chem., 2013, 78 , 6763 , . Its use for C–H functionalization reactions was unknown . 23734560 10.1021/jo400774u

[cit26] Reaction of **32** with 2 equiv. of **31** and 0.2 equiv. of benzoyl peroxide in HFIP at either rt or 80 °C gave no chlorinated product **20**

