# Supplementary material for: A visible-light-promoted radical reaction system for azidation and halogenation of tertiary aliphatic C–H bonds
Source: Chem Sci. 2016 Jan 11;7(4):2679–83. doi: 10.1039/c5sc04169d (PMC5477033; doi:10.1039/c5sc04169d)
Supplement: Supplementary file 1 [file SC-007-C5SC04169D-s001.pdf]

Electronic Supplementary Material (ESI) for Chemical Science.
This journal is © The Royal Society of Chemistry 2016

# Supporting Information

# A Visible-Light-Promoted Radical Reaction System for Azidation and Halogenation of Tertiary Aliphatic C−H Bonds

Yaxin Wang[1], Guoxing Li[1], Guohui Yang[1], Gang He[1] and Gong Chen[1,2]*

[1]State Key Laboratory and Institute of Elemento-Organic Chemistry, Collaborative Innovation Center of Chemical Science and Engineering (Tianjin), Nankai University, Tianjin 30071, China

[2]Department of Chemistry, The Pennsylvania State University, University Park, Pennsylvania 16802, United States

**Content**

1. **Reagents, S2**
2. **Instruments, S2**
3. **Synthesis of substrates, S3**
4. **Reaction optimization for C-H azidation and chlorination of Leu 2, S8**
5. **General procedures and substrate scope for C-H azidation, S10**
6. **General procedures and substrate scope for C-H chlorination, S16**
7. **General procedures and substrate scope for C-H bromination, S19**
8. **Chlorination of 32 with chloroiodane 31, S21**
9. **Quantum yield of C-H azidation of 32, S23**
10. **Light/dark experiments of C-H azidation of 32, S27**
11. **References, S28**
12. **$^{1}$H-NMR and $^{13}$C-NMR spectra, S29**

## 1. Reagents

All commercial materials were used as received unless otherwise noted. DCM and DMF were dried by distillation over $CaH_2$. THF and toluene were dried by distillation over sodium/benzophenone. TLC were performed on silica gel Huanghai HSGF254 plates and visualization of the developed chromatogram was performed by fluorescence quenching ($\lambda_{max}$ = 254 nm). Flash chromatography was performed using Silica gel (200-300 mesh) purchased from Qingdao Haiyang Chemical Co., China. Azidoiodane **1** [1-3] and chloroiodane **40** [4] was synthesized according to reported procedures and used as freshly prepared. Starting materials were synthesized according to reported procedures. LiCl (≥99%, Aladdin), $Bu_4NBr$ (99%, Energy Chemical), $Ru(bpy)_3Cl_2$ (98%, Ru＞15.75%, Energy Chemical) and HFIP (99.0%, J&K Chemical) were used in the azidation and halogenation reactions.

## 2. Instruments

NMR spectra were recorded on Bruker AVANCE AV 400 instruments and all NMR experiments were reported in units, parts per million (ppm), using residual solvent peaks as internal reference. Multiplicities are recorded as: s = singlet, d = doublet, t = triplet, dd = doublet of doublets, td = triplet of doublets, br s = broad singlet, m = multiplet. High resolution ESI mass experiments were operated on a Waters LCT Premier instrument. IR spectra were recorded on a Bruker Tensor 27 instrument and are reported in wavenumbers ($cm^{-1}$). All reactions were carried out in a 4 mL glass vial (Thermo SCIENTIFIC National B7999-2, made from superior quality 33 expansion borosilicate clear glass), sealed with PTEF cap on bench top.

**Note：** All azidation reactions and subsequent workup were performed behind a blast shield with the sash positioned as low as possible. Once isolated, organic azides were stored in a freezer and away from sources of heat, light, pressure and shock. While we did not encounter any issues during their synthesis, proper precautions were taken.

## 3. Synthesis of substrates

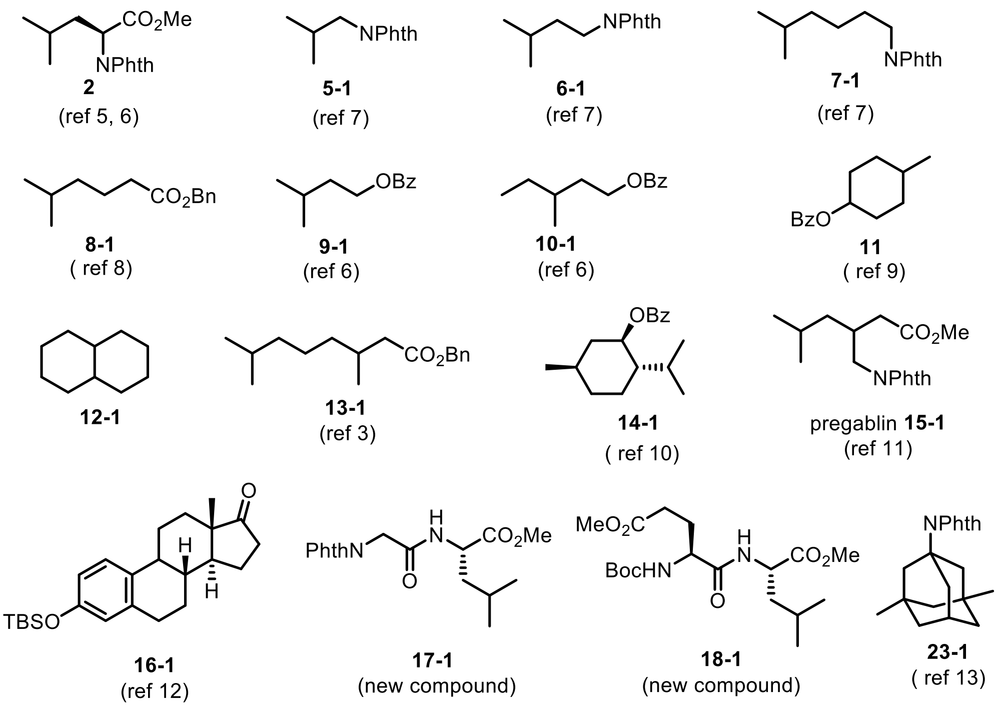


**Scheme S1**. List of all substrates used in this study

### 3.1 General procedure I for synthesis of protected amino acid substrates

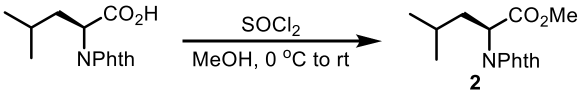


**Scheme S2**

To a solution of *N*-phthaloyl-*L*-leucine (1.0 g, 3.8 mmol, 1.0 equiv) in MeOH (10.0 mL) was added thionyl chloride (2.0 mL, 27.7 mmol, 7.2 equiv) at 0 °C. The reaction mixture was stirred at room temperature for 10 h before quenched with $H_2O$ (5.0 mL) and the mixture was extracted with $CH_2Cl_2$ (3 x 20.0 mL). The combined organic layer was washed with brine, dried over anhydrous $Na_2SO_4$ and concentrated *in vacuo*. The residue was purified by chromatography on silica gel (eluted with hexane/acetone (v/v 60:1)) to afford 0.9 g of compound **2** as a white solid (83% yield), spectra data are consistent with those reported in the literature.[5, 6]

### 3.2 General procedure II for synthesis of Phthal-protected amine substrates

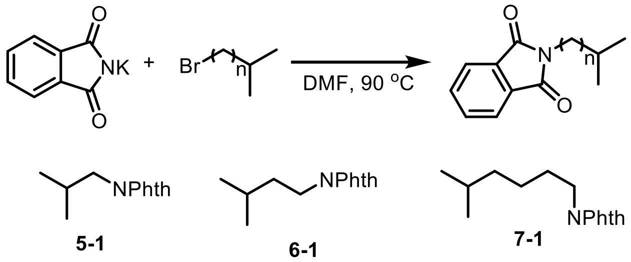


(known compounds, ref 7)

**Scheme S3**

Potassium phthalimide (21 mmol) was added to a solution of alkyl bromide (19.6 mmol) in 23 mL of anhydrous DMF. The mixture was heated to 90 °C for 16 h. After cooled to room temperature, the reaction mixture was poured into water (75 mL) and extracted with $CH_2Cl_2$ (3 x 50 mL). The combined organic phase was washed with 100 mL 0.2 M KOH (aq.) and water. The organic phase was dried over anhydrous $Na_2SO_4$, filtered and concentrated *in vacuo*. The resulting crude yellow oil was purified by silica gel column chromatography (eluted with hexane/acetone (v/v 40:1)) to afford the products **5-1, 6-1, 7-1,** spectra data are consistent with those reported in the literature.[7]

### 3.3 General procedure III for synthesis of benzyl esters

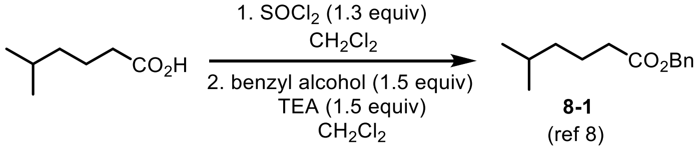


**8-1**
(ref 8)

**Scheme S4**

To a mixture of alkyl carboxylic acids (25 mmol, 1.0 equiv) in dry $CH_2Cl_2$ (50 mL) was added $SOCl_2$ (32 mmol, 1.3 equiv) at rt. The mixture was heated to 55 °C for 7 h under $N_2$. $CH_2Cl_2$ and excess of $SOCl_2$ were then removed under reduced pressure. The crude acyl chloride was dissolved in $CH_2Cl_2$ (50 mL), benzyl alcohol (20 mmol, 0.8 equiv) and TEA (37 mmol, 1.5 equiv) were added at 0 °C. The mixture was stirred at rt for 5 hours and then quenched with water, extracted with $CH_2Cl_2$ (3 x 20.0 mL). The organic layer was washed with water and brine, dried over anhydrous $Na_2SO_4$ and concentrated *in vacuo*. The residue

was purified by silica gel column chromatography (eluted with hexane/acetone (v/v 100:1)) to afford the products **8-1**, spectra data are consistent with those reported in the literature.[8]

**3.4 General procedure IV for synthesis of benzoates:**

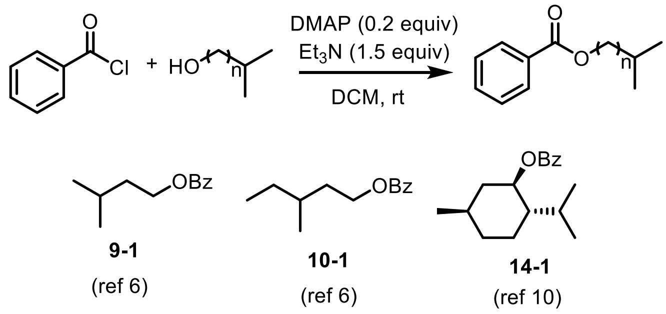


**Scheme S5**

To a solution of corresponding alcohol (10.0 mmol, 1.0 equiv), DMAP (24.4 mg, 2.0 mmol, 0.2 equiv) and $Et_3N$ (2.0 mL, 15.0 mmol, 1.5 equiv) in $CH_2Cl_2$ (50.0 mL) at 0 $^{o}C$ was added benzoyl chloride (1.68 g, 12.0 mmol, 1.2 equiv). The reaction mixture was stirred at rt for 6 h before quenched with $H_2O$ (10.0 mL) and the mixture was extracted with $CH_2Cl_2$ (20.0 x 3mL). The combined organic layer was dried over anhydrous $MgSO_4$, filtered and concentrated *in vacuo*. The residue was purified by silica gel chromatography (eluted with hexane/EtOAc (v/v 50:1)) to give alkyl benzoate **9-1, 10-1, 14-1** as colorless liquid, spectra data are consistent with those reported in the literature.[6, 10]

**3.5 Synthesis of 11-1**

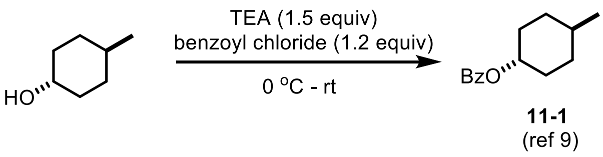


**Scheme S6**

To a solution of *trans*-4-methylcyclohexanol (228 mg, 2.0mmol, 1.0 equiv) and TEA (303 mg, 3.0 mmol, 1.5 equiv) in $CH_2Cl_2$ (10 mL) was added benzoyl chloride (281 mg, 2.4 mmol, 1.2 equiv) at 0 $^{o}C$. The reaction mixture was warmed to rt and stirred overnight. The solvent was removed *in vacuo* and the resulting residue was purified by silica gel flash chromatography to give the desired product **11-1** (253 mg, 58%), spectra data are consistent with those reported in the literature.[9]

### 3.6 Synthesis of compound 13-1

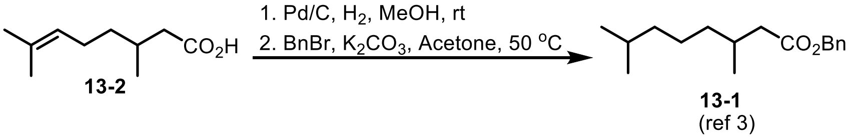


**Scheme S7**

To a solution of citronellyl acid (1.7 g, 10.0 mmol, 1.0 equiv) in 20 mL of MeOH was added Pd/C (0.3 g, 5% on carbon, wetted with ca. 55% water). The reaction mixture was stirred under a $H_2$ atmosphere (balloon) for 24 h at rt. The reaction mixture was filtered through a pad of celite and the solvent was removed under reduced pressure. The resulting residue was dissolved in 15 mL of acetone, then benzyl bromide (3.4 g, 20 mmol, 2.0 equiv) and $K_2CO_3$ (4.2 g, 3.0 equiv, 30 mmol) were added at rt. The resulting reaction mixture was heated to 50 $^{o}$C for 12 h. The solvent was removed under reduced pressure and the resulting residue was purified by silica gel column chromatography (eluted with petroleum hexanes/EtOAc 100:1 (v/v)) to give compound **13-1** as a colorless liquid (80% yield), spectra data are consistent with those reported in the literature.[3]

### 3.7 Synthesis of pregabalin 15-1

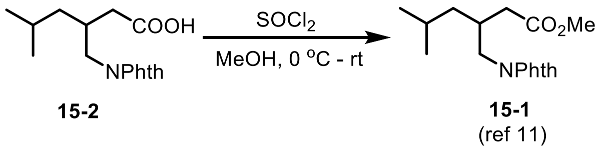


**Scheme S8**

*Pregablin* **15-1** was prepared following the same procedure as compound **2** in 91% yield, spectra data are consistent with those reported in the literature. [11]

### 3.8 Synthesis of 16-1

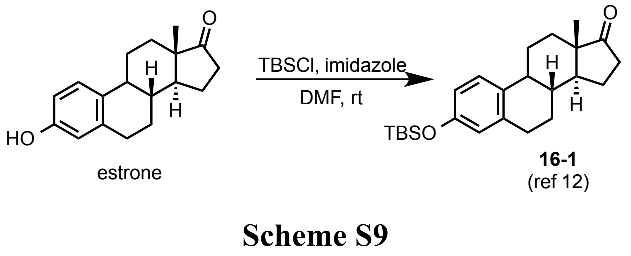


**Scheme S9**

To a stirred solution of estrone (1 g, 3.7 mmol) in anhydrous DMF (10 mL) was added imidazole (756 mg, 11.1 mmol) at room temperature. The mixture was cooled to 0 °C and a solution of tert-butyldimethylsilyl chloride (840 mg, 5.6 mmol) in 5 mL anhydrous DMF was added. The mixture was allowed to warm up to room temperature and stirred for 6 h. The mixture was diluted with $H_2O$ (75 mL) and the aqueous solution was extracted with Ethyl Acetate (3x 100 mL). The combined organic layer was washed with brine (15 mL) and dried over $Na_2SO_4$. The solvent removed under reduced pressure, and the resulting residue was purified by silica gel chromatography to the desired product. Spectra data are consistent with those reported in the literature. [12]

### 3.9 Synthesis of dipeptides 17-1 and 18-1

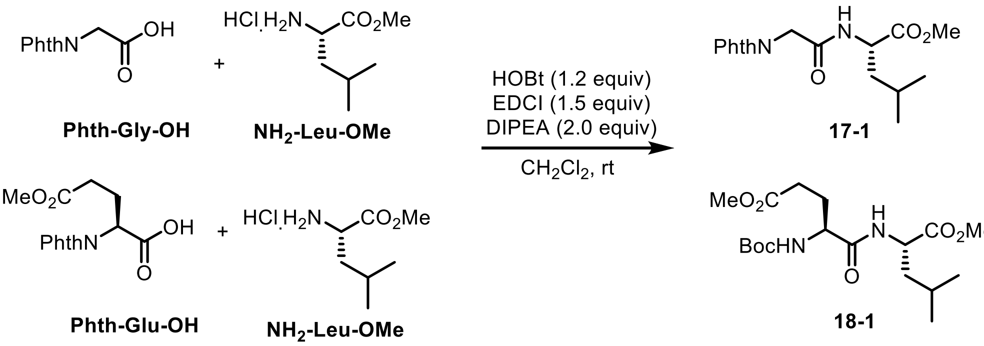


**Scheme S10**

General procedure for the amide coupling: A mixture of the corresponding N-Phthal-protected amino acid (5 mmol, 1.0 equiv), amino acid methyl ester hydrochloride (6 mmol, 1.2 equiv), HOBt (6 mmol, 1.2 equiv), EDCI (7.5 mmol, 1.5 equiv) and DIPEA (10 mmol, 2.0 equiv) in 25 mL of anhydrous $CH_2Cl_2$ were stirred at rt for 5 h. The reaction was quenched with 10 mL of water and the organic phase was washed with 10 mL of 1 M HCl (aq.) and brine. The solution was dried over anhydrous $Na_2SO_4$, and concentrated *in vacuo*. The resulting residue was purified by silica gel flash chromatography to give the desired products.

**Gly-Leu 17-1: $^1$H NMR** ($CDCl_3$, 400 MHz) δ 7.89-7.87 (m, 2H), 7.75-7.72 (m, 2H), 6.23 (br s, 1H), 4.66 (td, $J$ = 5.2, 8.4 Hz, 1H), 4.39 (d, $J$ = 10.0 Hz, 2H), 3.73 (s, 3H), 1.65-1.50 (m,

4H), 0.93 (d, $J$ = 1.6 Hz, 3H), 0.92 (d, $J$ = 1.6 Hz, 3H); **$^{13}$C NMR** ($CDCl_3$, 100 MHz) δ 173.23, 167.68, 165.77, 134.18, 131.94, 123.58, 52.41, 50.91, 41.78, 40.53, 24.72, 22.67, 21.94. **HRMS** Calcd for $C_{17}H_{20}N_2NaO_5$ [M+H$^+$]: 355.1264; Found:355.1270.

**Glu-Leu 18-1: $^1$H NMR** ($CDCl_3$, 400 MHz) δ 6.70 (d, $J$ = 7.2 Hz, 1H), 5.27 (d, $J$ = 7.6 Hz, 1H), 4.57 (td, $J$ = 4.4, 8.4 Hz, 1H), 4.21-4.15 (m, 1H), 3.71 (s, 3H), 3.68 (s, 3H), 2.55-2.41 (m, 2H), 2.16-2.07 (m, 1H), 1.96-1.89 (m, 1H), 1.68-1.50 (m, 3H), 1.42 (s, 9H), 0.92 (d, $J$ = 1.6 Hz, 3H), 0.91 (d, $J$ = 1.6 Hz, 3H); **$^{13}$C NMR** ($CDCl_3$, 100 MHz) δ 173.68, 172.94, 171.37, 155.50, 79.73, 53.17, 52.06, 51.62, 50.59, 40.89, 29.92, 28.10, 27.78, 24.56, 22.63, 21.56. **HRMS** Calcd for $C_{18}H_{32}N_2NaO_7$ [M+H$^+$]: 411.2102; Found: 411.2107.

### 3.10 Synthesis of 23-1

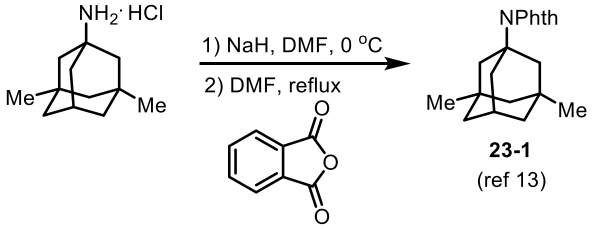


**Scheme S11**

Memantine hydrochloride (1.40 g, 5.6 mmol, 1.0 equiv) was dissolved in anhydrous DMF (15 mL) and cooled to 0 °C. Sodium hydride (134.4 mg, 5.6 mmol, 1.0 equiv) was added portion-wise. The reaction mixture was allowed to stirred at 0 °C for 10 min before warmed to rt over 30 min. Phthalic anhydride (1.24 g, 8.37 mmol, 1.5 equiv) was then added and the reaction mixture was heated at reflux overnight. The reaction mixture was then cooled to room temperature, diluted with $Et_2O$ and 1M HCl (aq.). The organic layer was separated and washed 1M HCl (aq.) (2 x 5 mL) and brine. The organic layer was dried over anhydrous $MgSO_4$ and concentrated *in vacuo*. The crude material was purified by silica gel column chromatography (10% EtOAc/Hexanes) to give *N*-Phth memantine **23-1** (1.25 g, 70% yield) as a white solid, spectra data are consistent with those reported in the literature.[13]

## 4. Reaction optimization for C-H azidation and chlorination of Leu 2

All screening reactions were carried out at a 0.2 mmol scale in a 4 mL glass vial (Thermo Scientific, National B7999-2). The vials were purged with Ar for 1 min if necessary, sealed with PTEF cap and stirred on bench top. Stock solution of $Ru(bpy)_3Cl_2$ (0.01 mmol in 5 mL of HFIP) was used if necessary. A 12 W household white fluorescent bulb was positioned 10 cm aside from the reaction vials if necessary.

Leu **2** (55 mg, 0.2 mmol, 1.0 equiv) and other specified reagents were first dispersed in 0.1 mL of solvent and stirred for 5 min at rt. Reagent **1** (115.6 mg, 0.4 mmol, 2.0 equiv) was then added and the resulting mixture was vigorously stirred at specified temperature with or without light irradiation for 24 h. After removal of the solvent *in vacuo*, the resulting residue was dissolved in 1 mL of $CD_3OD$ along with $Cl_2CHCHCl_2$ (20 μL) as an internal standard for $^1$H-NMR analysis. The composition of reaction mixture was analyzed based on the methyl peaks at 0.91 ppm (dd, $J$ = 11.6, 6.6 Hz, 6H) for compound **2**, 1.34 and 1.29 ppm (s, 6H) for compound **3**, 1.64 and 1.56 ppm (s, 6H) for compound **4**.

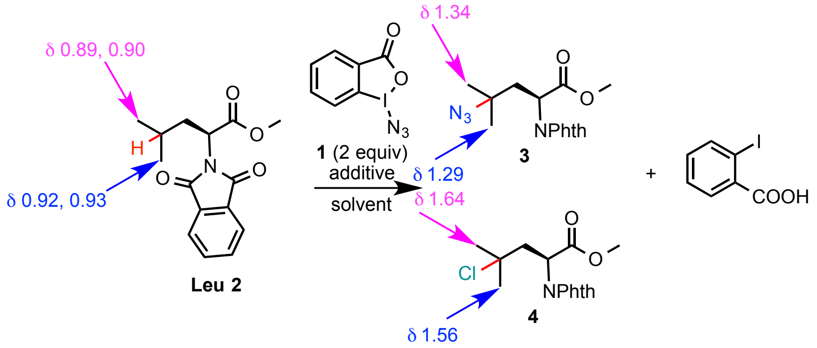


| Entry | Regents (equiv) / atmosphere | Solvent | t (°C) | Time (h) | Yield (%)[a] | |
|---|---|---|---|---|---|---|
| | | | | | **3** | **4** |
| 1 | CAN (2.0), Ar | HFIP | 80 | 24 | 70 | 0 |
| 2 | CAN (2.0), Ar | HFIP | rt | 24 | 0 | 0 |
| 3 | DDQ (2.0), Ar | HFIP | 80 | 24 | <5 | 0 |
| 4 | CuI (2.0), Ar | HFIP | 80 | 24 | <5 | 0 |
| 5 | Mg (2.0), Ar | HFIP | rt | 24 | <5 | 0 |
| 6 | Zn (2.0), Ar | HFIP | rt | 24 | <5 | 0 |

| | | | | | | |
|---|---|---|---|---|---|---|
| 7 | $Ru(bbpy)_3Cl_2$ (0.00001), VL, Ar | HFIP | rt | 24 | 50 | <5 |
| 8 | $Ru(bbpy)_3Cl_2$ (0.000001), VL, Ar | HFIP | rt | 24 | <5 | <5 |
| 9 | $Ru(bbpy)_3Cl_2$ (0.3), VL, Ar | HFIP | rt | 24 | <5 | 56 |
| 10 | $Ru(bbpy)_3Cl_2$ (0.001), NaCl (2.0), VL, Ar | HFIP | rt | 24 | 90 | <5 |
| 11 | $Ru(bbpy)_3Cl_2$ (0.001), LiCl (2.0), VL, Ar | HFIP | rt | 24 | <2 | 90 |
| 12 | LiCl (2.0), VL, Ar | HFIP | rt | 24 | 0 | 0 |
| 13[b] | $Fe(OAc)_2$ (0.1), *i*Pr-PyBOX (0.1) | HFIP | 50 | 24 | 15 | 0 |
| 14[b] | $Fe(OAc)_2$ (0.1), *i*Pr-PyBOX (0.1) | HFIP | rt | 24 | 0 | 0 |
| 15[b] | $Fe(OAc)_2$ (0.1) | HFIP | 50 | 24 | 14 | 0 |

a) Yield are based on $^1$H-NMR analysis on a 0.2 mmol scale. b) Reactions were carried out on a 0.2 mmol scale at 0.2 M concentration.

**Table S1. 3$^o$ C−H azidation and chlorination of Leu 2**

## 5. General procedures and substrate scope for C-H azidation

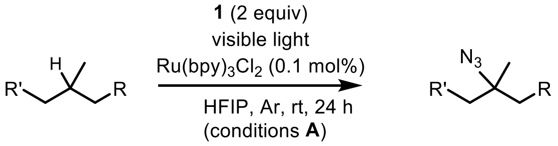


**General conditions A**: Substrates (0.2 mmol, 1.0 equiv) were first dispersed in 0.1 mL of 0.002 M $Ru(bpy)_3Cl_2$ solution in HFIP and stirred for 5 min at rt. Reagent **1** (115.6 mg, 0.4 mmol, 2.0 equiv) was then added. The reaction vial was purged with Ar for 1 min and the reaction mixture vigorously stirred at rt under the fluorescent light irradiation for 24 h. The solvents were removed *in vacuo* and the residue was purified by silica gel flash chromatography to give the desired products.

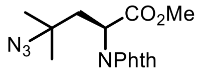


**3** $R_f$ = 0.4, 2% acetone in Hexane

Compound **3** was isolated in 86% yield following the general conditions **A** ($^1$H NMR yield: 91%). **IR** (film) 3000, 2951, 2931, 2104, 1746, 1716, 1389, 725 cm$^{-1}$; **$^1$H NMR** (400 MHz, $CDCl_3$) δ 7.91-7.83 (m, 2H), 7.78-7.70 (m, 2H), 5.05 (d, $J$ = 10.0 Hz, 1H), 3.72 (s, 3H), 2.57-2.47 (m, 1H), 2.45-2.35 (m, 1H), 1.37 (s, 3H), 1.30 (s, 3H); **$^{13}$C NMR** (101 MHz, $CDCl_3$) δ 169.68, 167.54, 134.20, 131.79, 123.56, 60.15, 53.06, 48.44, 38.85, 26.72, 25.18;

**HRMS** Calcd for $C_{15}H_{17}N_2O_4$ [M-$N_2$+$H^+$]: 289.1183; Found: 289.1188.

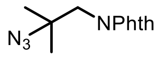


**5** $R_f$= 0.3, 2% acetone in Hexane

Compound **5** was isolated in 35% yield following the general conditions **A** ($^1$H NMR yield: 41%). **IR** (film) 3000, 2800, 2104, 1772, 1717, 1386, 1079, 725 cm$^{-1}$; **$^1$H NMR** (400 MHz, $CDCl_3$) δ 7.89-7.86 (m, 2H), 7.75-7.73 (m, 2H), 3.73 (s, 2H), 1.34 (s, 6H); **$^{13}$C NMR** (101 MHz, $CDCl_3$) δ 168.33, 134.16, 131.82, 123.49, 61.83, 46.25, 24.67; **HRMS** Calcd for $C_{12}H_{12}N_4NaO_2$ [M+$Na^+$]: 267.0852; Found:267.0852.

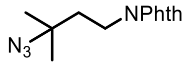


**6** $R_f$= 0.3, 2% acetone in Hexane

Compound **6** was isolated in 80% yield following the general conditions **A** ($^1$H NMR yield: 95%). **IR** (film) 3000, 2927, 2855, 2098, 1772, 1715, 1399, 1373, 719 cm$^{-1}$; **$^1$H NMR** (400 MHz, $CDCl_3$) δ 7.87-7.80 (m, 2H), 7.74-7.67 (m, 2H), 3.82-3.72 (m, 2H), 1.90-1.80 (m, 2H), 1.36 (s, 6H); **$^{13}$C NMR** (101 MHz, $CDCl_3$) δ 168.10, 133.91, 132.07, 123.18, 60.13, 39.13, 33.75, 25.88; **HRMS** Calcd for $C_{13}H_{14}N_4NaO_2$ [M+$Na^+$]: 281.1009; Found:281.1007.

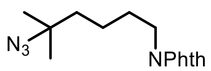


**7** $R_f$= 0.2, 2% acetone in Hexane

Compound **7** was isolated in 85% yield following the general conditions **A** ($^1$H NMR yield: 90%). **IR** (film) 3000, 2971, 2942, 2868, 2096, 1772, 1714, 1438, 1400, 1370, 720 cm$^{-1}$; **$^1$H NMR** (400 MHz, $CDCl_3$) δ7.85-7.82 (m, 2H), 7.75-7.65 (m, 2H), 3.68 (t, $J$ = 7.3 Hz, 2H), 1.71-1.64 (m, 2H), 1.55-1.48 (m, 2H), 1.45-1.35 (m, 2H), 1.24 (s, 6H); **$^{13}$C NMR** (101 MHz, $CDCl_3$) δ 168.30, 133.83, 132.02, 123.11, 61.36, 40.83, 37.64, 28.70, 25.83, 21.45; **HRMS** Calcd for $C_{15}H_{19}N_2O_2$ [M-$N_2$+$H^+$]: 259.1141; Found: 259.1147.

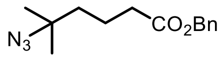


**8** $R_f$= 0.6, 1% EtOAc in Hexane

Compound **8** was isolated in 77% yield following the general conditions **A** ($^1$H NMR yield:

93%). **IR** (film) 3000, 2930, 2098, 1740, 1457, 1370, 1163, 735, 698 cm$^{-1}$; **$^1$H NMR** (400 MHz, $CDCl_3$) δ 7.40-7.30 (m, 5H), 5.12 (s, 2H), 2.38 (t, $J$ = 7.4 Hz, 2H), 1.76-1.68 (m, 2H), 1.51-1.47 (m, 2H), 1.26 (s, 6H); **$^{13}$C NMR** (101 MHz, $CDCl_3$) δ 173.06, 135.95, 128.55, 128.24, 66.22, 61.29, 40.69, 34.19, 25.85, 19.76; **HRMS** Calcd for $C_{14}H_{19}N_3NaO_2$ [M+Na$^+$]: 284.1369; Found:284.1375.

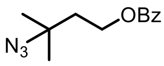


**9** R$_f$= 0.3, 1% acetone in Hexane

Compound **9** was isolated in 77% yield following the general conditions **A** ($^1$H NMR yield: 93%). **IR** (film) 3008, 2973, 2933, 2102, 1720, 1275, 1114, 712 cm$^{-1}$; **$^1$H NMR** (400 MHz, $CDCl_3$) δ 8.03 (d, $J$ = 7.7 Hz, 2H), 7.56 (t, $J$ = 7.3 Hz, 1H), 7.44 (t, $J$ = 7.5 Hz, 2H), 4.44 (t, $J$ = 6.7 Hz, 2H), 1.98 (t, $J$ = 6.7 Hz, 2H), 1.37 (s, 6H); **$^{13}$C NMR** (101 MHz, $CDCl_3$) δ 166.43, 132.96, 130.11, 129.51, 128.35, 61.21, 60.22, 39.71, 26.34; **HRMS** Calcd for $C_{12}H_{15}N_3NaO_2$ [M+Na$^+$]: 256.1056; Found: 256.1057.

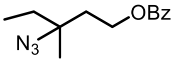


**10** R$_f$= 0.2, 1% acetone in Hexane

Compound **10** was isolated in 61% yield following the general conditions **A** ($^1$H NMR yield: 68%). **IR** (film) 3004, 2972, 2932, 2097, 1721, 1272, 1113, 712 cm$^{-1}$; **$^1$H NMR** (400 MHz, $CDCl_3$) δ 8.04 (d, $J$ = 7.6 Hz, 2H), 7.56 (t, $J$ = 7.3 Hz, 1H), 7.44 (t, $J$ = 7.5 Hz, 2H), 4.43 (t, $J$ = 6.9 Hz, 2H), 2.07-1.88 (m, 2H), 1.65 (q, $J$ = 7.4 Hz, 2H), 1.34 (s, 3H), 0.98 (t, $J$ = 7.5 Hz, 3H); **$^{13}$C NMR** (101 MHz, $CDCl_3$) δ 166.48, 132.97, 130.08, 129.51, 128.36, 63.12, 61.11, 37.17, 32.47, 23.05, 8.35; **HRMS** Calcd for $C_{13}H_{17}N_3NaO_2$ [M+Na$^+$]: 270.1213; Found: 270.1215

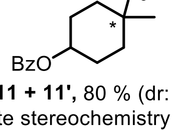


**11 + 11'**, 80 % (dr: ~1:1)
absolute stereochemistry unassigned

**11':** R$_f$= 0.51, 5% EtOAc in Hexane

**11'':** R$_f$= 0.5, 5% EtOAc in Hexane

Compound **11'** was isolated in 40% yield following the general conditions **A** ($^1$H NMR yield: 45%). **IR** (film) 3100, 2951, 2931, 2856, 2110, 2088, 1717, 1273, 1237, 1110, 712 cm$^{-1}$; **$^1$H**

**NMR** (400 MHz, $CDCl_3$) δ 8.03 (d, *J* = 7.6 Hz, 2H), 7.57 (t, *J* = 7.2 Hz, 1H), 7.45 (t, *J* = 7.2 Hz, 2H), 5.24 (br s, 1H), 1.93-1.63 (m, 8H), 1.39 (s, 3H); **$^{13}$C NMR** (101 MHz, $CDCl_3$) δ 165.75, 132.87, 130.70, 129.44, 128.35, 69.40, 60.88, 31.71, 26.42, 26.24; **HRMS** Calcd for $C_{14}H_{17}N_3NaO_2$ [M+Na$^+$]: 282.1213; Found:282.1214.

Compound **11''** was isolated in 40% yield following the general conditions **A** ($^1$H NMR yield: 46%). **IR** (film) 3100, 2942, 2870, 2097, 1716, 1276, 1253, 1114, 712 cm$^{-1}$; **$^1$H NMR** (400 MHz, $CDCl_3$) δ 8.04 (d, *J* = 7.6 Hz, 2H), 7.55 (t, *J* = 7.2 Hz, 1H), 7.44 (t, *J* = 7.2 Hz, 2H), 5.00-4.93 (m, 1H), 1.94-1.80 (m, 6H), 1.57-1.50 (m, 2H), 1.36 (s, 3H); **$^{13}$C NMR** (101 MHz, $CDCl_3$) δ 165.98, 132.86, 130.53, 129.53, 128.29, 71.96, 60.33, 34.27, 27.25, 25.91; **HRMS** Calcd for $C_{14}H_{17}N_3NaO_2$ [M+Na$^+$]: 282.1213; Found:282.1219.

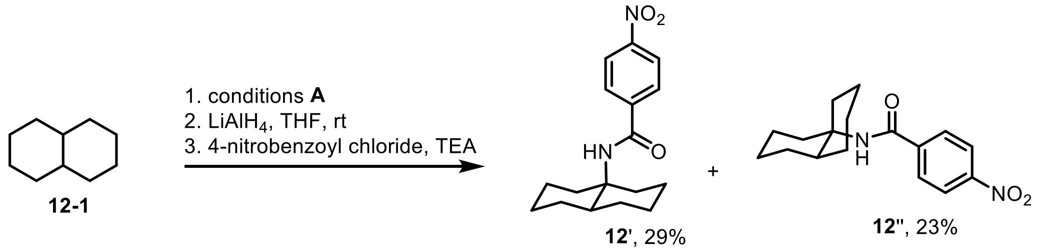


**Scheme S12**

*Cis*-Decahydronaphthalene **12-1** (0.2 mmol) were first dispersed in 0.1 mL of 0.002 M $Ru(bpy)_3Cl_2$ solution in HFIP and stirred for 5 min at rt before reagent **1** (115.6 mg, 0.4 mmol) was added. The resulting mixture was purged with Ar for 1 min and vigorously stirred at rt under light irradiation for 24 h. The solvent was removed under reduced pressure and the resulting residue were filtered through a short silica gel column and eluted with hexane (50 mL). The eluted solution was concentrated *in vacuo*, the resulting residue was dissolved in 15 mL of anhydrous THF. To the solution was added $LiAlH_4$ (100 mg) at 0 °C, the reaction mixture was stirred at rt for 30 min and then quenched with saturated aq. $NH_4Cl$ and the mixture was extracted with $CH_2Cl_2$ (3 x 20.0 mL). The organic layer was dried over anhydrous $Na_2SO_4$, concentrated under reduced pressure, then the crude product was redissolved in 10 mL of anhydrous $CH_2Cl_2$. To the solution was added 4-nitrobenzoyl chloride (111 mg, 0.6 mmol) at 0 °C, the mixture was stirred at rt overnight. The solvent was removed under reduced pressure and the resulting residue were purified by silica gel column chromatography (eluted with hexane/EtOAc (v/v 20:1)) to give products **12'** and **12''** (known

compounds).[1] Compound **12'** was isolated in 29% yield. **$^{1}$H NMR** (400 MHz, $CDCl_3$) δ 8.30 (d, *J* = 8.7 Hz, 2H), 7.90 (d, *J* = 8.7 Hz, 2H), 5.75 (b, 1H), 2.86-2.74 (m, 2H), 1.80-1.70 (m, 2H), 1.63-1.31 (m, 9H), 1.28-1.08 (m, 4H). Compound **12''** was isolated in 23% yield. **$^{1}$H NMR** (400 MHz, $CDCl_3$) δ 8.27 (d, *J* = 8.7 Hz, 2H), 7.88 (d, *J* = 8.7 Hz, 2H), 5.90 (b, 1H), 2.10 (b, 2H), 1.96 (b, 2H), 1.74-1.48 (m, 10H), 1.46-1.33 (m, 3H). Spectra data are consistent with those reported in the literature.

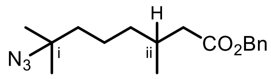


**13**

($C_{ii}$-azidated product <3%) $R_f$ = 0.3, 1% EtOAc in Hexane

Compound **13** was isolated in 73% yield following the general conditions **A** ($^{1}$H NMR yield: 90%, Cii-azidated product <3%). **IR** (film) 3005, 2930, 2097, 1735, 1457, 1370, 1163 $cm^{-1}$; **$^{1}$H NMR** (400 MHz, $CDCl_3$) δ 7.44-7.28 (m, 5H), 5.12 (s, 2H), 2.35 (dd, *J* = 14.8, 6.3 Hz, 1H), 2.19 (dd, *J* = 14.8, 7.8 Hz, 1H), 2.05-1.96 (m, 1H), 1.48-1.14 (m, 12H), 0.95 (d, *J* = 6.6 Hz, 3H); **$^{13}$C NMR** (101 MHz, $CDCl_3$) δ 172.94, 136.05, 128.51, 128.21, 128.16, 66.04, 61.55, 41.74, 41.43, 36.73, 30.24, 25.97, 25.92, 21.51, 19.63; **HRMS** Calcd for $C_{17}H_{25}N_3NaO_2$ [M+H$^+$]: 326.1839; Found:326.1846.

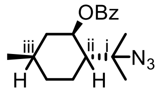


**14**, 43%

(+ $C_i$,$C_{iii}$-diazidated **14d**, 18%)

**14:** $R_f$ = 0.65, 10% acetone in Hexane

**14d:** $R_f$ = 0.64, 10% acetone in Hexane

Compound **14** was isolated in 43% yield following the general conditions **A** ($^{1}$H NMR yield: 52%). **$^{1}$H NMR** (400 MHz, $CDCl_3$) δ 8.07 (dd, *J* = 1.2, 8.2 Hz, 2H), 7.56 (t, *J* = 7.6 Hz, 1H), 7.45 (t, *J* = 7.6 Hz, 2H), 5.10 (td, *J* = 4.4, 10.8 Hz, 1H), 2.12-2.07 (m, 1H), 2.03-1.98 (m, 1H), 1.88-1.82 (m, 1H), 1.78-1.74 (m, 1H), 1.64-1.53 (m, 1H), 1.29 (s, 3H), 1.28 (s, 3H), 1.22-1.10 (m, 3H), 0.92 (d, *J* = 6.8 Hz, 3H); **$^{13}$C NMR** (101 MHz, $CDCl_3$) δ 165.68, 132.89, 130.58, 129.60, 128.36, 73.84, 63.57, 49.11, 41.29, 34.05, 31.19, 26.52, 25.13, 24.47, 21.69; **HRMS** Calcd for $C_{17}H_{23}N_3NaO_2$ [M+Na$^+$]: 324.1682; Found:324.1689.

Compound **14d** was isolated in 18% yield following the general conditions **A** ($^{1}$H NMR yield: 29%). **$^{1}$H NMR** (400 MHz, $CDCl_3$) δ 8.05 (dd, *J* = 1.2, 8.4 Hz, 2H), 7.56 (t, *J* = 7.6 Hz, 1H), 7.44 (t, *J* = 7.6 Hz, 2H), 5.33 (td, *J* = 4.4, 10.8 Hz, 1H), 2.24-2.19 (m, 1H), 1.94-1.83 (m, 3H), 1.60-1.38 (m, 4H), 1.37 (s, 3H), 1.32 (s, 3H), 1.31 (s, 3H); **$^{13}$C NMR** (101 MHz, $CDCl_3$) δ

165.33, 133.04, 130.25, 129.62, 128.41, 70.73, 63.40, 61.84, 48.75, 42.47, 35.81, 26.37, 25.13, 24.25, 22.47; **HRMS** Calcd for $C_{17}H_{23}N_6O_2$ [M+H$^+$]: 343.1877; Found:343.1873

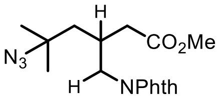


**15** R$_f$= 0.4, 2% acetone in Hexane

Compound **15** was isolated in 77% yield following the general conditions **A** ($^1$H NMR yield: 85%). **$^1$H NMR** (400 MHz, CDCl3) δ 7.87-7.81 (m, 2H), 7.75-7.68 (m, 2H), 3.83 (dd, *J* = 13.8, 5.5 Hz, 1H), 3.63 (dd, *J* = 13.8, 8.5 Hz, 1H), 3.57 (s, 3H), 2.59-2.48 (m, 1H), 2.40 (t, *J* = 6.3 Hz, 2H), 1.60-1.51 (m, 2H), 1.34 (d, *J* = 16.3 Hz, 6H). **$^{13}$C NMR** (101 MHz, CDCl3) δ 172.49, 168.57, 134.02, 131.88, 123.26, 61.11, 51.54, 43.05, 42.32, 37.86, 31.28, 26.27, 26.18. **HRMS** Calcd for $C_{18}H_{21}N_3NaO_4$ [M+H$^+$]: 367.1377; Found:367.1383.

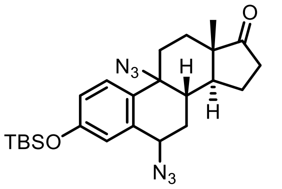


**16** R$_f$= 0.5, 30% EtOAc in Hexane (absolute stereochemistry unassigned)

Compound **16** was isolated in 32% yield following the general conditions **A** ($^1$H NMR yield: 37%). **$^1$H NMR** (400 MHz, $CDCl_3$) δ 7.19 (d, *J* = 8.8 Hz, 1H), 6.79 (dd, *J* = 2.0, 8.8 Hz, 1H), 6.68 (d, *J* = 2.0, 1H), 4.53 (t, *J* = 2.8 Hz, 1H), 2.91-2.84 (m, 2H), 2.57-2.50 (m, 1H), 2.18-2.10 (m, 4H), 1.99-1.92 (m, 1H), 1.86-1.60 (m, 4H), 1.12 (s, 3H), 0.98 (s, 9H), 0.21 (s, 6H); **$^{13}$C NMR** (101 MHz, $CDCl_3$) δ 217.42, 155.60, 139.29, 127.30, 125.45, 120.98, 118.04, 68.51, 61.53, 46.54, 43.02, 37.19, 35.18, 31.47, 29.25, 25.60, 20.95, 20.75, 18.14, 14.78, -4.38; **$^{13}$C NMR (DEPT 135)** (101 MHz, $CDCl_3$) δ 127.30, 120.97, 118.04, 61.51, 43.01, 37.18, 35.18, 31.45, 29.24, 25.60, 20.95, 20.75, 14.77, -4.36; **$^{13}$C NMR (DEPT 90)** ( 101 MHz, $CDCl_3$) δ 127.34, 121.02, 118.08, 61.55, 43.05, 37.22; **HRMS** Calcd for $C_{24}H_{35}N_2O_2Si$ [M-2N$_2$+H$^+$]: 411.2462; Found: 411.2468.

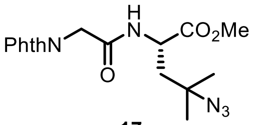


**17** R$_f$= 0.2, 30% EtOAc in Hexane

Compound **17** was isolated in 64% yield following the general conditions **A** ($^1$H NMR yield: 70%). **$^1$H NMR** (400 MHz, $CDCl_3$) δ 7.90-7.88 (m, 2H), 7.76-7.74 (m, 2H), 6.57 (d, *J* = 6.4

Hz, 1H), 4.65-4.60 (m, 1H), 4.40 (d, $J$ = 2.8 Hz, 2H), 3.74 (s, 3H), 2.01-1.96 (m, 1H), 1.89-1.83 (m, 1H), 1.34 (d, $J$ = 1.6 Hz, 3H), 1.33 (d, $J$ = 1.6 Hz, 3H); **$^{13}$C NMR** (101 MHz, $CDCl_3$) δ 172.14, 172.07, 167.58, 165.96, 165.90, 134.27, 131.90, 123.65, 123.63, 60.39, 52.65, 50.10, 50.04, 42.19, 40.70, 26.38, 26.31, 25.65, 25.61; **HRMS** Calcd for $C_{17}H_{19}N_5NaO_5$ [M+Na$^+$]: 396.1278; Found: 396.1284.

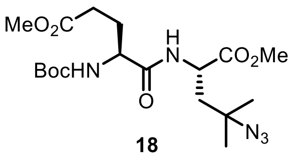


**18** $R_f$= 0.2, 30% EtOAc in Hexane

Compound **18** was isolated in 30% yield following the general conditions **A** ($^1$H NMR yield: 33%). **$^1$H NMR** (400 MHz, $CDCl_3$) δ 7.03 (d, $J$ = 6.8 Hz, 1H), 6.25 (d, $J$ = 8.0 Hz, 1H), 4.61 (td, $J$ = 4.4, 8.0 Hz, 1H), 4.23-4.17 (m, 1H), 3.73 (s, 3H), 3.68 (s, 3H), 2.55-2.41 (m, 2H), 2.18-2.09 (m, 1H), 2.03-1.80 (m, 3H), 1.42 (s, 9H), 1.33 (s, 6H); **$^{13}$C NMR** (101 MHz, $CDCl_3$) δ 173.87, 172.17, 171.23, 155.53, 80.07, 60.30, 53.42, 52.55, 51.85, 49.67, 42.39, 30.10, 28.21, 27.88, 26.24, 25.84; **HRMS** Calcd for $C_{18}H_{31}N_5NaO_7$ [M+Na$^+$]: 452.2116; Found: 452.2121.

## 6. General procedures and substrate scope for C-H chlorination

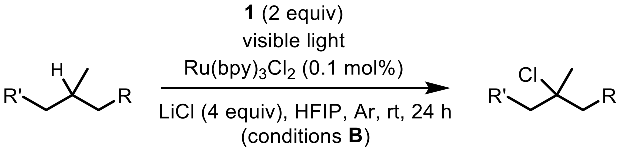


**General conditions B:** Substrates (0.2 mmol, 1.0 equiv) and LiCl (0.8 mmol, 4.0 equiv) were first dispersed in 0.1 mL of 0.002 M $Ru(bpy)_3Cl_2$ solution in HFIP and stirred for 5 min at rt before reagent **1** (115.6 mg, 0.4 mmol, 2.0 equiv) was added. The reaction vial was purged with Ar for 1 min and the reaction mixture vigorously stirred at rt under the fluorescent light irradiation for 24 h. The solvents were removed *in vacuo*, the residue was purified by silica gel flash chromatography to give the desired products.

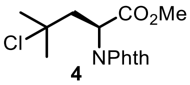


**4** $R_f$= 0.5, 2% acetone in Hexane

Compound **4** was isolated in 77% yield following the general conditions **B** ($^1$H NMR yield:

95%). **IR** (film) 3000, 2957, 2931, 1746, 1716, 1400, 1380, 1719, 735 $cm^{-1}$; **$^1$H NMR** (400 MHz, $CDCl_3$) δ 7.92-7.83 (m, 2H), 7.79-7.70 (m, 2H), 5.22 (d, *J* = 9.4 Hz, 1H), 3.73 (s, 3H), 2.87-2.68 (m, 2H), 1.66 (s, 3H), 1.57 (s, 3H); **$^{13}$C NMR** (101 MHz, $CDCl_3$) δ 169.67, 167.60, 134.21, 131.90, 123.60, 68.13, 53.11, 49.24, 42.87, 33.20, 31.91; **HRMS** Calcd for $C_{15}H_{17}ClNO_4$ [M+H$^+$]: 310.0841; Found:310.0847.

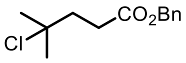


**19** $R_f$= 0.2, 1% EtOAc in Hexane

Compound **19** was isolated in 46% yield following the general conditions **B** ($^1$H NMR yield: 61%). **IR** (film) 3004, 2973, 2927, 1737, 1157, 734, 698 $cm^{-1}$; **$^1$H NMR** (400 MHz, $CDCl_3$) δ 7.42-7.30 (m, 5H), 5.13 (s, 2H), 2.69-2.55 (m, 2H), 2.16-2.05 (m, 2H), 1.58 (s, 6H); **$^{13}$C NMR** (101 MHz, $CDCl_3$) δ 173.01, 135.80, 128.56, 128.27, 69.53, 66.42, 40.30, 32.31, 30.40; **HRMS** Calcd for $C_{13}H_{17}ClNaO_2$ [M+H$^+$]: 263.0809; Found:263.0806.

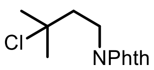


**20** $R_f$= 0.5, 2% acetone in Hexane

Compound **20** was isolated in 62% yield following the general conditions **B** ($^1$H NMR yield: 80%). **IR** (film) 3000, 2994, 2947, 1770, 1713, 1401, 722 $cm^{-1}$; **$^1$H NMR** (400 MHz, $CDCl_3$) δ 7.87-7.79 (m, 2H), 7.75-7.65 (m, 2H), 3.99-3.84 (m, 2H), 2.19-2.06 (m, 2H), 1.65 (s, 6H); **$^{13}$C NMR** (101 MHz, $CDCl_3$) δ 168.12, 133.91, 132.10, 123.19, 68.03, 43.27, 34.58, 32.43; **HRMS** Calcd for $C_{13}H_{15}ClNO_2$ [M+H$^+$]: 252.0786; Found: 252.0787.

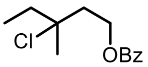


**21** $R_f$= 0.4, 1% acetone in Hexane

Compound **21** was isolated in 75% yield following the general conditions **B** ($^1$H NMR yield: 80%). **IR** (film) 3005, 2974, 2930, 1720, 1273, 1113, 711 $cm^{-1}$; **$^1$H NMR** (400 MHz, $CDCl_3$) δ 8.03 (d, *J* = 7.7 Hz, 2H), 7.56 (t, *J* = 7.3 Hz, 1H), 7.44 (t, *J* = 7.5 Hz, 2H), 4.56 (t, *J* = 6.8 Hz, 2H), 2.35-2.11 (m, 2H), 1.96-1.79 (m, 2H), 1.61 (s, 3H), 1.06 (t, *J* = 7.3 Hz, 3H); **$^{13}$C NMR** (101 MHz, $CDCl_3$) δ 166.48, 132.96, 130.14, 129.52, 128.36, 72.73, 61.88, 41.78,

37.39, 29.58, 9.13; **HRMS** Calcd for $C_{13}H_{18}ClO_2$ [M+H$^+$]: 241.0990; Found: 241.0987.

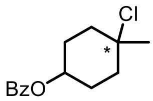


**22'+22''**, 93% (dr: ~1:1)

**22'** (trans): $R_f$= 0.4, 1% acetone in Hexane

**22''**(cis): $R_f$= 0.39, 1% acetone in Hexane

The stereochemistry was determined by comparison with the similar reported structure.[14]

Compound **22'** was isolated in 40% yield following the general conditions **B**. **IR** (film) 3000, 2942, 2923, 2850, 1716, 1275, 1113, 711 cm$^{-1}$; **$^1$H NMR** (400 MHz, $CDCl_3$) δ 8.06 (d, *J* = 7.4 Hz, 2H), 7.56 (t, *J* = 7.3 Hz, 1H), 7.44 (t, *J* = 7.6 Hz, 2H), 5.02-4.88 (m, 1H), 2.16-1.89 (m, 6H), 1.79-1.59 (m, 5H); **$^{13}$C NMR** (101 MHz, $CDCl_3$) δ 166.05, 132.86, 130.55, 129.57, 128.30, 72.27, 70.11, 39.43, 32.97, 27.64; **HRMS** Calcd for $C_{14}H_{18}ClO_2$ [M+H$^+$]: 253.0990; Found: 253.0994.

Compound **22''** was isolated in 53% yield following the general conditions **B**. **IR** (film) 3004, 2929, 2870, 2854, 1717, 1450, 1272, 1109, 712 cm$^{-1}$; **$^1$H NMR** (400 MHz, $CDCl_3$) δ 8.02 (d, *J* = 7.2 Hz, 1H), 7.57 (t, *J* = 7.4 Hz, 1H), 7.45 (t, *J* = 7.6 Hz, 2H), 5.34-5.23 (m, 1H), 2.19-2.04 (m, 2H), 2.01-1.86 (m, 6H), 1.70 (s, 3H); **$^{13}$C NMR** (101 MHz, $CDCl_3$) δ 166.05, 132.86, 130.55, 129.57, 128.30, 72.27, 70.11, 39.43, 32.97, 27.64; **HRMS** Calcd for $C_{14}H_{18}ClO_2$ [M+H$^+$]: 253.0990; Found: 253.0993.

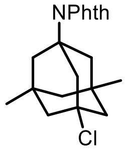


**23** $R_f$= 0.3, 1% acetone in Hexane

Compound **23** was isolated in 63% yield following the general conditions **B**. **IR** (film) 3000, 2954, 2907, 1704, 1317, 714 cm$^{-1}$; **$^1$H NMR** (400 MHz, $CDCl_3$) δ 7.81-7.72 (m, 2H), 7.71-7.63 (m, 2H), 2.75 (s, 2H), 2.14 (dd, *J* = 31.9, 12.3 Hz, 4H), 1.82 (dd, *J* = 48.8, 11.9 Hz, 4H), 1.21 (dd, *J* = 25.1, 13.8 Hz, 2H), 0.97 (s, 6H); **$^{13}$C NMR** (101 MHz, $CDCl_3$) δ 169.37, 133.88, 131.67, 122.70, 67.17, 62.24, 52.32, 48.62, 47.84, 44.47, 35.22, 29.28; **HRMS** Calcd for $C_{20}H_{23}ClNO_2$ [M+H$^+$]: 344.1412; Found: 344.1417.

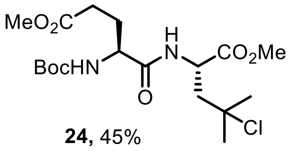

**24**, 45%
(34% of SM recovered) $R_f$= 0.4, 3% EtOAc in Hexane

Compound **24** was isolated in 45% yield following the general conditions **B** ($^{1}$H NMR yield: 59%). **$^{1}$H NMR** (400 MHz, $CDCl_3$) δ 7.01 (d, *J* = 7.6 Hz, 1H), 5.28 (d, *J* = 7.2 Hz, 1H), 4.76 (td, *J* = 3.6, 8.0 Hz, 1H), 4.22-4.16 (m, 1H), 3.72 (s, 3H), 3.68 (s, 3H), 2.53-2.37 (m, 3H), 2.17-2.08 (m, 2H), 1.96-1.87 (m, 1H), 1.61 (s, 6H), 1.41 (s, 9H); **$^{13}$C NMR** (101 MHz, $CDCl_3$) δ 173.91, 172.15, 171.24, 155.57, 80.08, 68.22, 53.45, 52.60, 51.85, 50.13, 46.89, 32.63, 32.46, 30.12, 28.23, 27.75; **HRMS** Calcd for $C_{18}H_{31}ClN_2NaO_7$ [M+H$^+$]: 445.1712; Found: 445.1717.

## 7. General procedures and substrate scope for C-H bromination

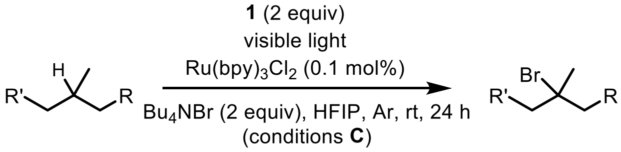


**Conditions C:** Substrates (0.2 mmol, 1.0 equiv) and $Bu_4NBr$ (0.2 mmol, 1.0 equiv) were first dispersed in 0.1 mL of 0.002 M $Ru(bpy)_3Cl_2$ solution in HFIP and stirred for 5 min at rt. Reagent **1** (115.6 mg, 0.4 mmol, 2.0 equiv) was then added. The resulting mixture was purged with Ar for 1 min and vigorously stirred at rt under the fluorescent light irradiation for 2 h. Another equiv of $Bu_4NBr$ (0.2 mmol) was added, the reaction mixture was stirred at rt for 22 h. The solvents were removed *in vacuo*, the resulting residue was purified by silica gel flash chromatography.

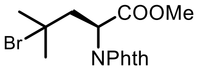

**25** $R_f$= 0.4, 2% acetone in Hexane

Compound **25** was isolated in 71% yield following the general conditions **C** ($^{1}$H NMR yield: 80%). **$^{1}$H NMR** (400 MHz, $CDCl_3$) δ 7.92-7.82 (m, 2H), 7.79-7.68 (m, 2H), 5.23 (dd, *J* = 8.4, 3.2 Hz, 1H), 3.73 (s, 3H), 2.93-2.75 (m, 2H), 1.82 (s, 3H), 1.74 (s, 3H). **$^{13}$C NMR** (101 MHz, $CDCl_3$) δ 169.59, 167.63, 134.24, 131.87, 123.63, 63.60, 53.15, 50.13, 44.31, 34.78, 33.83.

**HRMS** Calcd for $C_{15}H_{17}BrNO_4$ [M+H$^+$]: 354.0335; Found: 354.0333.

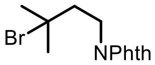


**26** R$_f$= 0.4, 2% acetone in Hexane

Compound **26** was isolated in 70% yield following the general conditions **C** ($^1$H NMR yield: 80%). **IR** (film) 3007, 2926, 2856, 1773, 1715, 1400, 1373, 720 cm$^{-1}$; **$^1$H NMR** (400 MHz, $CDCl_3$) δ 7.90-7.80 (m, 2H), 7.77-7.65 (m, 2H), 4.03-3.85 (m, 2H), 2.26-2.07 (m, 2H), 1.84 (s, 6H); **$^{13}$C NMR** (101 MHz, $CDCl_3$) δ 168.14, 133.95, 132.09, 123.23, 63.56, 44.69, 35.69, 34.24; **HRMS** Calcd for $C_{13}H_{15}BrNO_2$ [M+H$^+$]: 296.0281; Found: 296.0285.

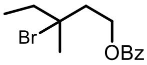


**27** R$_f$= 0.3, 1% acetone in Hexane

Compound **27** was isolated in 62% yield following the general conditions **C** ($^1$H NMR yield: 70%). **$^1$H NMR** (400 MHz, $CDCl_3$) δ 8.04 (d, $J$ = 7.8 Hz, 2H), 7.56 (t, $J$ = 7.4 Hz, 1H), 7.44 (t, $J$ = 7.6 Hz, 2H), 4.58 (t, $J$ = 6.9 Hz, 2H), 2.45-2.33 (m, 1H), 2.33-2.18 (m, 1H), 2.08-1.86 (m, 2H), 1.80 (s, 3H), 1.09 (t, $J$ = 7.3 Hz, 3H). **$^{13}$C NMR** (101 MHz, $CDCl_3$) δ 166.46, 132.98, 130.09, 129.53, 128.37, 70.52, 62.84, 43.03, 38.77, 31.27, 10.27. **HRMS** Calcd for $C_{13}H_{18}BrO_2$ [M+H$^+$]: 285.0485; Found: 285.0488.

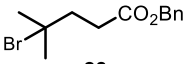


**28** R$_f$= 0.4, 1% EtOAc in Hexane

Compound **28** was isolated in 65% yield following the general conditions **C** ($^1$H NMR yield: 70%). **$^1$H NMR** (400 MHz, $CDCl_3$) δ 7.50-7.27 (m, 5H), 5.13 (s, 2H), 2.73-2.50 (m, 2H), 2.19-2.06 (m, 2H), 1.76 (s, 6H). **$^{13}$C NMR** (101 MHz, $CDCl_3$) δ 172.87, 135.80, 128.57, 128.28, 128.14, 66.46, 66.06, 41.75, 34.13, 31.70. **HRMS** Calcd for $C_{13}H_{18}BrO_2$ [M+H$^+$]: 285.0485; Found: 285.0483.

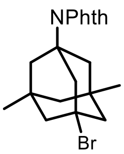


**29** R$_f$= 0.5, 1% acetone in Hexane

Compound **29** was isolated in 65% yield following the general conditions **C**. **$^1$H NMR** (400 MHz, $CDCl_3$) δ 7.75 (d, *J* = 2.7 Hz, 2H), 7.68 (d, *J* = 2.6 Hz, 2H), 2.93 (s, 2H), 2.23 (d, *J* = 12.3 Hz, 2H), 2.12 (dd, *J* = 29.2, 13.1 Hz, 4H), 1.96 (d, *J* = 11.9 Hz, 2H), 1.24 (q, *J* = 13.5 Hz, 3H), 0.96 (s, 6H). **$^{13}$C NMR** (101 MHz, $CDCl_3$) δ 169.33, 133.90, 131.65, 122.71, 62.80, 62.27, 53.76, 49.13, 48.52, 44.40, 35.91, 29.31. **HRMS** Calcd for $C_{20}H_{23}BrNO_2$ [M+H$^+$]: 388.0907; Found: 388.0905.

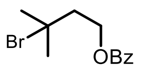

**30** $R_f$ = 0.3, 1% acetone in Hexane

Compound **30** was isolated in 70% yield following the general conditions **C** ($^1$H NMR yield: 75%). **$^1$H NMR** (400 MHz, $CDCl_3$) δ 8.03 (t, *J* = 9.7 Hz, 2H), 7.56 (t, *J* = 7.0 Hz, 1H), 7.44 (t, *J* = 7.2 Hz, 2H), 4.65 (t, *J* = 6.5 Hz, 2H), 2.44 (t, *J* = 6.4 Hz, 2H), 2.00 (s, 6H). $^{13}$C NMR (101 MHz, $CDCl_3$) δ 166.44, 133.00, 130.10, 129.54, 128.38, 64.25, 63.08, 45.43, 34.72. **HRMS** Calcd for $C_{12}H_{16}BrO_2$ [M+H$^+$]: 271.0328; Found: 271.0325.

## 8. Chlorination of 32 with chloroiodane 31

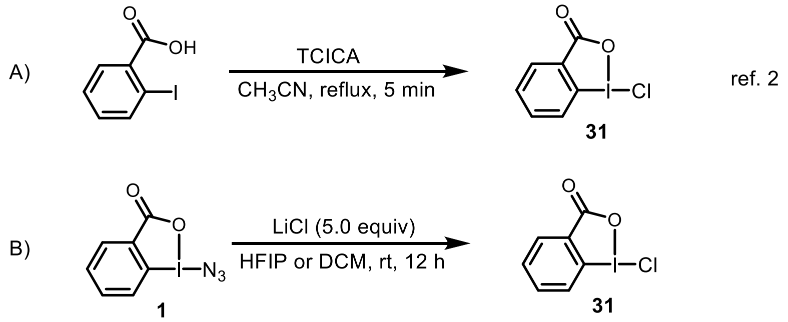


**Scheme S13**

Chloroiodane **31** is a stable white solid and can be easily synthesized from ortho-iodobenzoic acid in one step following Togni's procedure (**Scheme S13 A)**.[2]

**Proof of in situ formation of 31**: We observed that chloroiodane **31** could also be readily formed by mixing **1** with LiCl at rt (Scheme S13B): To a solution of azidoiodane **1** (28.9 mg, 0.1 mmol, 1.0 equiv) in HFIP (0.1 mL) was added LiCl (21 mg, 0.5 mmol, 5.0 equiv) at rt. The reaction mixture was stirred at rt for 12 h in the absence of visible light irradiation. The

solvent was removed under reduced pressure. The $^{1}$H NMR analysis of the resulting sample showed that 42% and >95% yield of chloroiodane **31** was formed in HFIP and DCM respectively (Scheme S14).

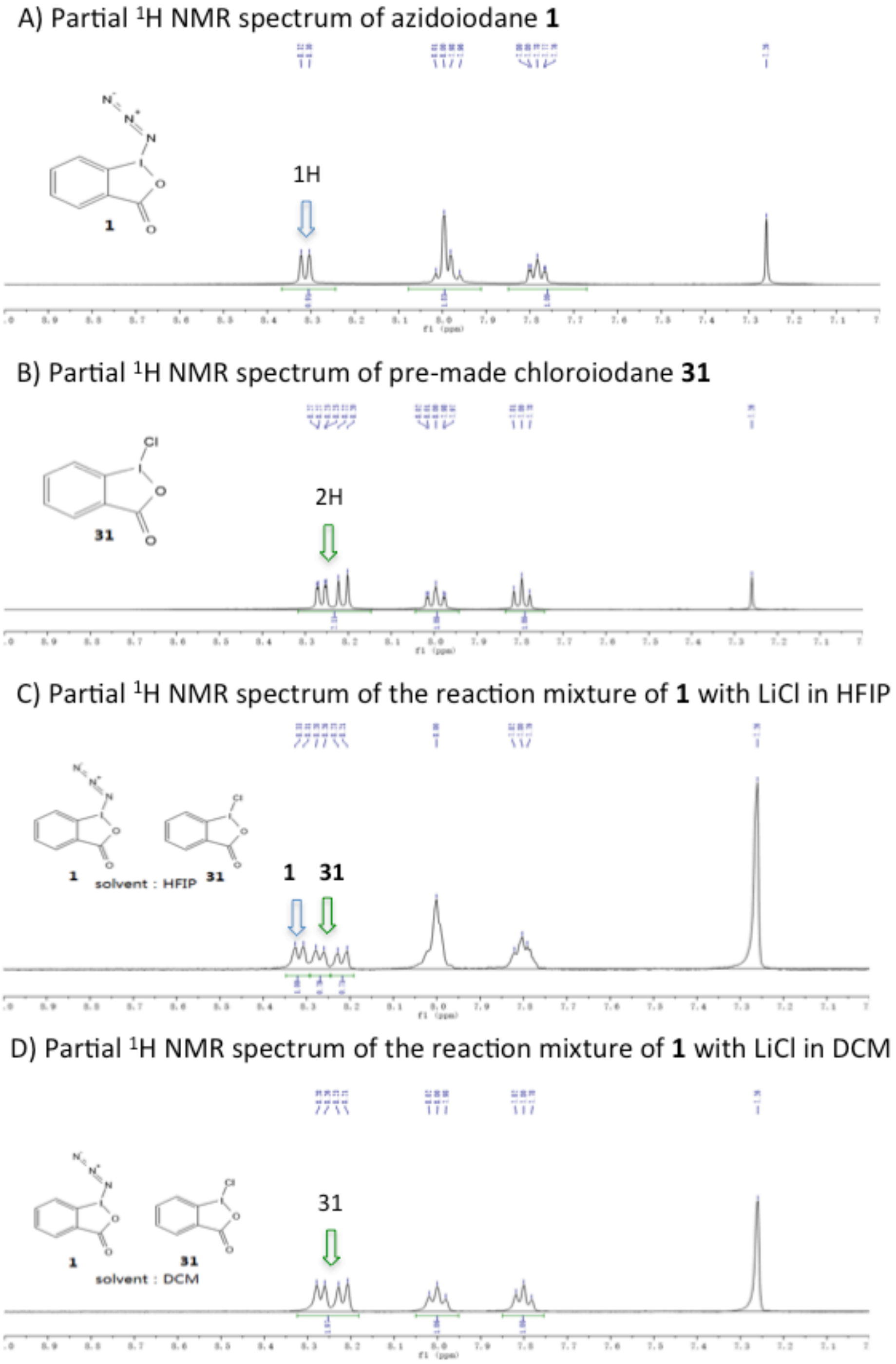


**Scheme S14**. $^{1}$H NMR analysis of substitution reaction of **1** with LiCl at rt

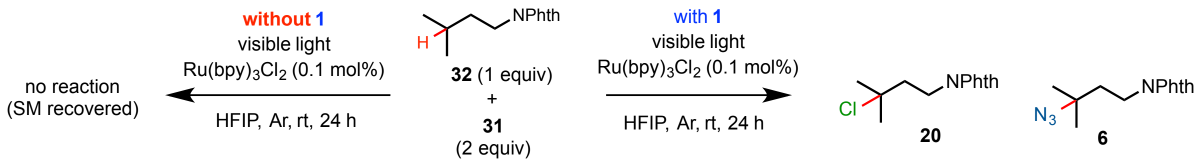


| **1** (equiv) | converstion of **32** | yield of **20** | yield of **6** (by $^1$H-NMR) |
|---|---|---|---|
| 0.2 | 74% | 58% | <2% |
| 0.5 | 97% | 78% | <2% |
| 1 | >98% | 82% (72% isolated) | <2% |

**Scheme S15**. Control reactions of **32** with **1** and **31**

**Control experiments of 32 with azidoiodane 1 and chloroiodane 31**: experiments were performed under the standard conditions A at a 0.2 mmol scale (Scheme S15). Reactions were analyzed by $^1$H-NMR. A 72% yield of the chlorinated product **20** was isolated under the conditions of using 1 equiv of **1**.

## 9. Measurement of quantum yield (Φ) for C-H azidation of 32

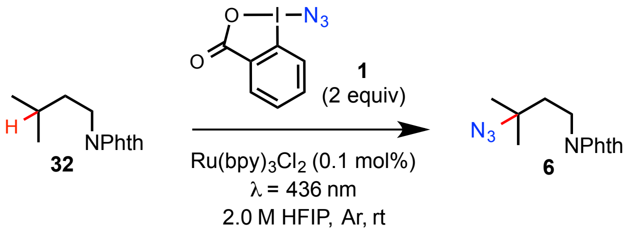


**Scheme S16.** C-H azidation of **32**

**General information:** All the experiments were carried out based on C-H azidation of substrate **32** with azidoiodane **1** following Yoon's procedure.[15] Solutions used were prepared in the dark and reactions were conducted in a 1 cm square quartz cuvette and capped with either a PTFE stopper. A Hitachi F-4600 fluorescence spectrophotometer with a 150 W Xe lamp was used as the light source for the quantum yield measurements. A 12 W compact fluorescent light bulb was used for "light/dark" at a distance of 10 cm away from the reaction flask. UV-vis data were measured on a Hitachi U-3900 spectrophotometer.

The quantum yield can be calculated using eq 1:

$$\Phi = \frac{\text{mols of product formed}}{\text{einsteins of light absorbed}} = \frac{\text{mols of starting material·yield}}{\text{fluxl·t·f}} \quad (1)$$

Where fluxl is the photon flux of the spectrophotometer, t is the reaction time and f is the light absorbance of catalyst.

**A) Absorbance of catalyst:**

The absorbance of $Ru(bpy)_3Cl_2$ in HFIP was measured at the reaction concentration of $2.0 \times 10^{-3}$ M and at a substantially more dilute concentration of $2.0 \times 10^{-4}$ M. The absorbance at 436 nm for a $2.0 \times 10^{-3}$ M solution is >3 (**Figure S1**) indicating the fraction of light absorbed is >0.999.

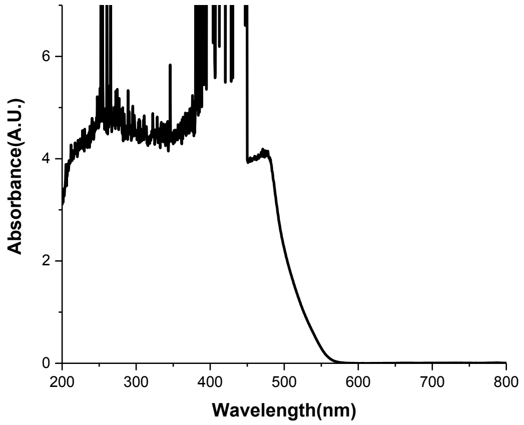


**Figure S1.** Absorbance of a $2.0 \times 10^{-3}$ M solution of $Ru(bpy)_3Cl_2$ in HFIP.

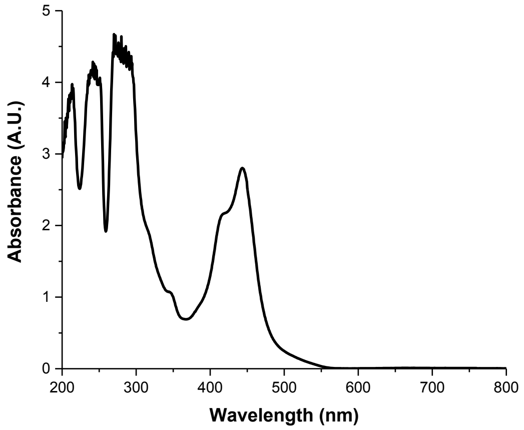


**Figure S2.** Absorbance of a $2.0 \times 10^{-4}$ M solution of $Ru(bpy)_3Cl_2$ in HFIP.

**B) Determination of the light intensity at 436 nm:**

The photon flux of the spectrophotometer was determined by standard ferrioxalate actinometry. A 0.15 M solution of ferrioxalate was prepared by dissolving 2.21 g of potassium ferrioxalate hydrate in 30 mL of 0.05 M $H_2SO_4$. A buffered solution of phenanthroline was prepared by dissolving 50 mg of phenanthroline and 11.25 g of sodium acetate in 50 mL of 0.5 M $H_2SO_4$. Both solutions were stored in the dark. To determine the photon flux of the spectrophotometer, 2.0 mL of the ferrioxalate solution was placed in a cuvette and irradiated for 90.0 seconds at $\lambda$ = 436 nm with an emission slit width at 10.0 nm. After irradiation, 0.35 mL of the phenanthroline solution was added to the cuvette. The solution was then allowed to rest for 1 h to allow the ferrous ions to completely coordinate to the phenanthroline. The absorbance of the resulting solution was measured at 510 nm. A non-irradiated sample was also prepared and the absorbance at 510 nm measured.

Under light excitation the potassium ferrioxalate decomposes according to the following equations:

$$Fe(C_2O_4)_3^{3-} \xrightarrow{h\nu} Fe^{2+} + C_2O_4^{\bullet -} + 2C_2O_4^{2-}$$

$$Fe(C_2O_4)_3^{3-} + C_2O_4^{\bullet -} \xrightarrow{\Delta} Fe^{2+} + 2CO_2 + 3C_2O_4^{2-}$$

The quantity of ferrous ions formed during an irradiatin period is monitored by conversion to the colored tris-phenanthroline complex. The original ferric ions are not appreciably complexed by phenanthroline and the complex does not absorb at 510 nm. The mols of ferrous ions formed in the irradiated volume are given by eq 2.

$$\text{mol Fe}^{2+} = \frac{V \cdot \Delta A}{l \cdot \varepsilon} \qquad (2)$$

Where V is the total volume (0.00235 L) of the solution after addition of phenanthroline, $\Delta A$ is the difference in absorbance at 510 nm between the irradiated and non-irradiated solutions, l is the path length (1.000 cm), and $\varepsilon$ is the molar absorptivity of phenanthroline complex at 510 nm (11,100 L $mol^{-1}$ $cm^{-1}$).[16] The difference in absorbance at 510 nm between the irradiated and non-irradiated solutions was measured to be 0.328 (average of three experiments). The conversion was calculated using eq 2:

$$\text{mol Fe}^{2+} = \frac{0.00235\ \text{L} \cdot 0.328}{1.000\text{cm} \cdot 11{,}100\ \text{L mol}^{-1}\ \text{cm}^{-1}} = 6.94 \times 10^{-8}\ \text{mol}$$

The photon flux can be calculated using eq 3.

$$\text{photon flux} = \frac{\text{mol Fe}^{2+}}{\Phi_{Fe} \cdot t \cdot f_{Fe}} \qquad (3)$$

Where mol $Fe^{2+}$ is the mols of $Fe^{2+}$ formed during irradiation (6.94 $\times 10^{-8}$ mol), $\Phi_{Fe}$ is the

quantum yield for the ferrioxalate actinometer (1.01 for a 0.15 M solution at λ = 436 nm),[2] t is the time (90.0 s), and $f_{Fe}$ is the fraction of light absorbed of the ferrioxalate solution at λ = 436 nm.

The fraction of light absorbed ($f_{Fe}$) by this solution was calculated using eq 4, where A is the measured absorbance at 436 nm.

$$F_{fe} = 1\text{-}10^{-A} \qquad (4)$$

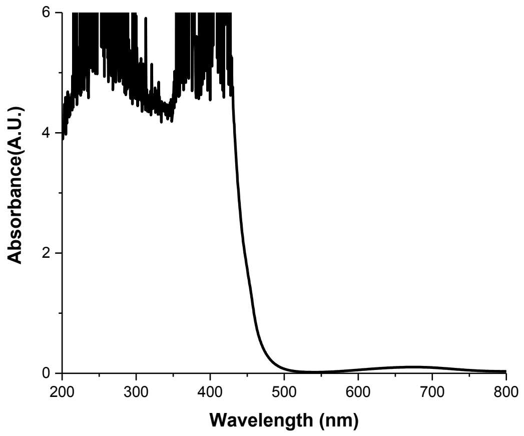


**Figure S3.** Absorbance of the ferrioxalate actinometer solution.

The absorbance of the above ferrioxalate solution at 436 nm was measured to be 3.389 (average of three experiments). The light absorbed ($f_{Fe}$) was calculated using eq 4:

$$f_{Fe} = 1\text{-}10^{-3.389} = 0.99959$$

The photon flux was calculated using eq 3:

$$\text{photon flux} = \frac{6.94\times10^{-8}\ \text{mol}}{1.01 \cdot 90.0\ \text{s} \cdot 0.99959} = 7.63\times10^{-10}\ \text{einstein s}^{-1}$$

## C) Determination of quantum yield:

A clear vial was charged with **32** (21.7 mg, 1.0 mmol, 1.0 equiv), azidoiodinane **1** (57.8 mg, 2.0 mmol, 2.0 equiv), $Ru(bpy)_3Cl_2$ (0.001 mmol, 0.1 mol%), and 0.5 mL HFIP (2.0 M). The resulting mixture was purged with Ar and the cuvette was then capped with a PTFE stopper. The reaction mixture was irradiated (λ = 436 nm, slit width= 10.0 nm) for 21600 s (6 h). After irradiation, the solvent of the reaction mixture was removed under reduced pressure. The yield of was determined by $^1H$ NMR based on a $Cl_2CHCHCl_2$ standard to be 30%.
The quantum yield was calculated using eq 1:

$$\Phi = \frac{1\ \text{mmol}\cdot 30\%}{7.63\times10^{-10}\ \text{einstein s}^{-1} \cdot 21600\ \text{s} \cdot 1.00} = 18$$

The quantum yield was calculated to be Φ(30%) = 18, indicating that 18 equivalents of

product are formed for every photon absorbed by the photocatalyst.

### 10. Light/dark experiment of C-H azidation of 32

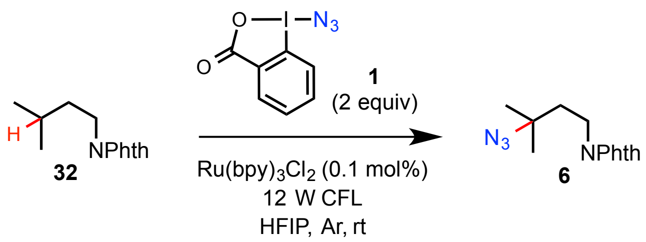


**Scheme S17**

Six vials were equipped with a stir bar and charged with **32** (21.7 mg, 0.10 mmol, 1.0 equiv) and 0.05 mL of the 0.002 M $Ru(bpy)_3Cl_2$ solvent. Azidoiodinane **1** (57.8 mg, 0.2 mmol, 2.0 equiv) was then added and the reaction was stirred under Ar atmosphere. The reactions was alternatively irradiated with a 12 W CFL bulb and kept in the dark in 1 h intervals. After each interval, one vial was take out, the solvent was removed under reduced pressure, and the yield was determined by $^1$H NMR based on a $Cl_2CHCHCl_2$ as an internal standard.

| Vial | Time (h)/condition | | | | | | Yield (%)[a] |
|---|---|---|---|---|---|---|---|
| 1 | 0-1/hv | | | | | | 28 |
| 2 | 0-1/hv | 1-2/dark | | | | | 28 |
| 3 | 0-1/hv | 1-2/dark | 2-3/hv | | | | 60 |
| 4 | 0-1/hv | 1-2/dark | 2-3/hv | 3-4/dark | | | 60 |
| 5 | 0-1/hv | 1-2/dark | 2-3/hv | 3-4/dark | 4-5/hv | | 70 |
| 6 | 0-1/hv | 1-2/dark | 2-3/hv | 3-4/dark | 4-5/hv | 5-6/dark | 70 |

**Table S1. Yields of Light/Dark experiment.** a) NMR yield, average of three experiments.

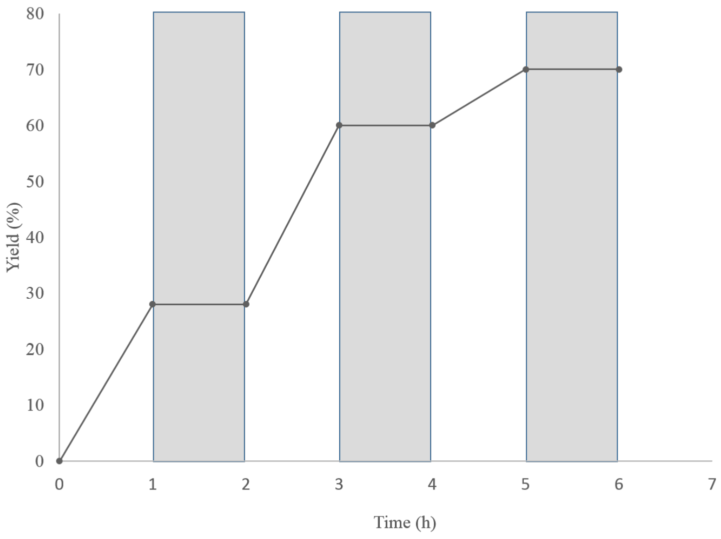


**Figure S4.** Light/dark experiment of C-H azidation of **32**

## 11. References


1. V. V. Zhdankin, A. P. Krasutsky, C. J. Kuehl, A. J. Simonsen, J. K. Woodward, B. Mismash, J. T. Bolz, *J. Am. Chem. Soc.* **1996**, *118*, 5192.
2. S. Akai, T. Okuno, M. Egi, T. Takada, H. Tohma, Y. Kita, *Heterocycles* **1996**, *42*, 47.
3. A. Sharma, J. F. Hartwig, *Nature* **2015**, *517*, 600.
4. V. Matoušek, E. Pietrasiak, R. Schwenk, A. Togni, *J. Org. Chem.* **2013**, *78*, 6763.
5. A. G. Griesbeck, H. Mauder, I. Müller, *Chem. Ber.* **1992**, *125*, 2467.
6. S. Guo, X. F. Zhang, P. P. Tang, *Angew. Chem., Int. Ed.* **2015**, *54*, 4065.
7. M. A. A. Rani, N. Borduas, V. Colquhoun, R. Hanley, H. Johnson, S. Larger, P. D. Lickiss, V. Llopis-Mestre, S. Luu, M. Mogstad, P. Oczipka, J. R. Sherwood, T. Welton, J. Y. Xing, *Green Chem*. **2014**, *16*, 1282.
8. M. Ochiai, A. Yoshimura, M. M. Hoque, T. Okubo, M. Saito, K. Miyamoto, *Org. Lett.* **2011,** *13*, 5568.
9. D. Donati, C. Morelli, M. Taddei, *Tetrahedron Lett.* **2005**, *46*, 2817.
10. N. Iranpoor, H. Firouzabadi, D. Khalili, S. Motevalli, *J. Org. Chem.* **2008**, *73*, 4882.
11. X. Huang, T. M. Bergsten, J. T. Groves, *J. Am. Chem. Soc.* **2015**, *137*, 5300.
12. J. D. Winkler, A. Isaacs, L. Holderbaum, V. Tatardand, N. Dahmane, *Org. Lett.* **2009**, *11*, 2824.
13. V. A. Schmidt, R. K. Quinn, A. T. Brusoe, E. J. Alexanian, *J. Am. Chem. Soc.* **2014**, *136*, 14389.
14. S. Antonello, F. Maran, *J. Am. Chem. Soc.* **1998**, *120*, 5713.
15. M. A. Cismesia and T. P. Yoon, *Chem. Sci.,* 2015, **6,** 5426
16. M. Montalti, A. Credi, L. Prodi, M. T. Gandolfi, Chemical Actinometry, *Handbook of Photochemistry*, 3rd Ed; Taylor & Francis Group, LLC. Boca Raton, FL, 2006, 601–616.

## 12. $^{1}$H-NMR and $^{13}$C-NMR spectra

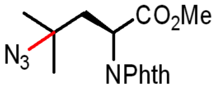


**3**

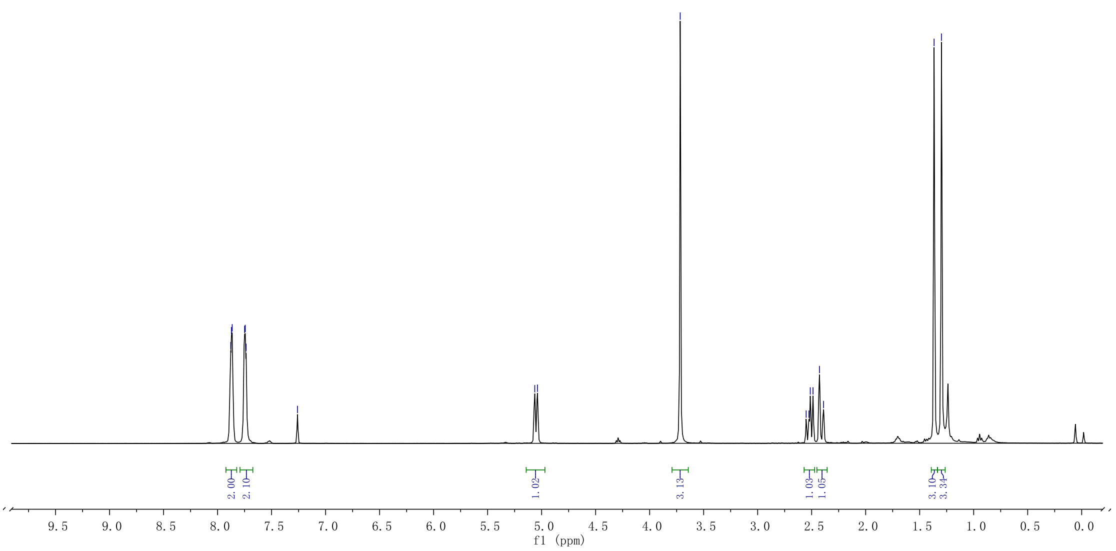


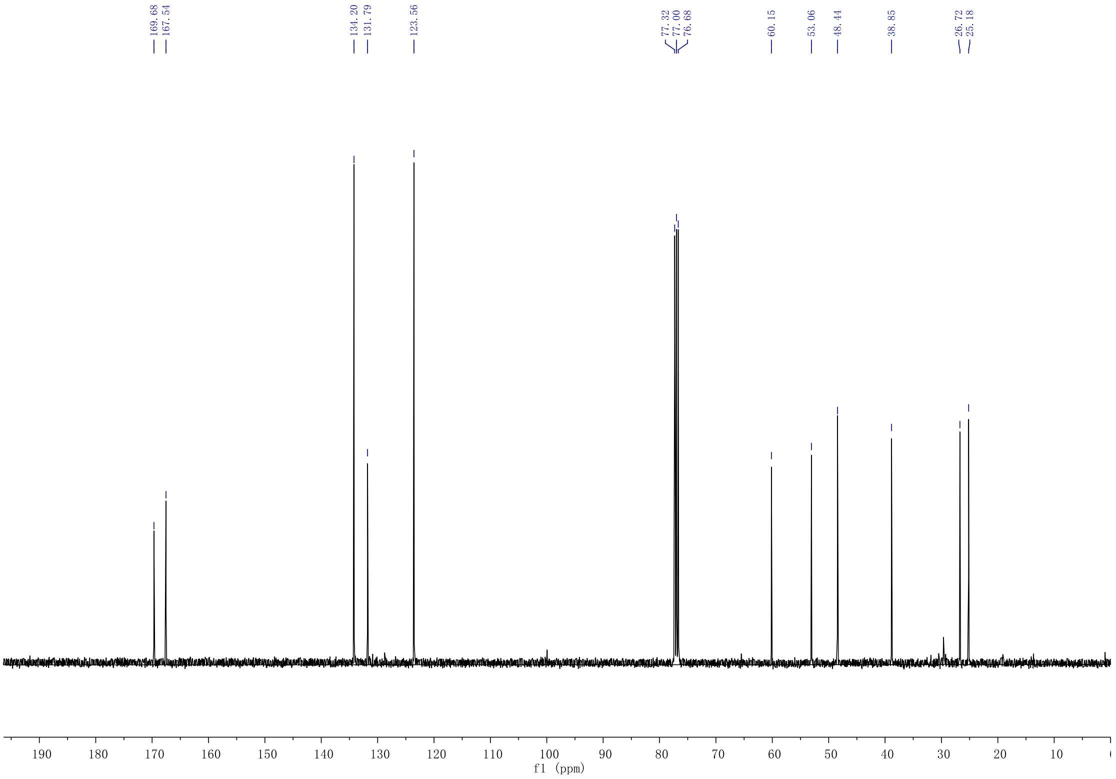

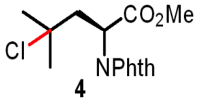
CO2Me
Cl
NPhth

**4**

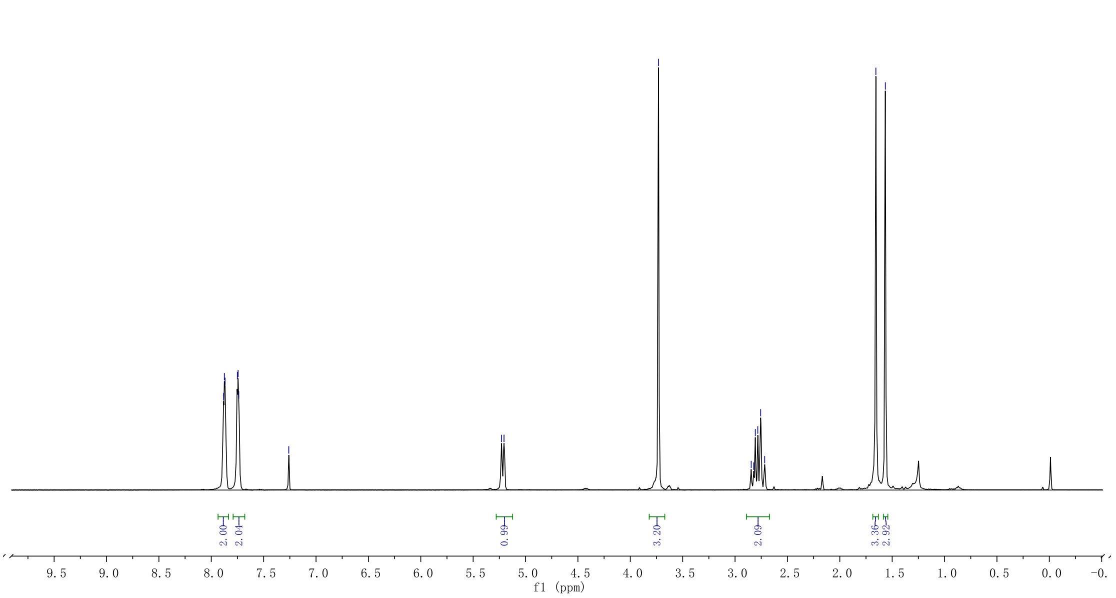
7.88
7.88
7.87
7.75
7.74
7.74
7.26
5.23
5.21
3.73
2.85
2.82
2.81
2.78
2.76
2.72
1.66
1.57
2.00
2.04
0.99
3.20
2.09
3.36
2.92
f1 (ppm)

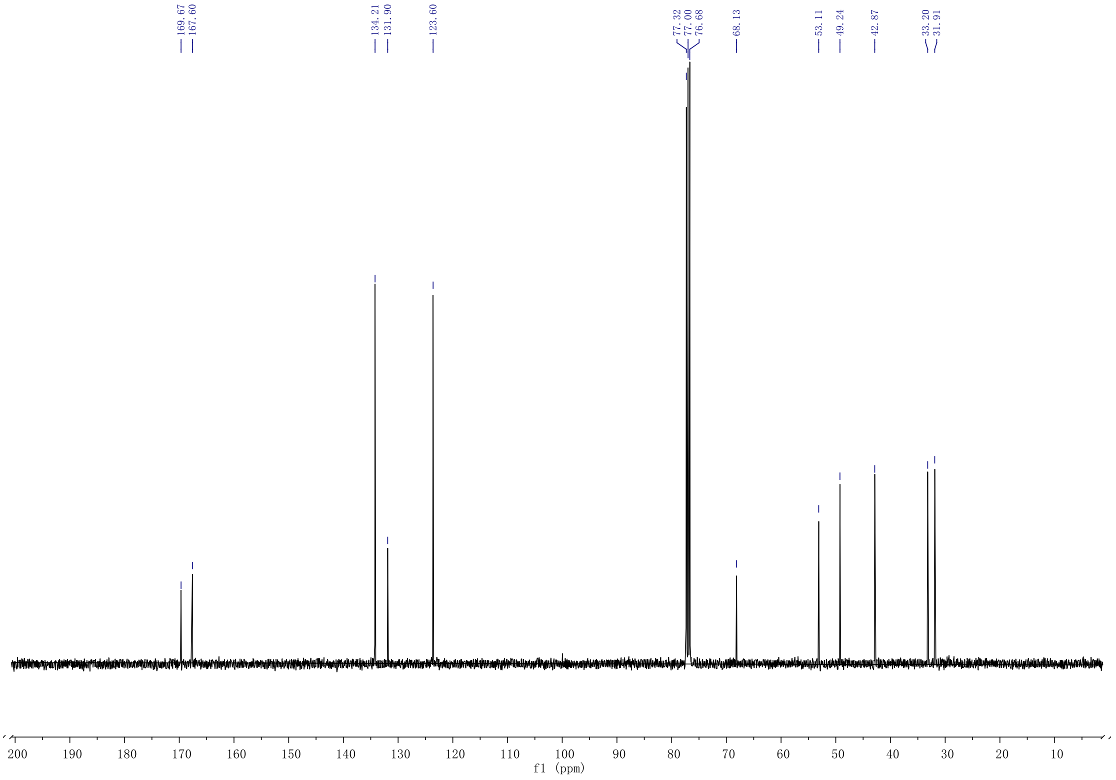
169.67
167.60
134.21
131.90
123.60
77.32
77.00
76.68
68.13
53.11
49.24
42.87
33.20
31.91
f1 (ppm)

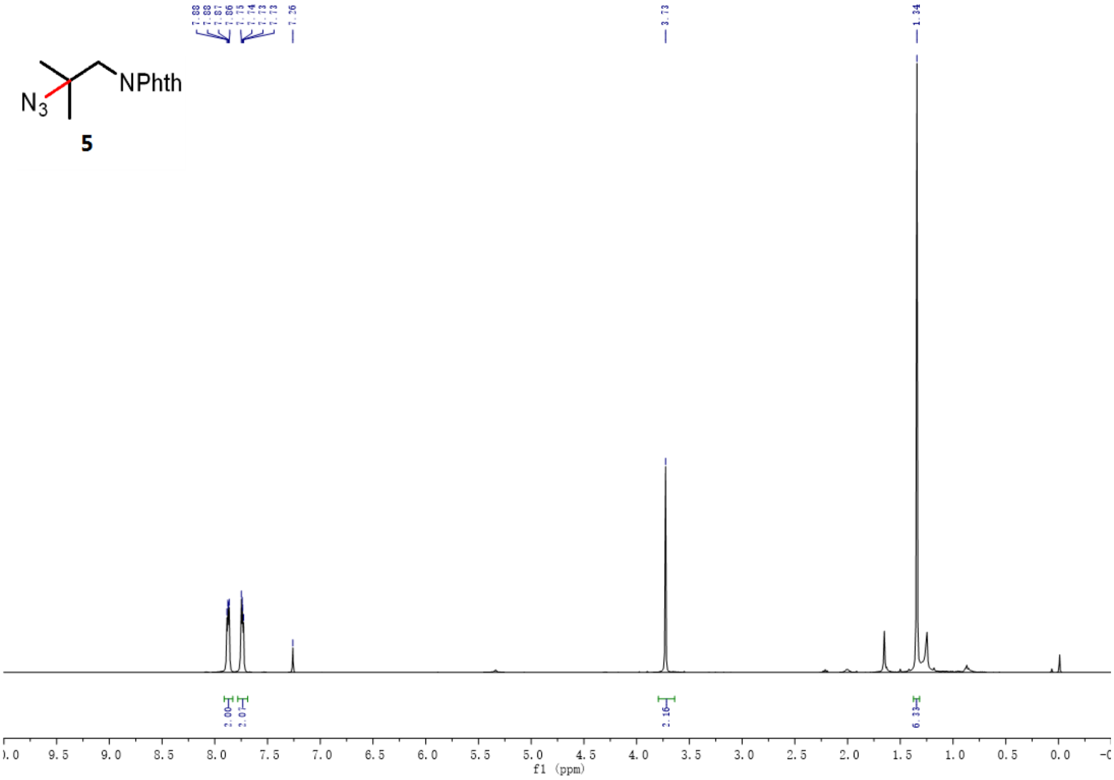
NPhth
$N_3$
5
7.88
7.88
7.87
7.86
7.75
7.74
7.73
7.73
7.26
3.73
1.34
2.00
2.07
2.16
6.33
f1 (ppm)

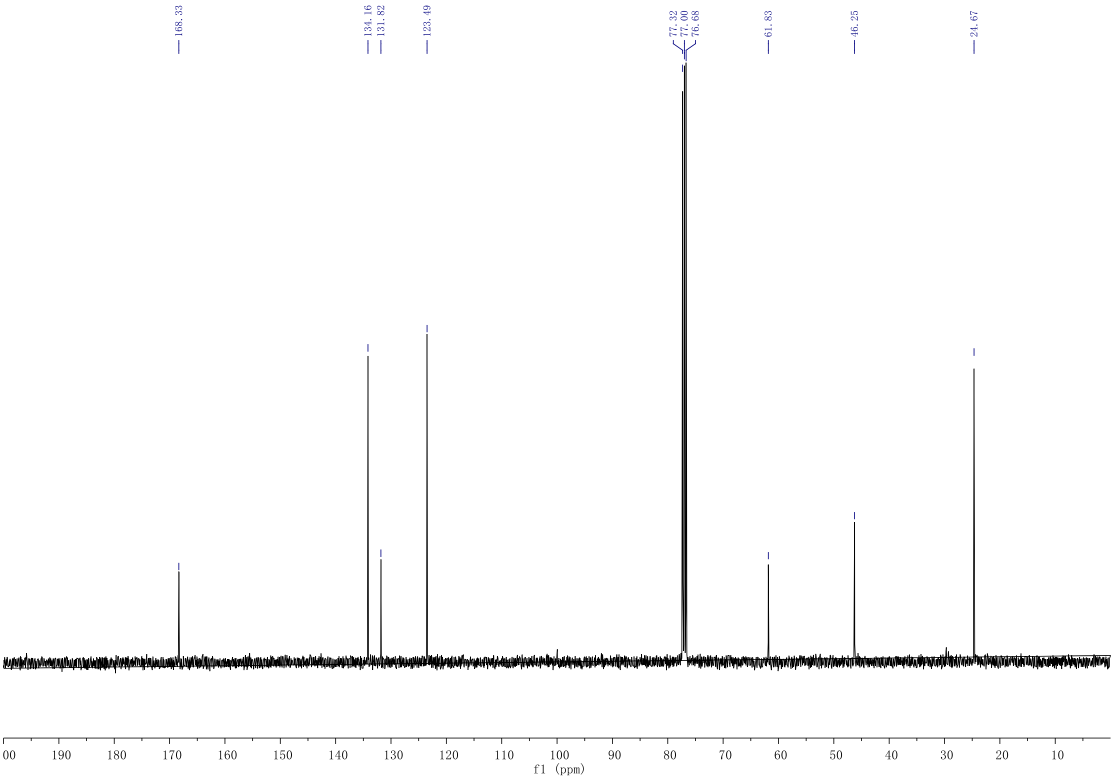
168.33
134.16
131.82
123.49
77.32
77.00
76.68
61.83
46.25
24.67
f1 (ppm)

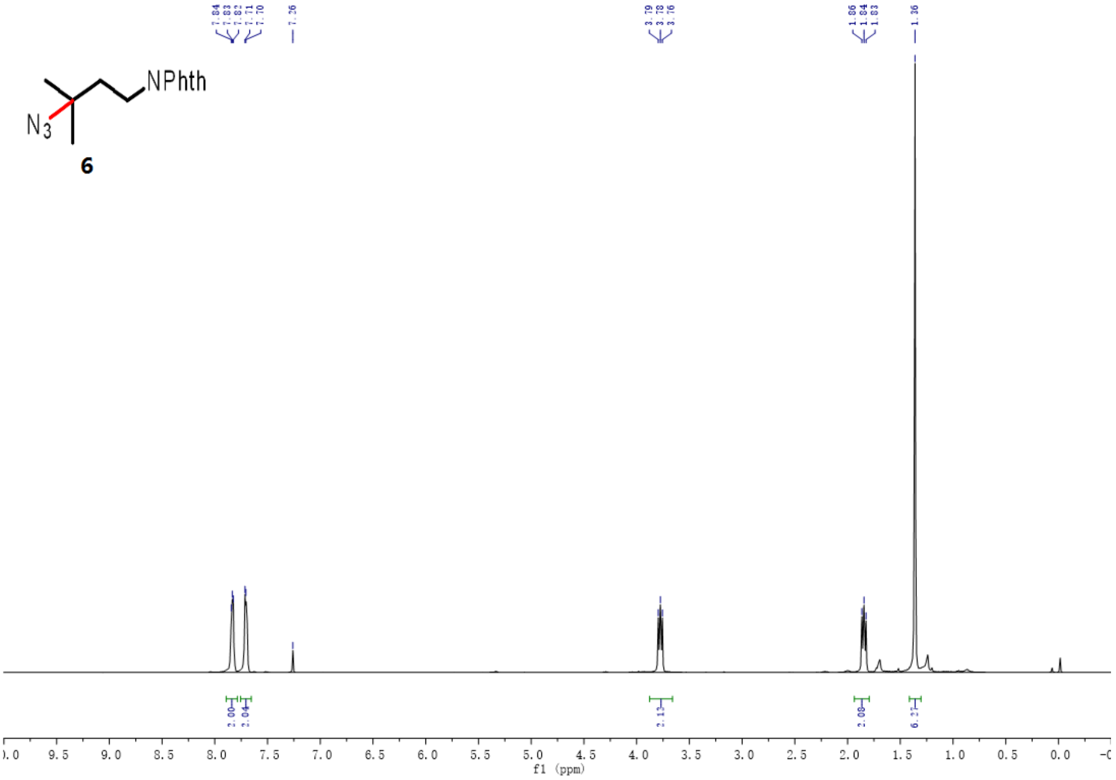

6

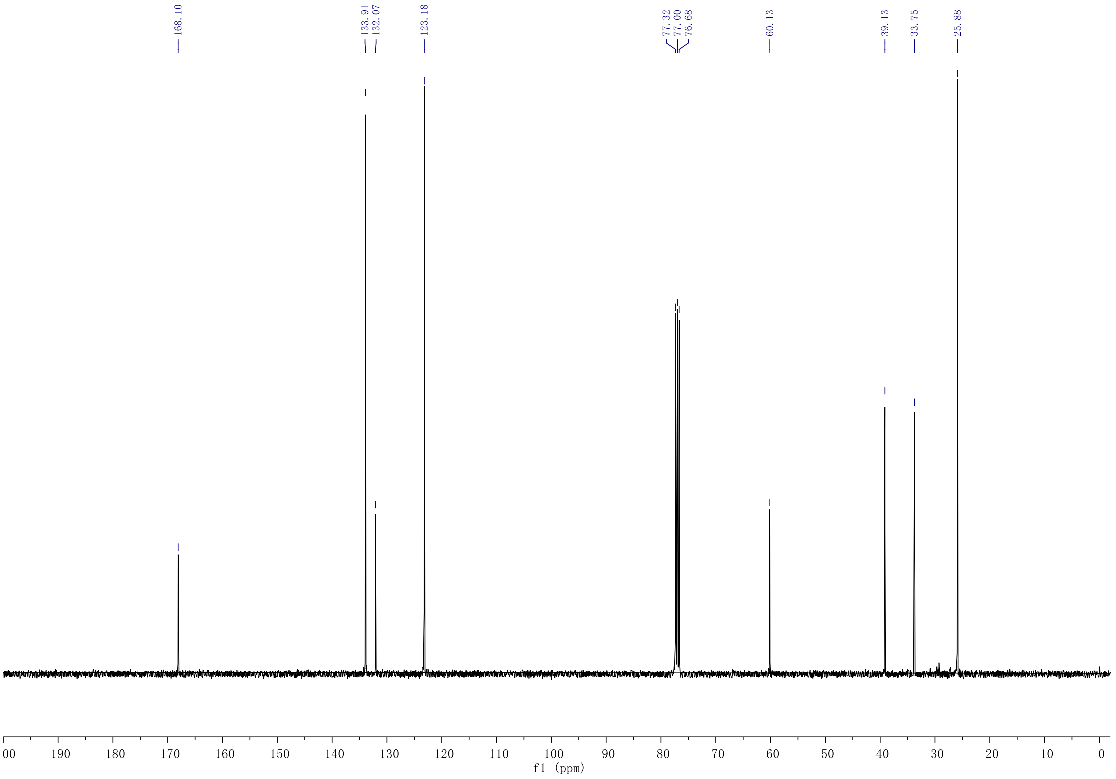

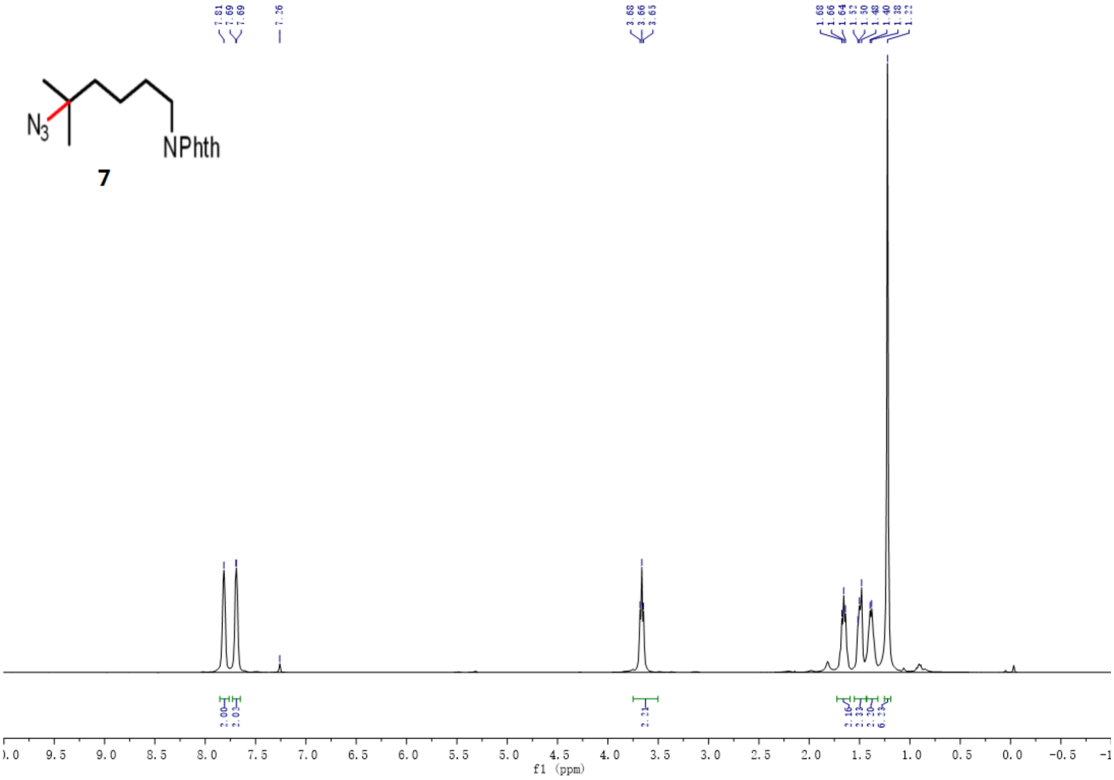
7.81
7.69
7.69
7.26
3.68
3.66
3.65
1.68
1.66
1.64
1.52
1.50
1.40
1.38
1.22
N3
NPhth
7
f1 (ppm)

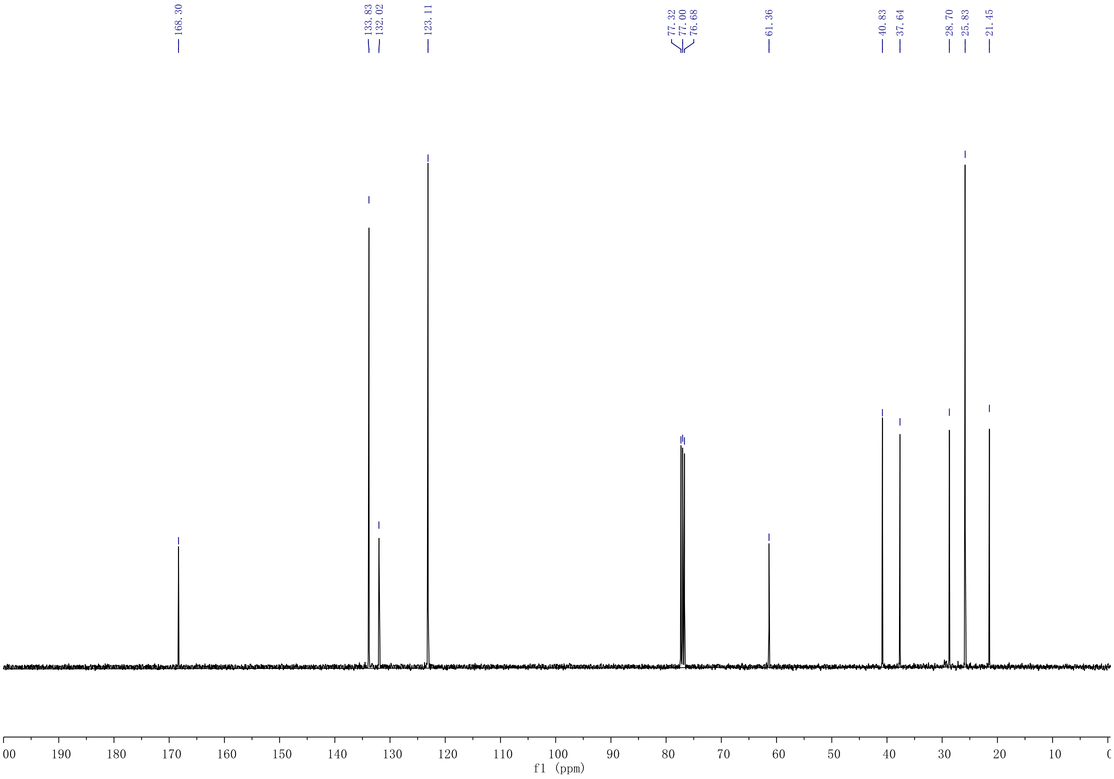
168.30
133.83
132.02
123.11
77.32
77.00
76.68
61.36
40.83
37.64
28.70
25.83
21.45
f1 (ppm)

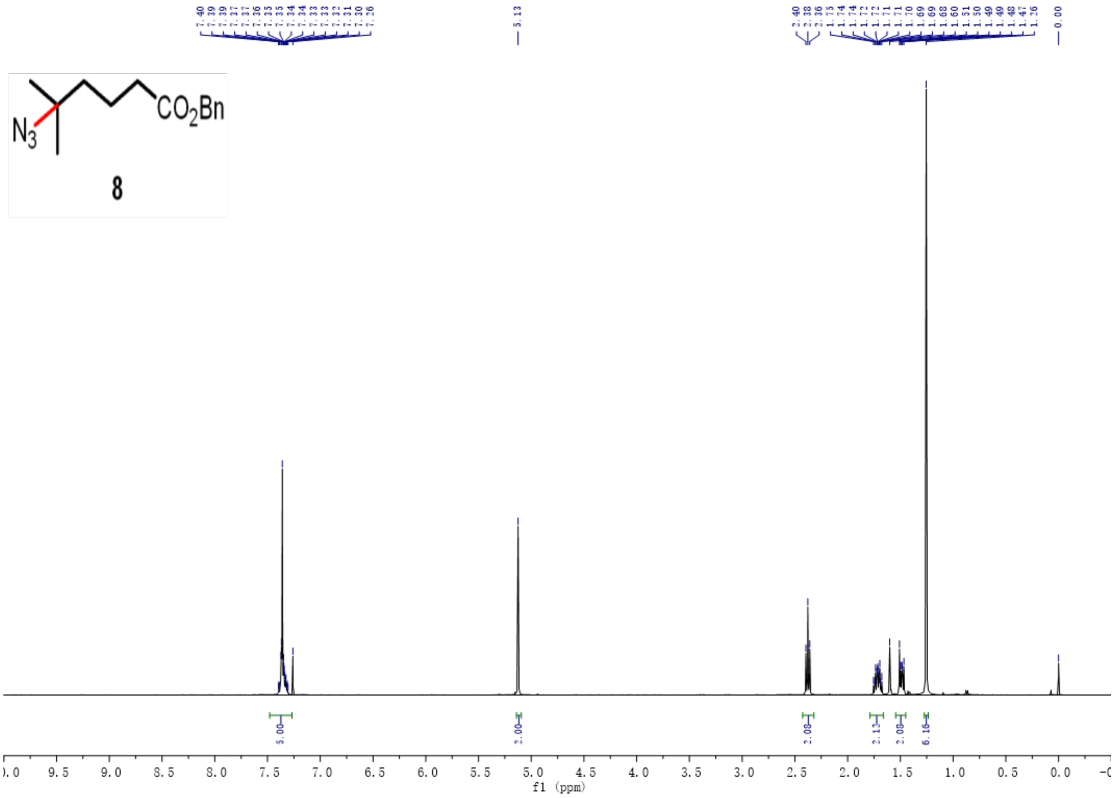

N3
CO2Bn
8
5.13
0.00
5.00
2.00
2.08
2.13
2.08
6.16
f1 (ppm)


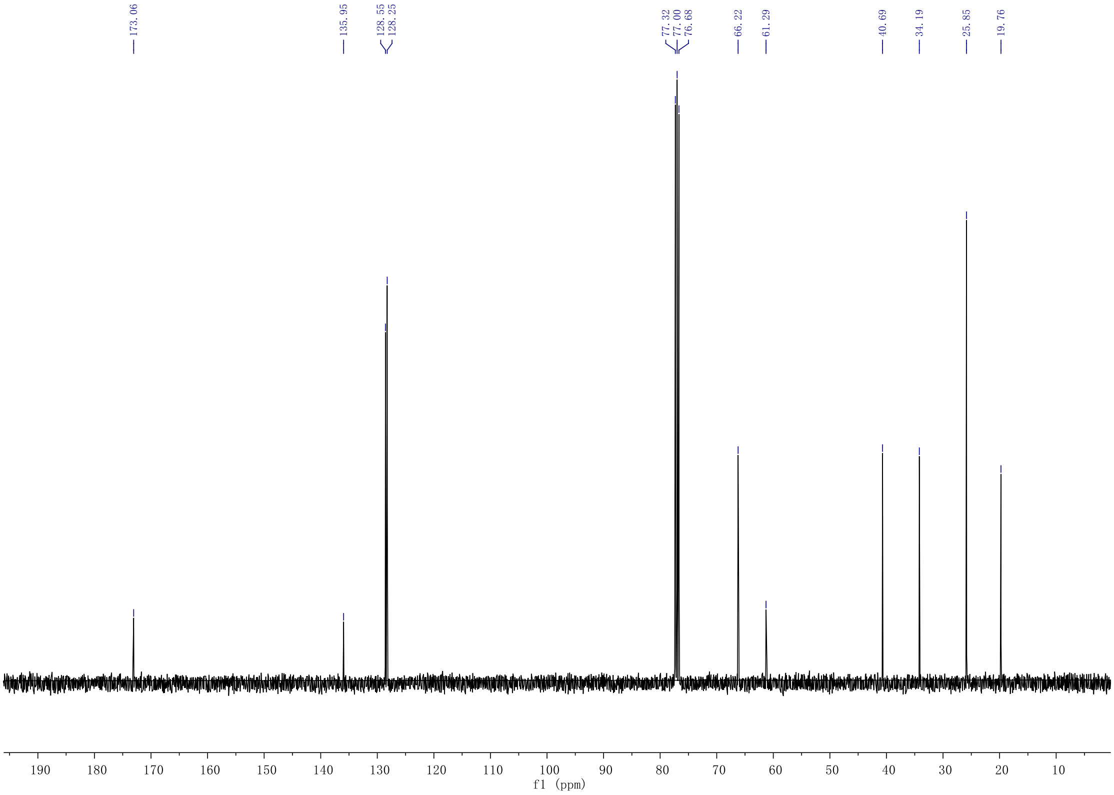

173.06
135.95
128.55
128.25
77.32
77.00
76.68
66.22
61.29
40.69
34.19
25.85
19.76
f1 (ppm)

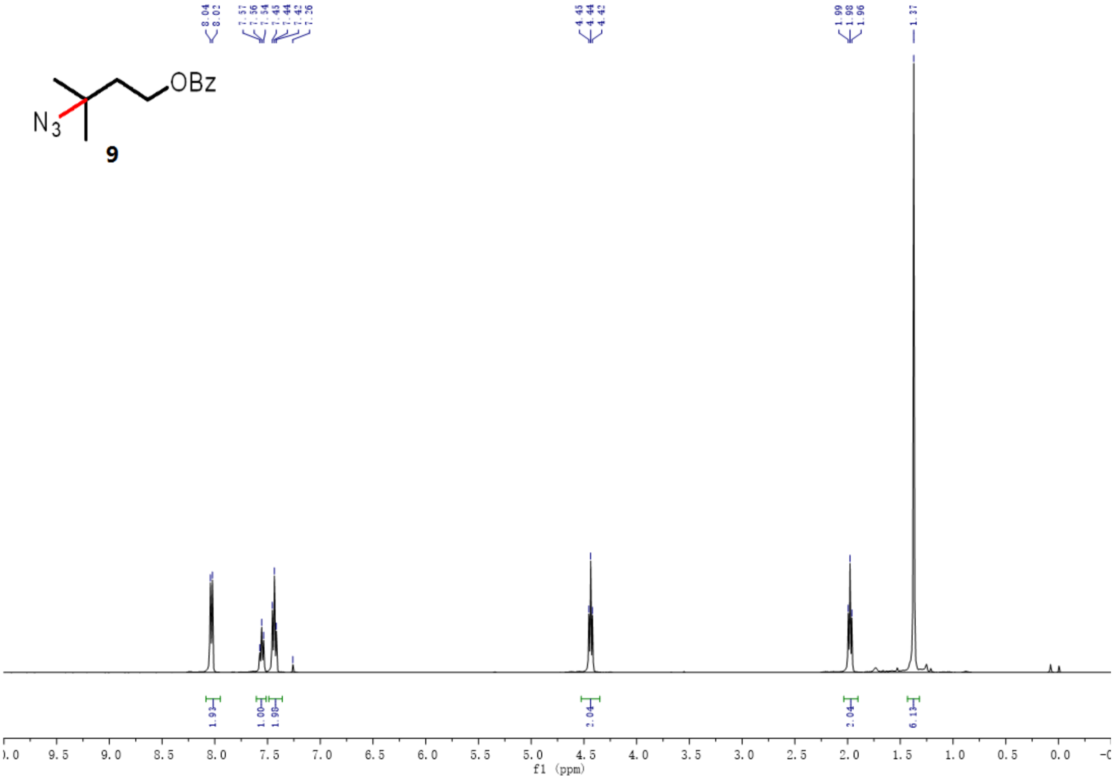

OBz
N3
9
8.04
8.02
7.57
7.56
7.54
7.45
7.44
7.42
7.26
4.45
4.44
4.42
1.99
1.98
1.96
1.37
1.93
1.00
1.98
2.04
2.04
6.13
f1 (ppm)


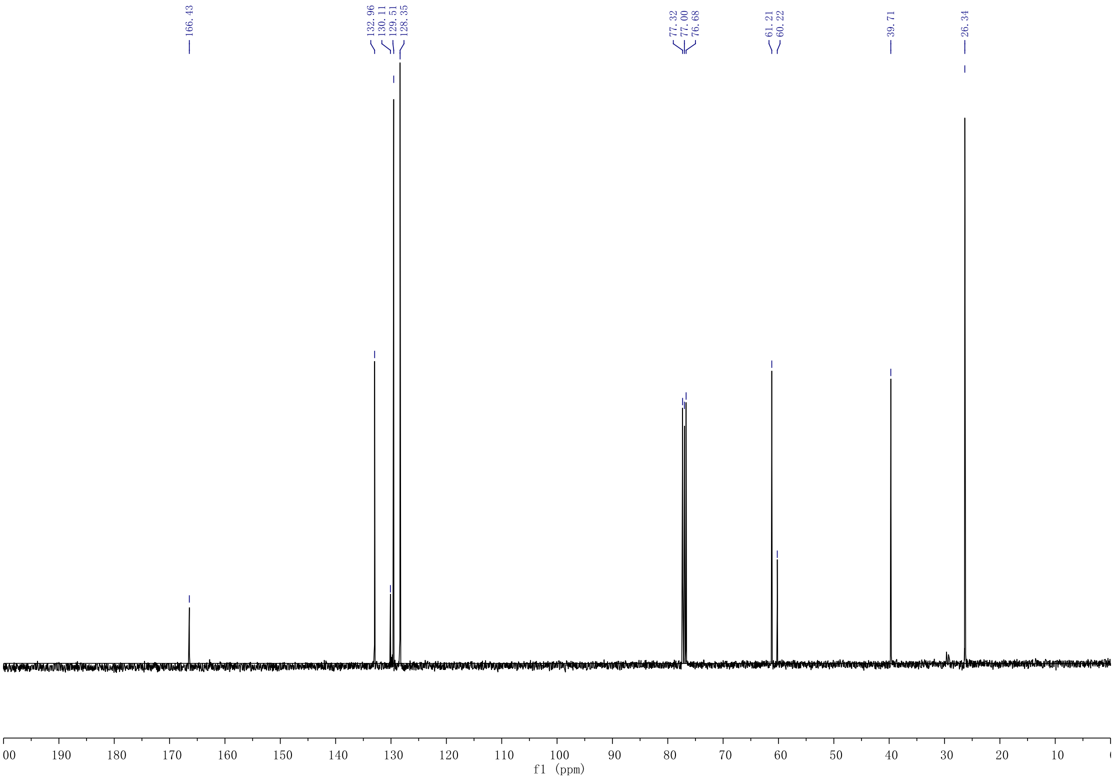

166.43
132.96
130.11
129.51
128.35
77.32
77.00
76.68
61.21
60.22
39.71
26.34
f1 (ppm)

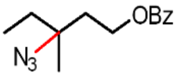
OBz
N3

**10**

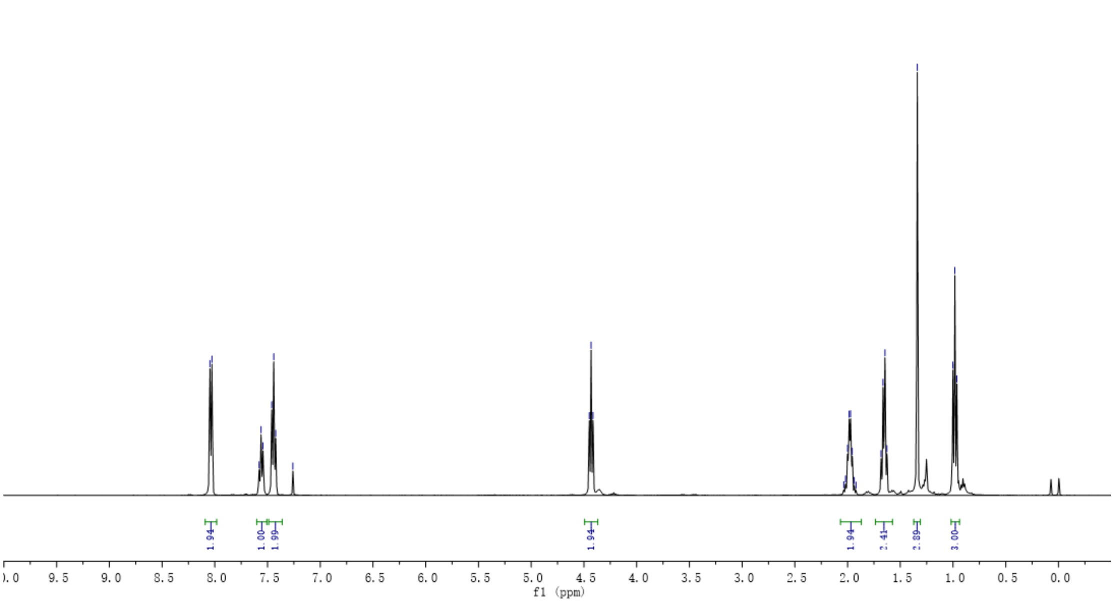
8.05
8.03
7.58
7.56
7.54
7.46
7.44
7.42
7.26
4.45
4.43
4.41
2.04
2.02
2.00
1.99
1.97
1.96
1.94
1.92
1.66
1.65
1.00
0.98
0.96
1.94
1.00
1.99
1.94
1.94
3.00
f1 (ppm)

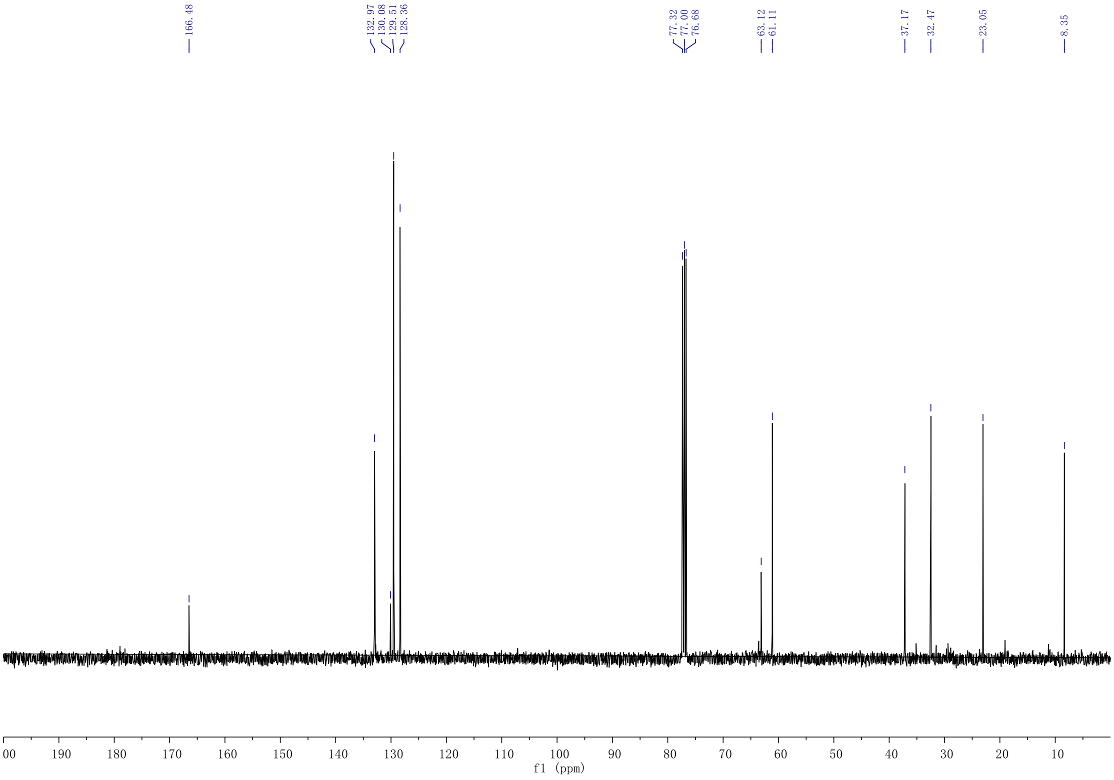
166.48
132.97
130.08
129.51
128.36
77.32
77.00
76.68
63.12
61.11
37.17
32.47
23.05
8.35
f1 (ppm)

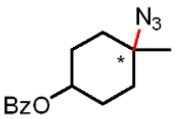

N3
BzO


**11'**

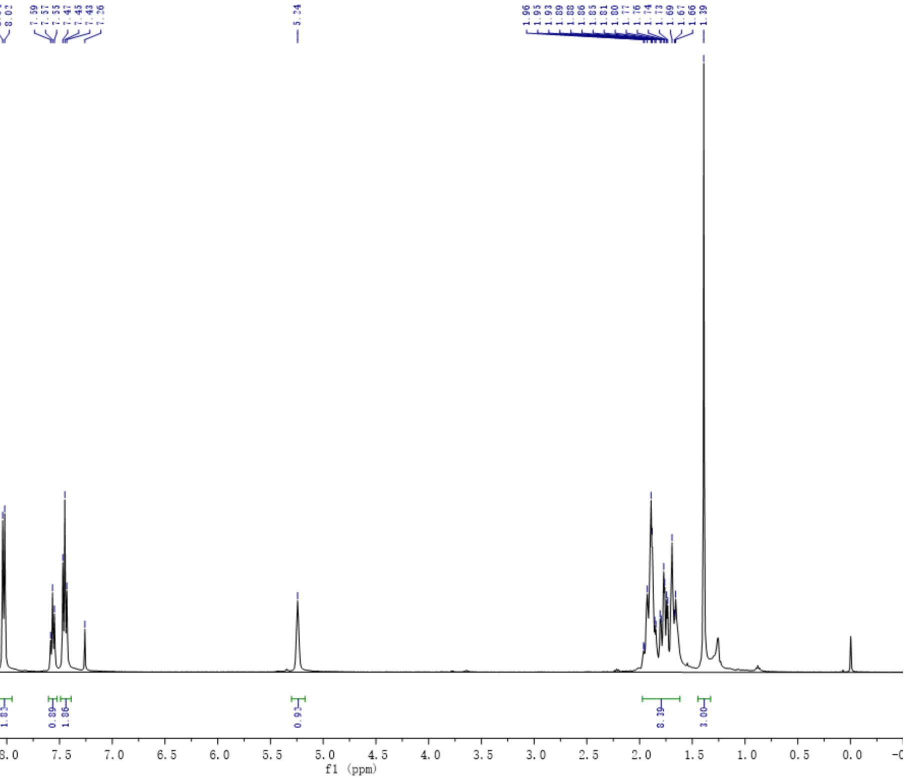

1.83
0.89
1.86
0.93
8.39
3.00
f1 (ppm)


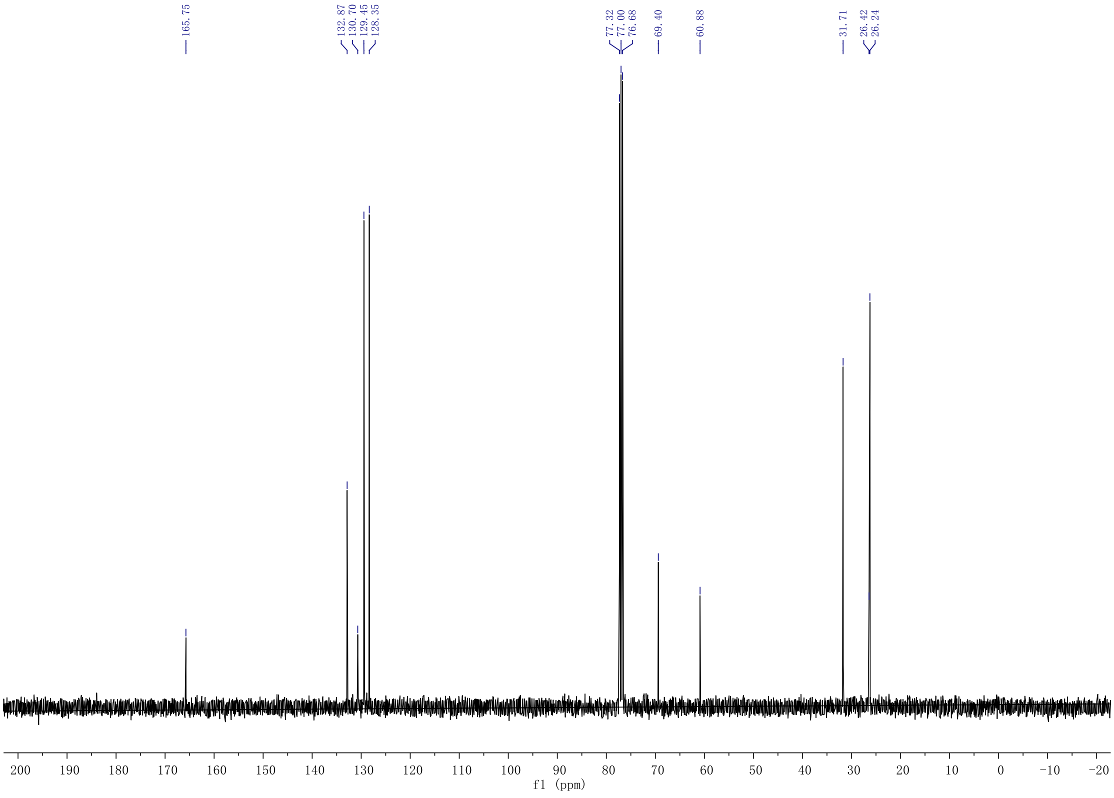

165.75
132.87
130.70
129.45
128.35
77.32
77.00
76.68
69.40
60.88
31.71
26.42
26.24
f1 (ppm)

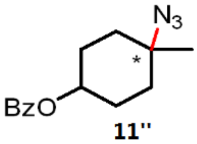
N3
BzO
*

**11''**

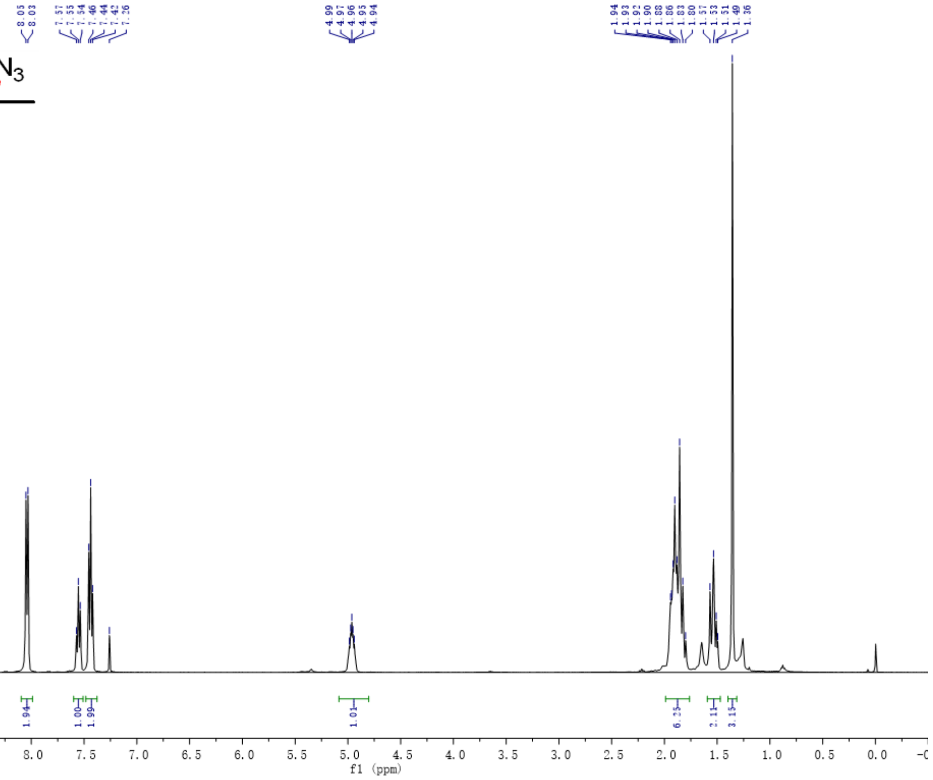
8.05
8.03
7.57
7.55
7.54
7.46
7.44
7.42
7.26
4.99
4.97
4.96
4.95
4.94
1.94
1.93
1.92
1.90
1.88
1.86
1.83
1.80
1.57
1.53
1.51
1.49
1.36
1.94
1.00
1.99
1.01
6.25
2.11
3.15
f1 (ppm)

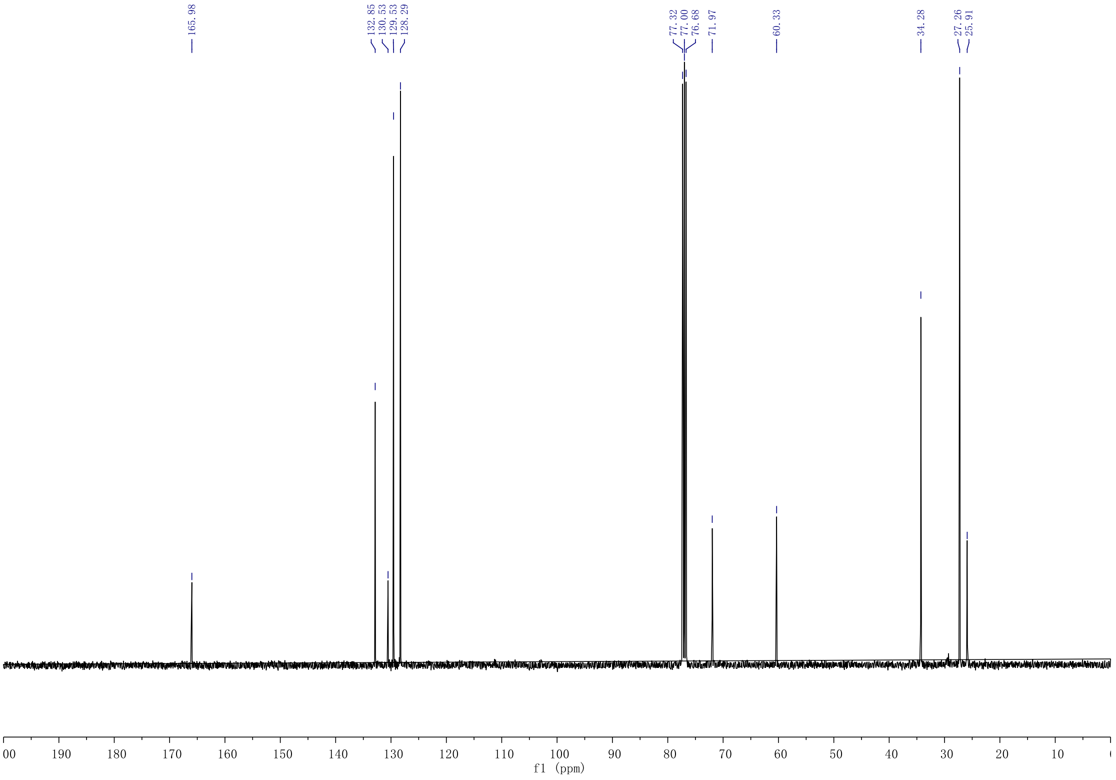
165.98
132.85
130.53
129.53
128.29
77.32
77.00
76.68
71.97
60.33
34.28
27.26
25.91
f1 (ppm)

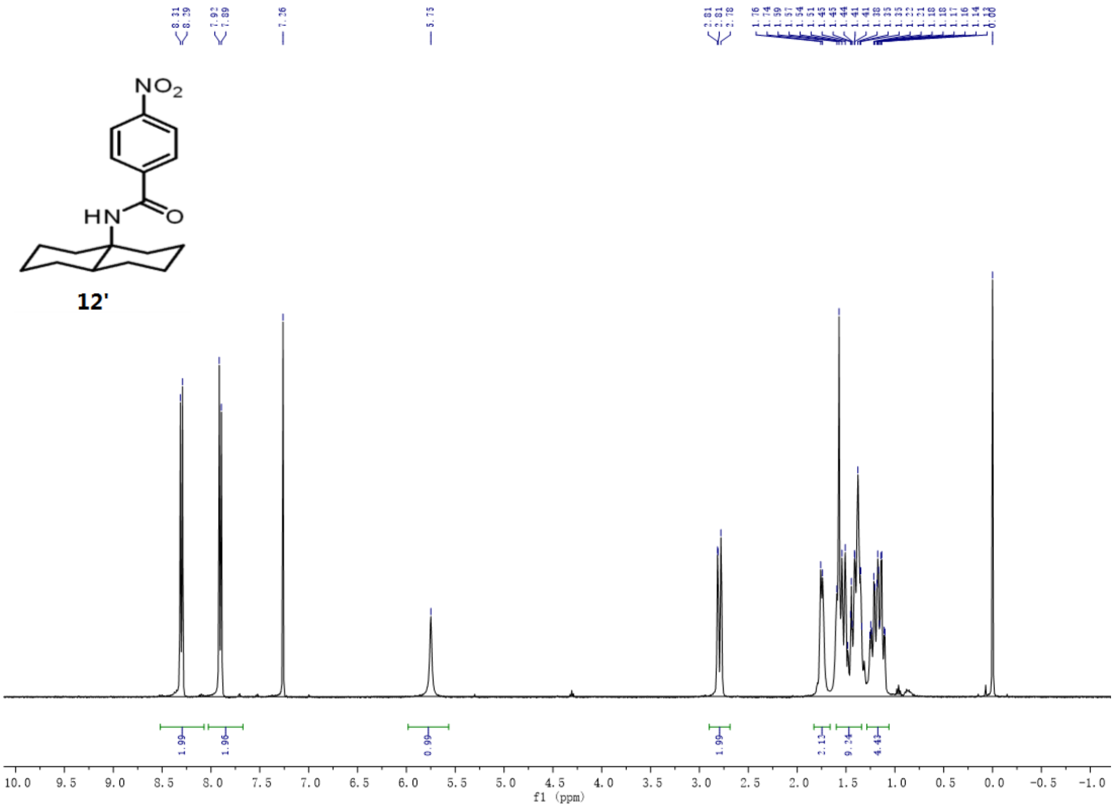
8.31
8.29
7.92
7.89
7.26
5.75
1.99
1.96
0.99
1.99
2.13
9.24
4.43
f1 (ppm)

12'

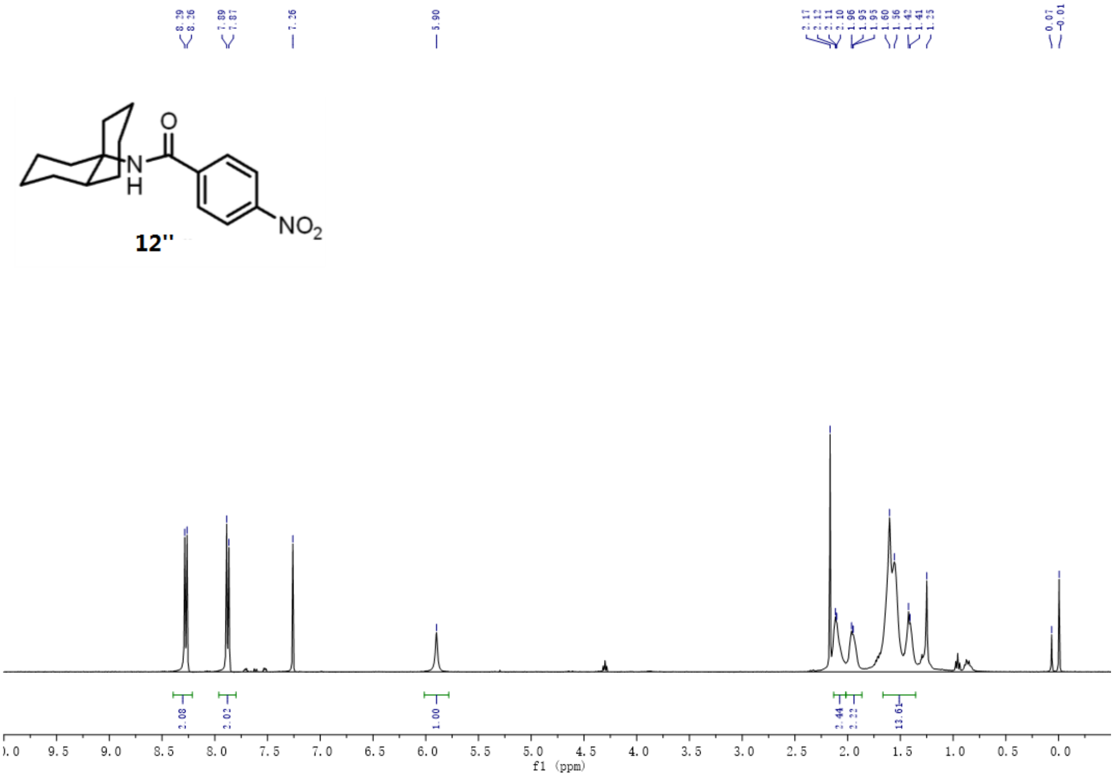
8.29
8.26
7.89
7.87
7.26
5.90
0.07
-0.01
2.08
2.02
1.00
2.44
2.22
13.61
f1 (ppm)

12''

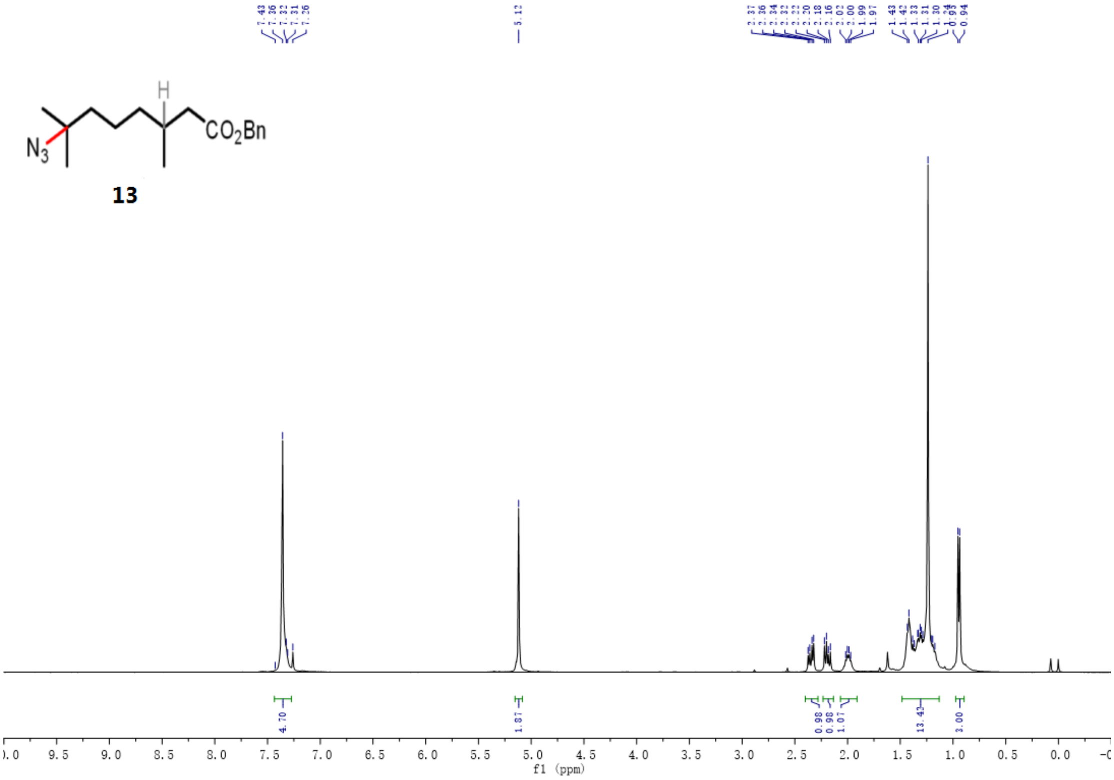

13

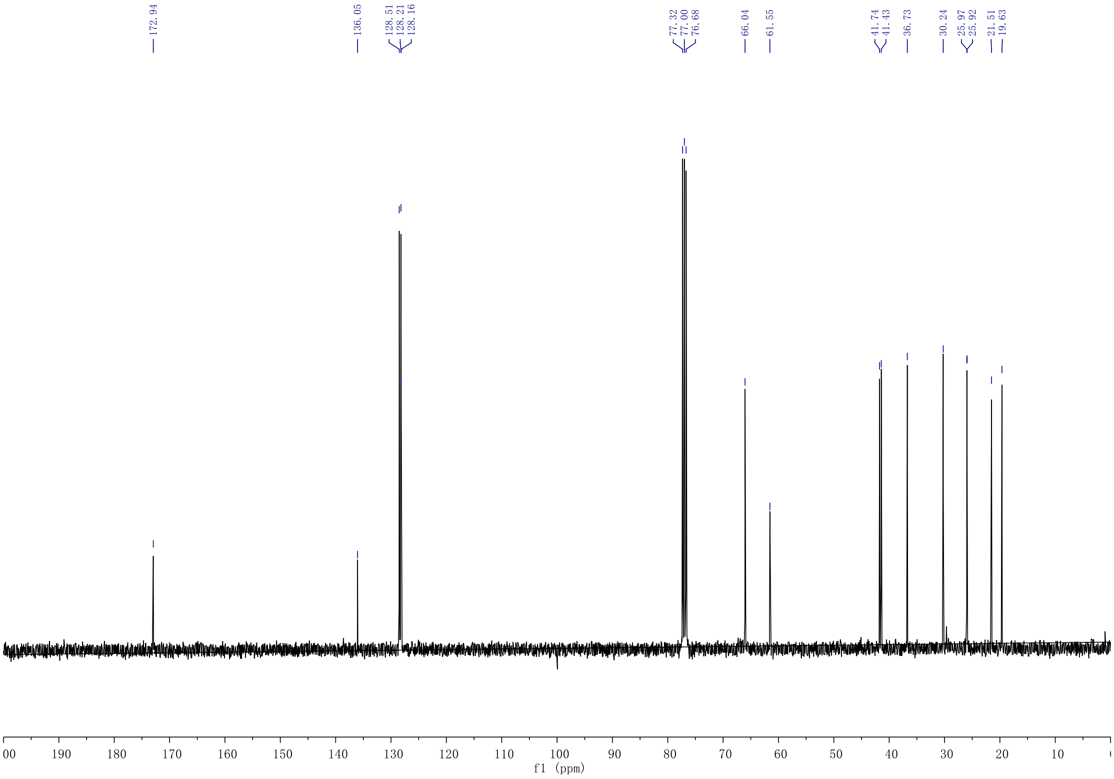

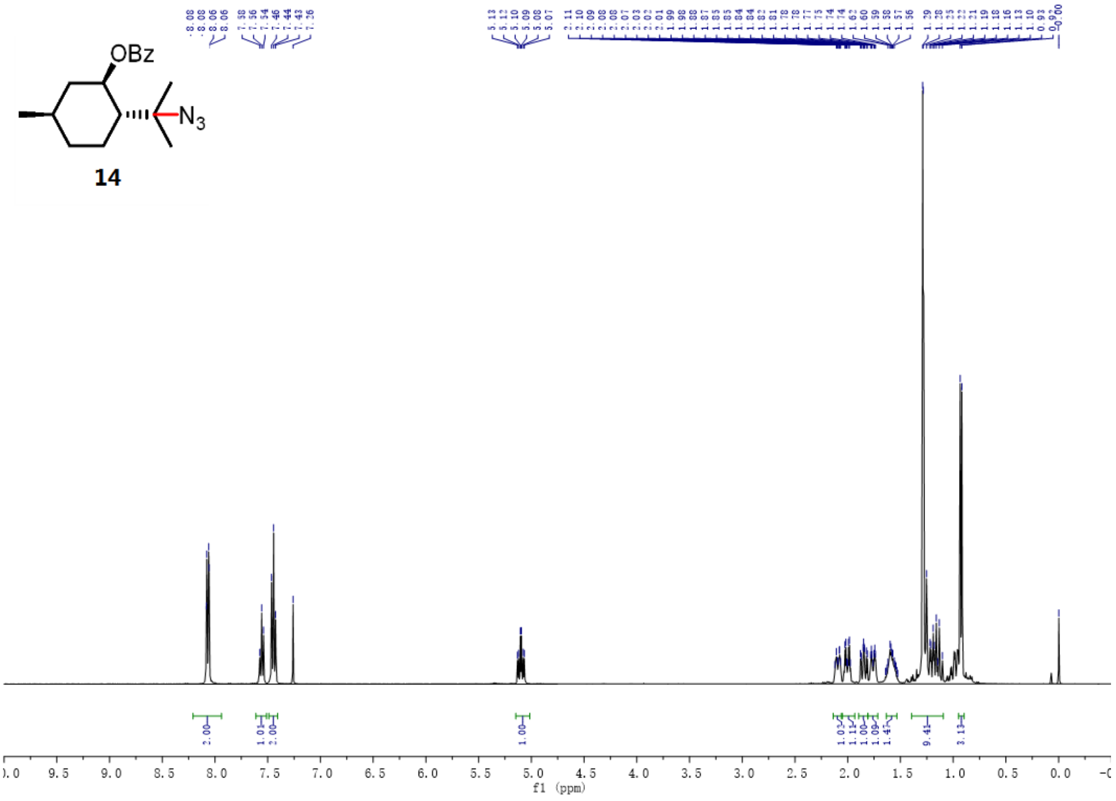

**14**

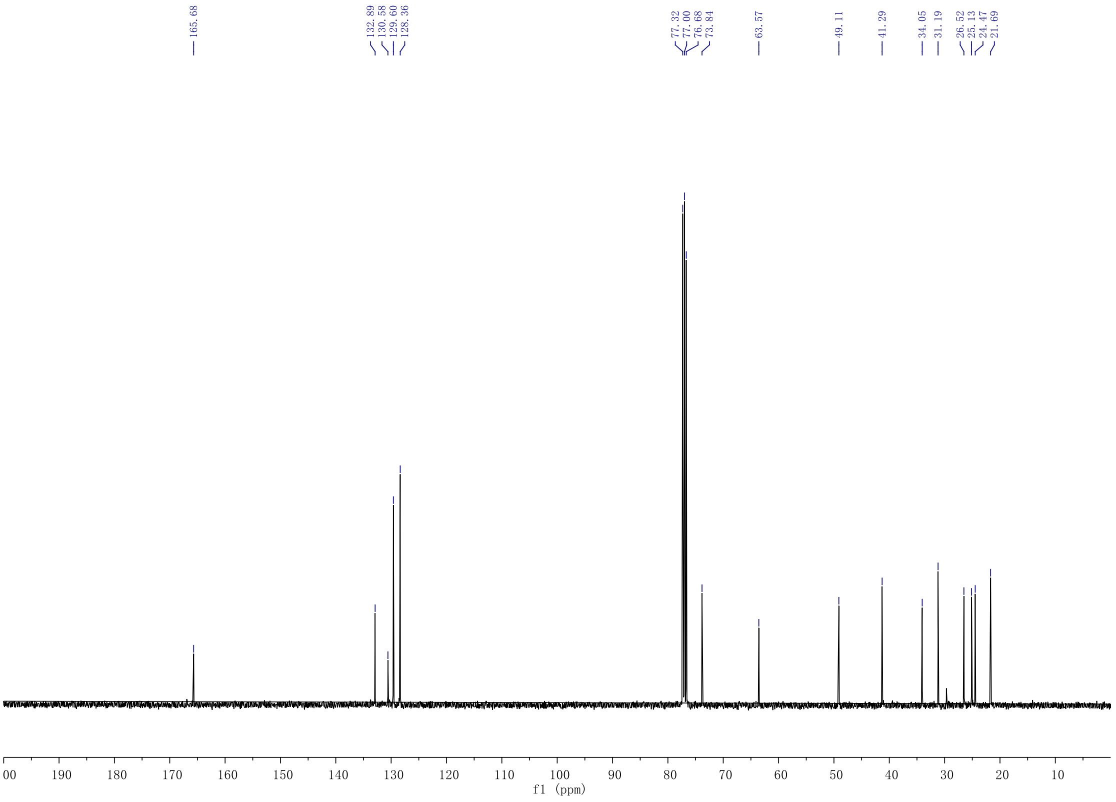

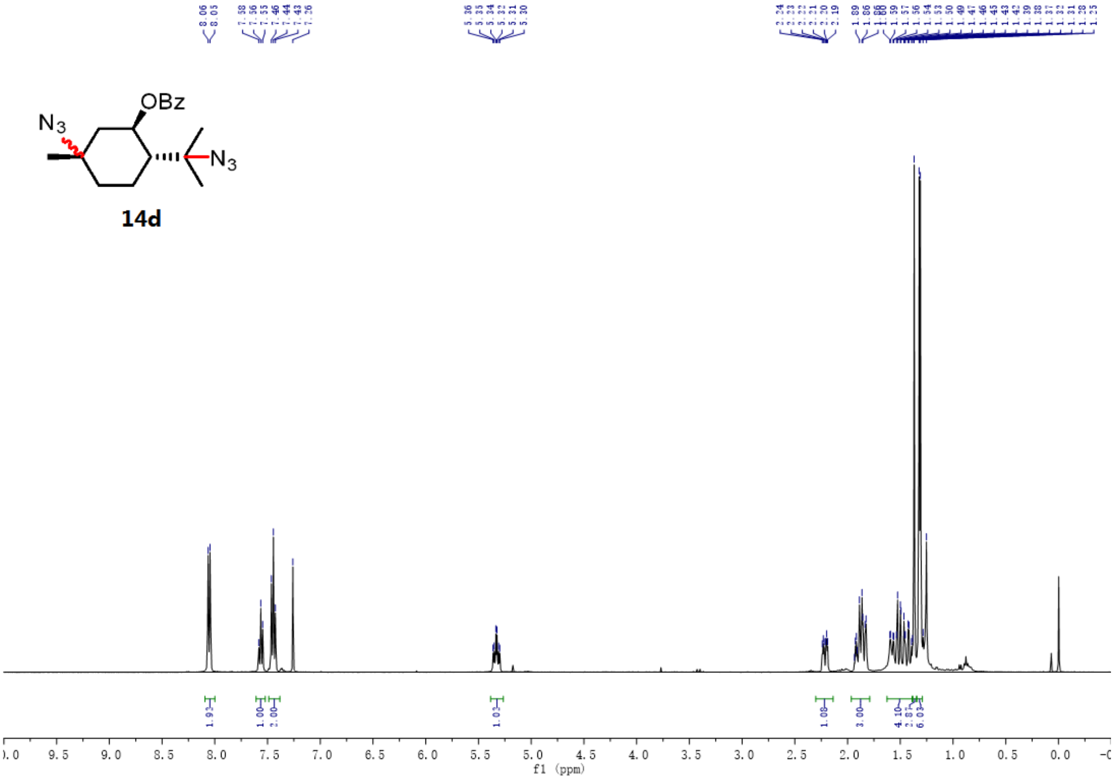
OBz
N3
N3
14d
8.06
8.05
7.58
7.56
7.55
7.46
7.44
7.43
7.26
5.36
5.35
5.34
5.32
5.31
5.30
1.93
1.00
2.00
1.02
1.08
3.00
4.10
6.03
f1 (ppm)

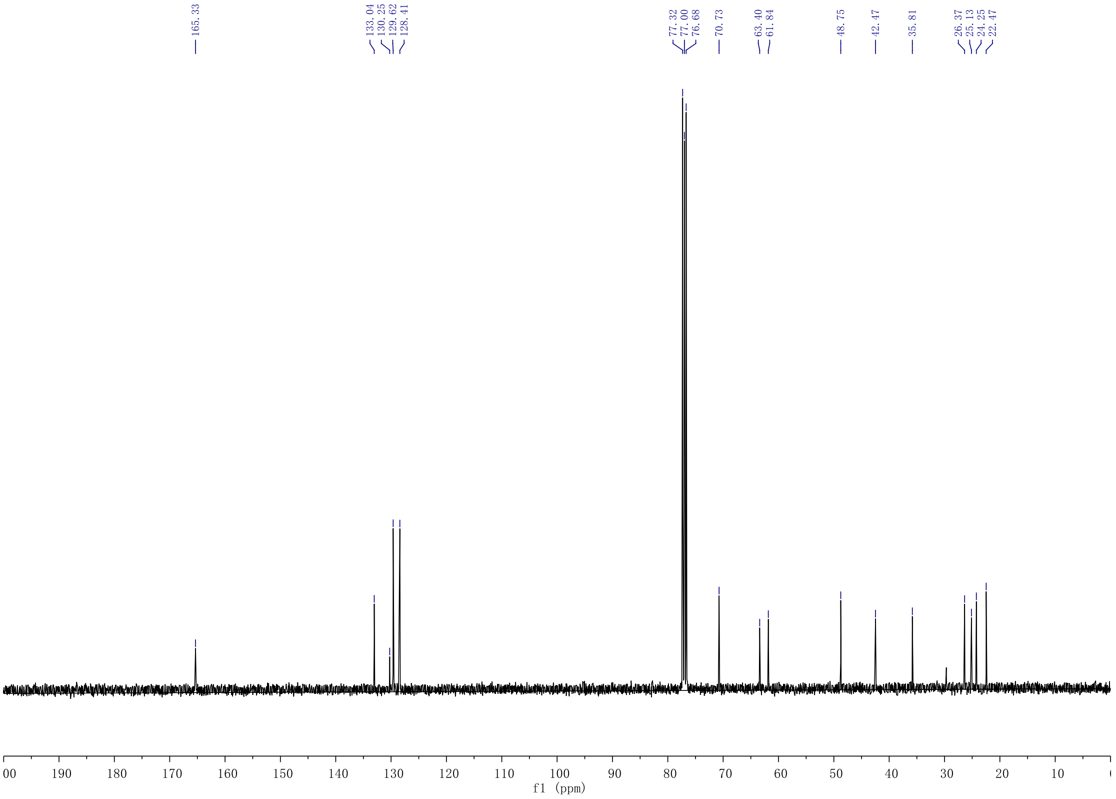
165.33
133.04
130.25
129.62
128.41
77.32
77.00
76.68
70.73
63.40
61.84
48.75
42.47
35.81
26.37
25.13
24.25
22.47
f1 (ppm)

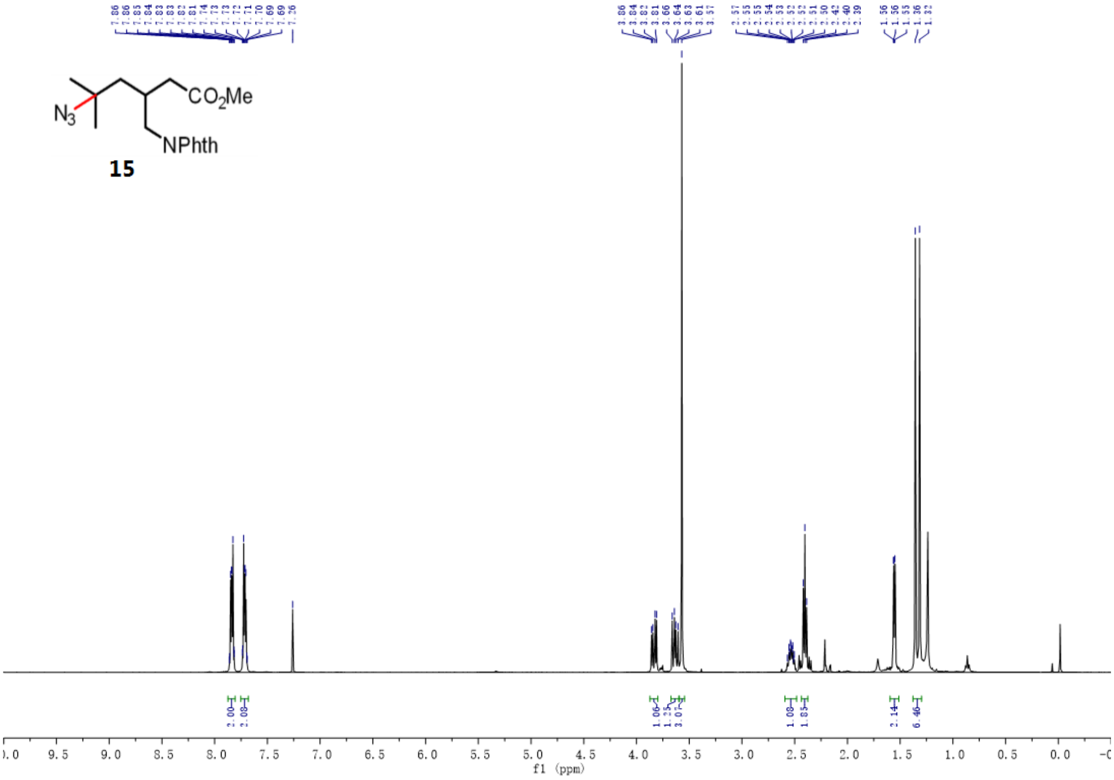

$N_3$
$CO_2Me$
NPhth
15
f1 (ppm)


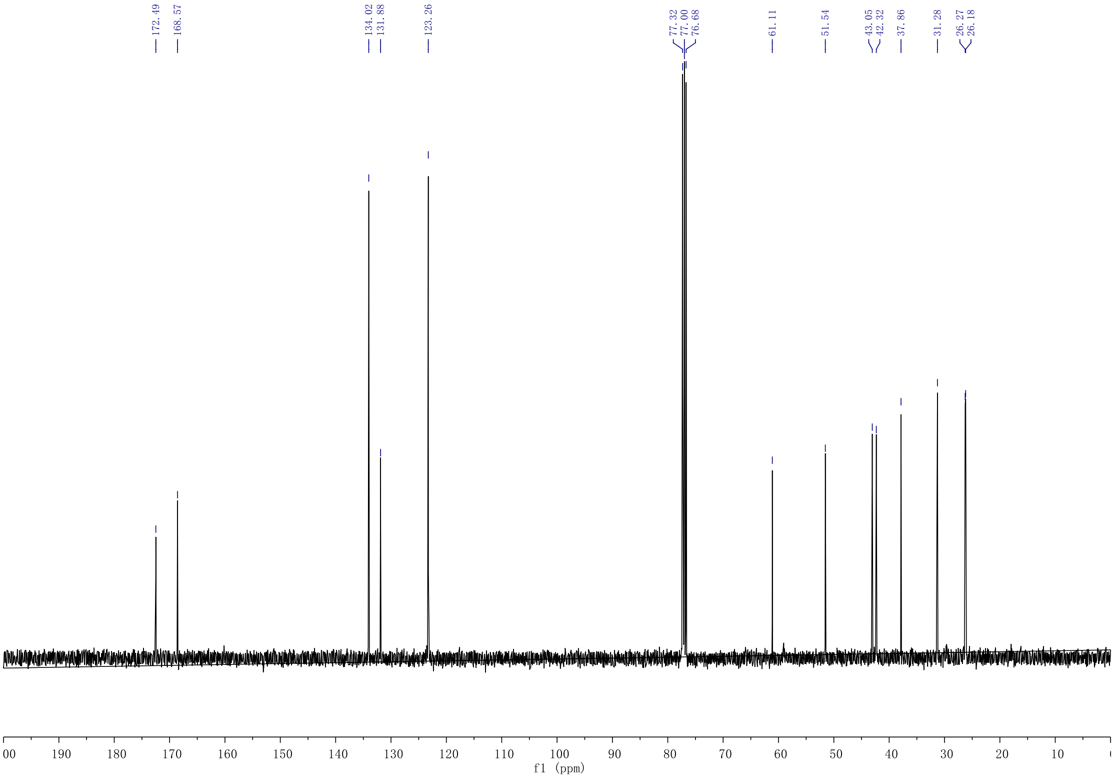

172.49
168.57
134.02
131.88
123.26
77.32
77.00
76.68
61.11
51.54
43.05
42.32
37.86
31.28
26.27
26.18
f1 (ppm)

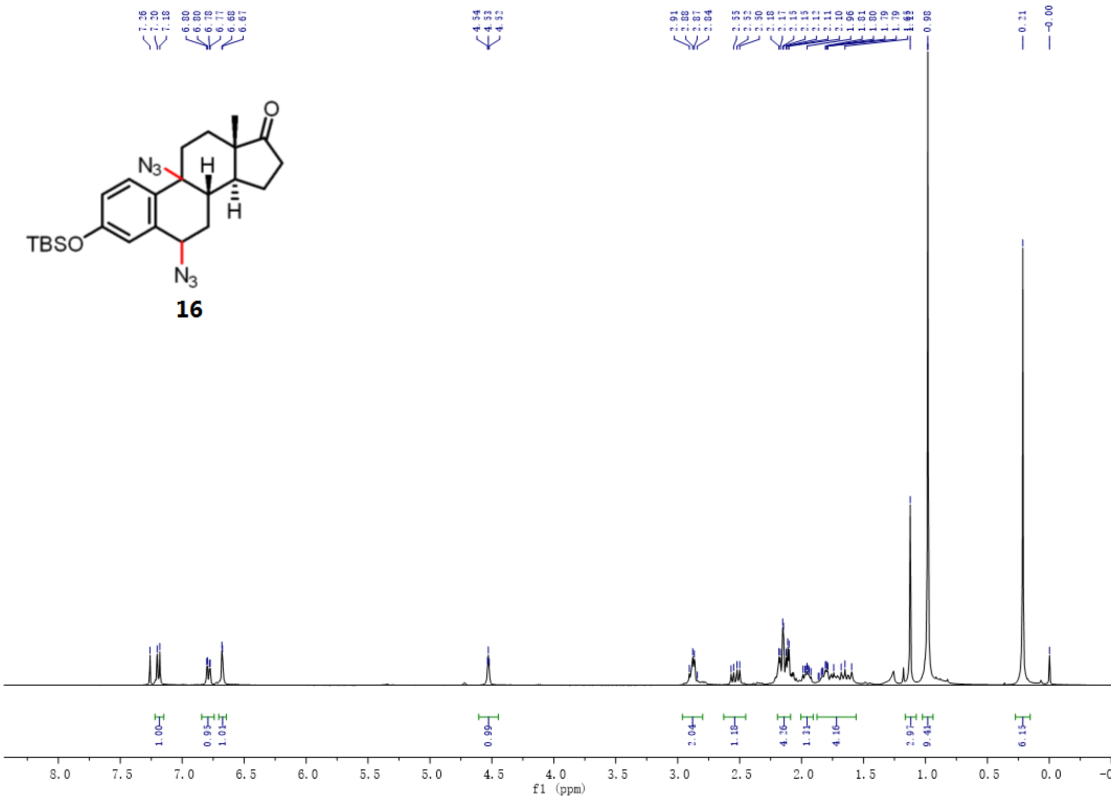
7.26
7.20
7.18
6.80
6.80
6.78
6.77
6.68
6.67
4.54
4.53
4.52
2.91
2.88
2.87
2.84
2.55
2.53
2.50
0.98
0.21
-0.00
N3
H
H
O
TBSO
N3
16
1.00
0.95
1.01
0.99
2.04
1.18
4.26
1.11
4.16
2.97
9.41
6.15
8.0
7.5
7.0
6.5
6.0
5.5
5.0
4.5
4.0
3.5
3.0
2.5
2.0
1.5
1.0
0.5
0.0
f1 (ppm)

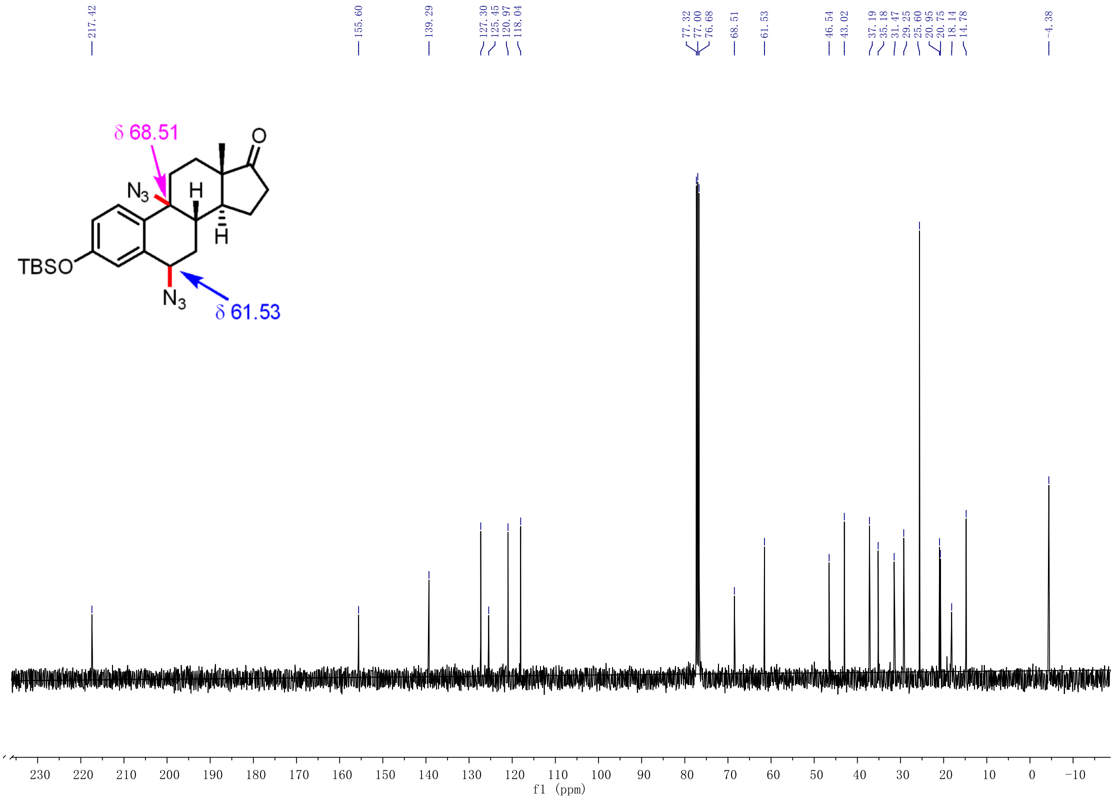
217.42
155.60
139.29
127.30
125.45
120.97
118.04
77.32
77.00
76.68
68.51
61.53
46.54
43.02
37.19
35.18
31.47
29.25
25.60
20.95
20.75
18.14
14.78
-4.38
δ 68.51
N3
H
H
O
TBSO
N3
δ 61.53
230
220
210
200
190
180
170
160
150
140
130
120
110
100
90
80
70
60
50
40
30
20
10
0
-10
f1 (ppm)

C13DEPT135

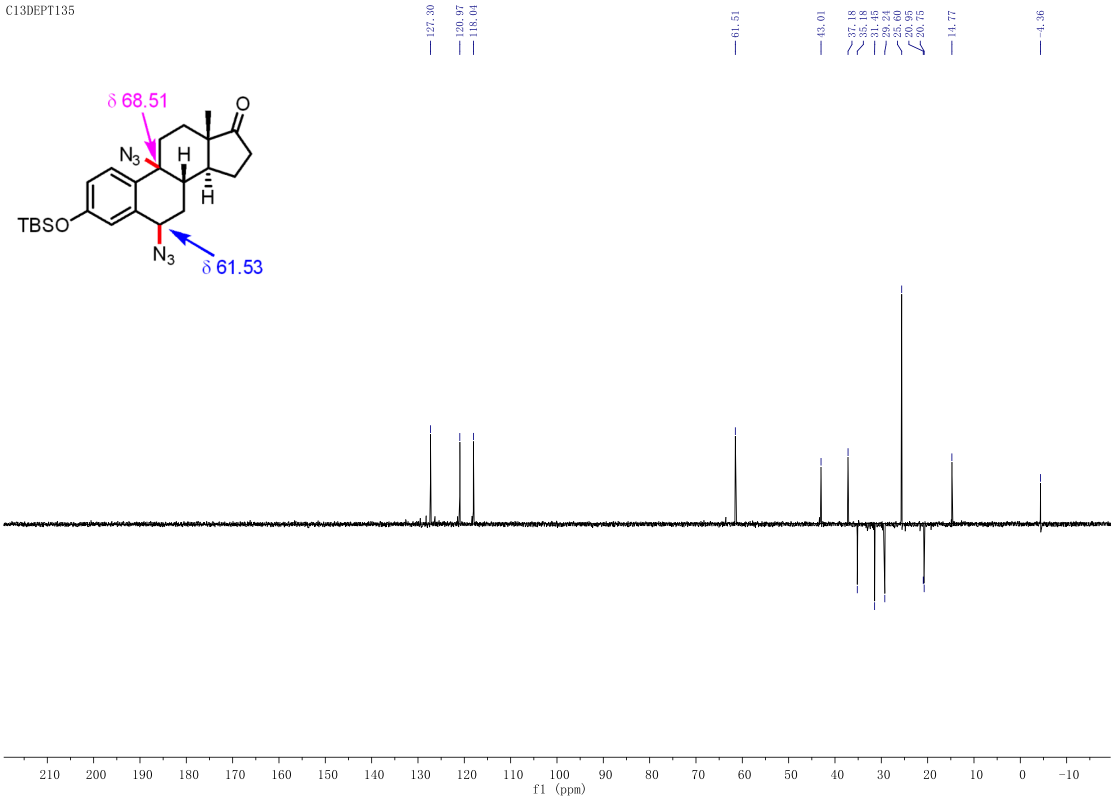


C13DEPT90

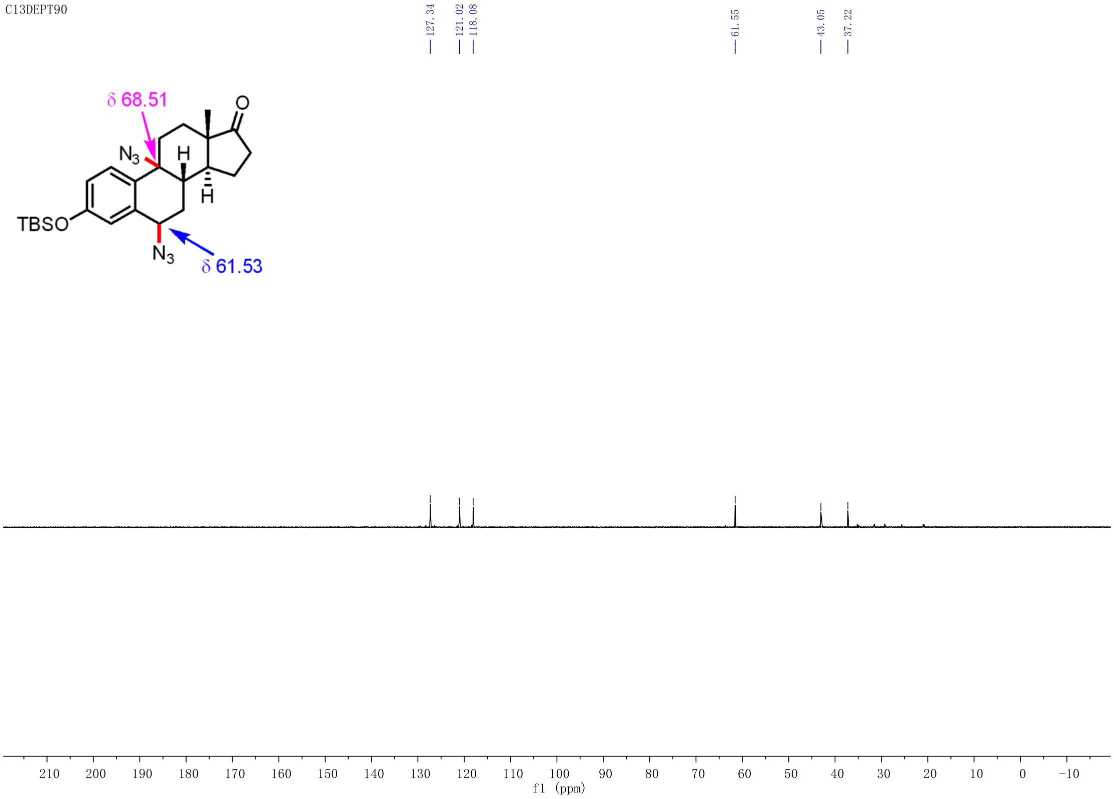

**17-1**

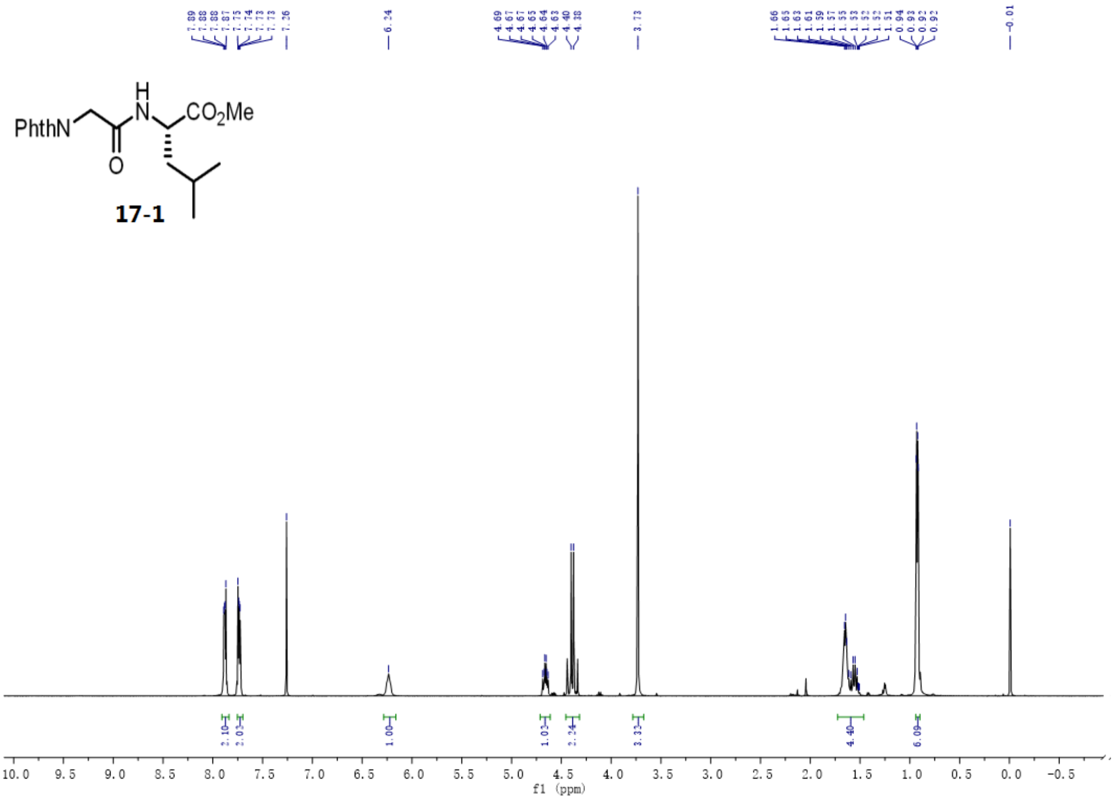
7.89
7.88
7.87
7.75
7.74
7.73
7.73
7.26
6.24
4.69
4.67
4.67
4.65
4.64
4.63
4.40
4.38
3.73
1.66
1.65
1.63
1.61
1.59
1.57
1.55
1.53
1.52
1.52
1.51
0.94
0.93
0.92
0.92
-0.01
2.10
2.03
1.00
1.03
2.24
3.33
4.40
6.09
f1 (ppm)

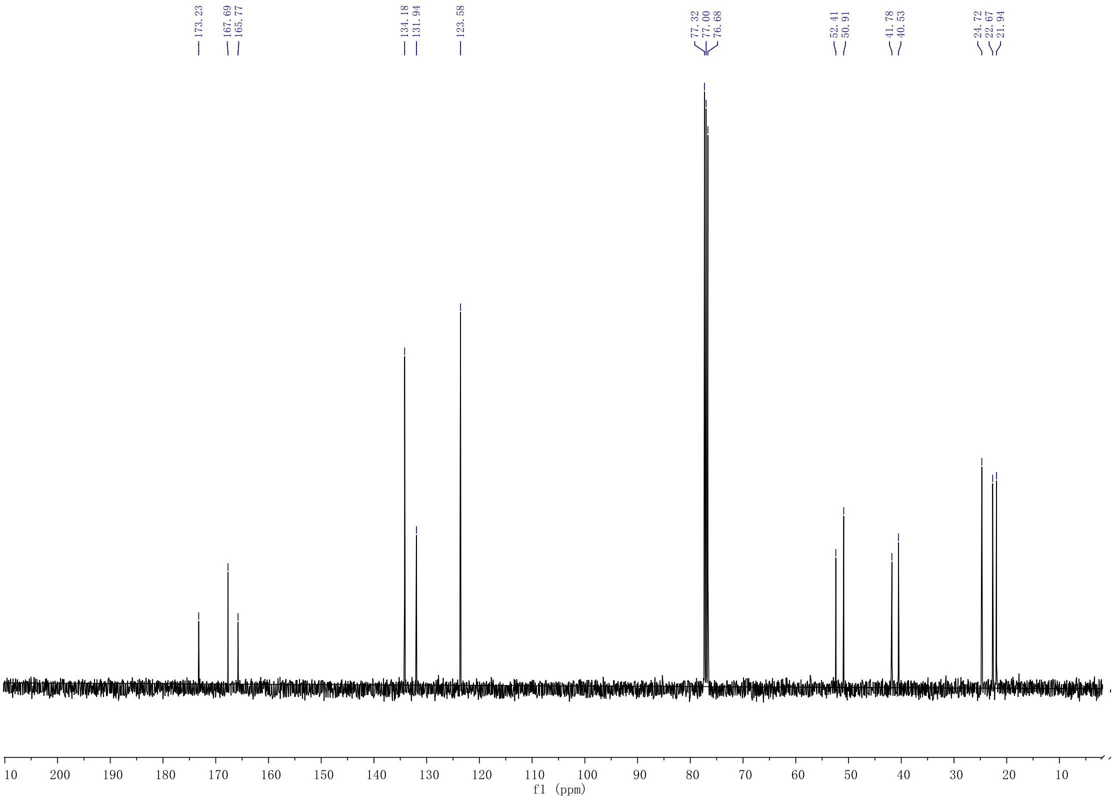
173.23
167.69
165.77
134.18
131.94
123.58
77.32
77.00
76.68
52.41
50.91
41.78
40.53
24.72
22.67
21.94
f1 (ppm)

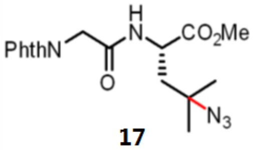

17

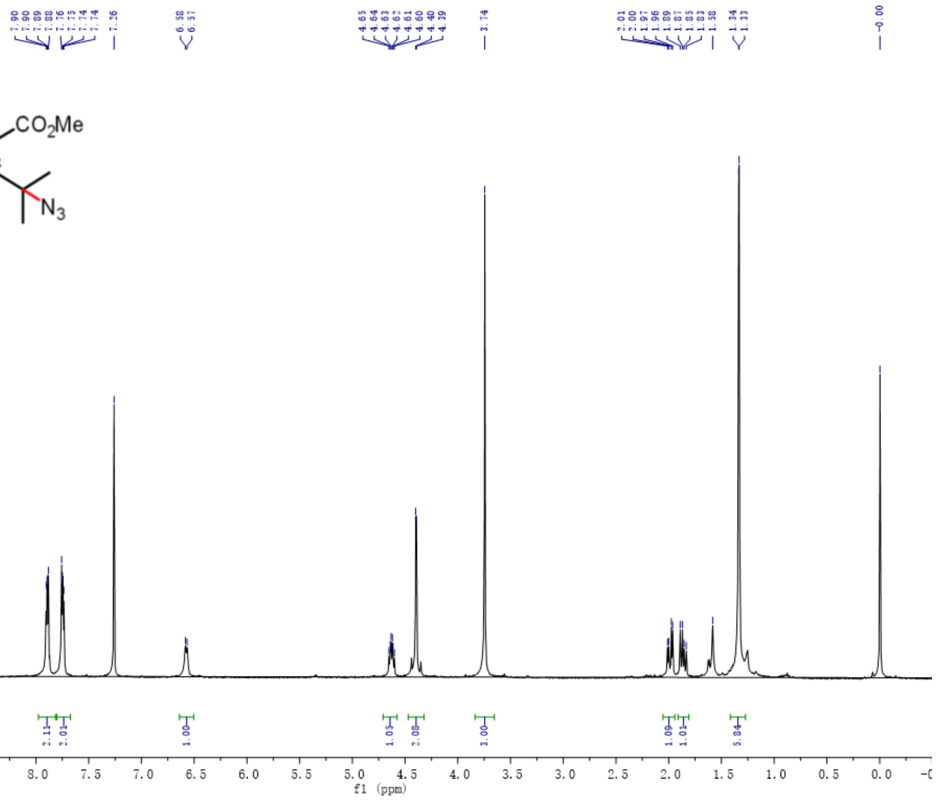

7.90
7.90
7.89
7.88
7.76
7.75
7.74
7.74
7.26
6.58
6.57
4.65
4.64
4.63
4.62
4.61
4.60
4.40
4.39
3.74
2.01
2.00
1.97
1.96
1.89
1.87
1.85
1.83
1.58
1.34
1.33
0.00
2.11
2.01
1.00
1.05
2.08
3.00
1.09
1.01
5.84
f1 (ppm)


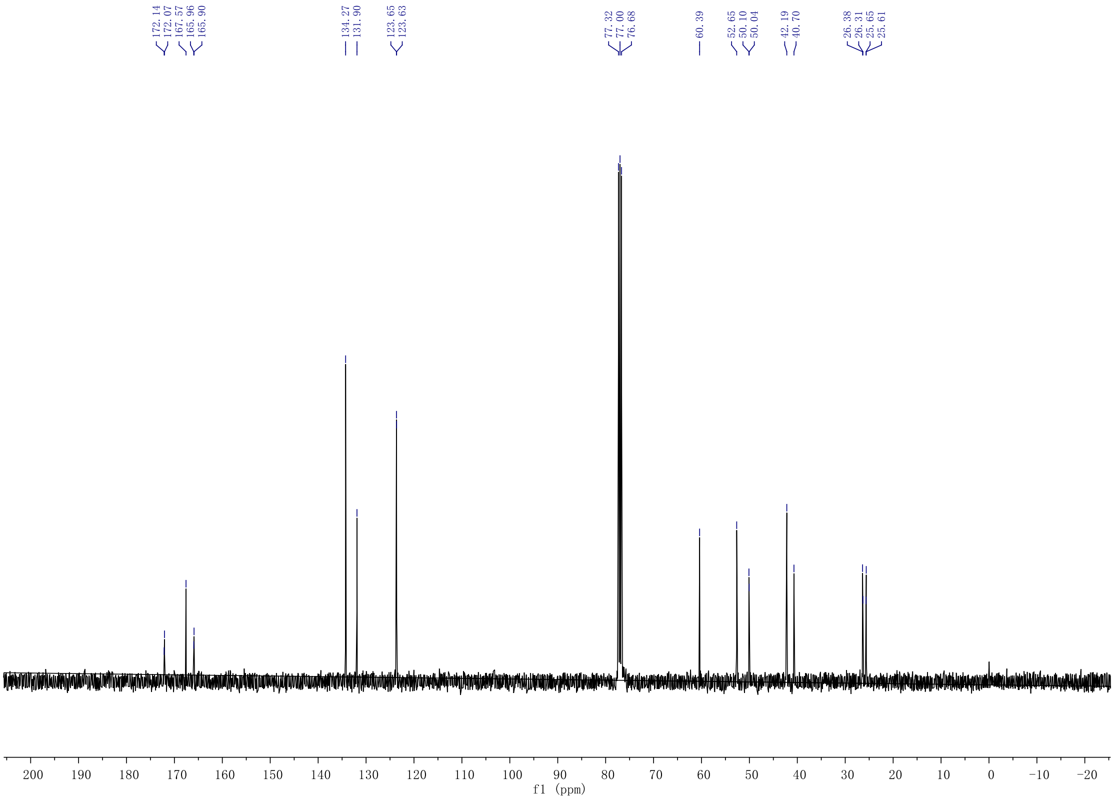

172.14
172.07
167.57
165.96
165.90
134.27
131.90
123.65
123.63
77.32
77.00
76.68
60.39
52.65
50.10
50.04
42.19
40.70
26.38
26.31
25.65
25.61
f1 (ppm)

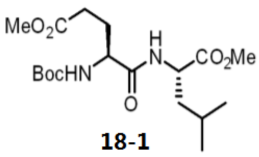
MeO2C
H
N
CO2Me
BocHN
O

**18-1**

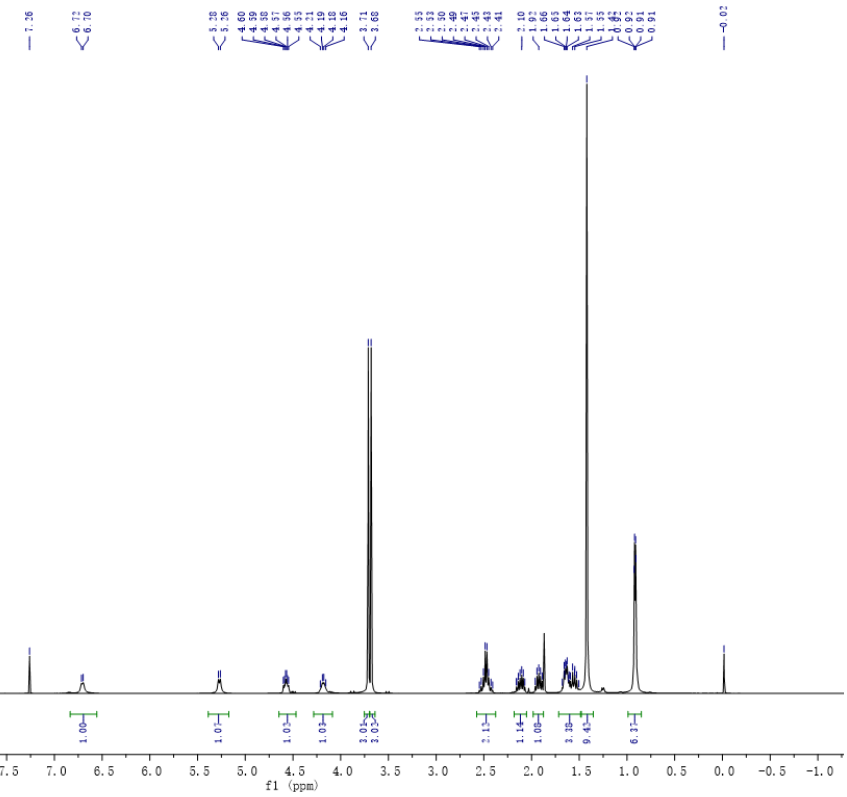
f1 (ppm)

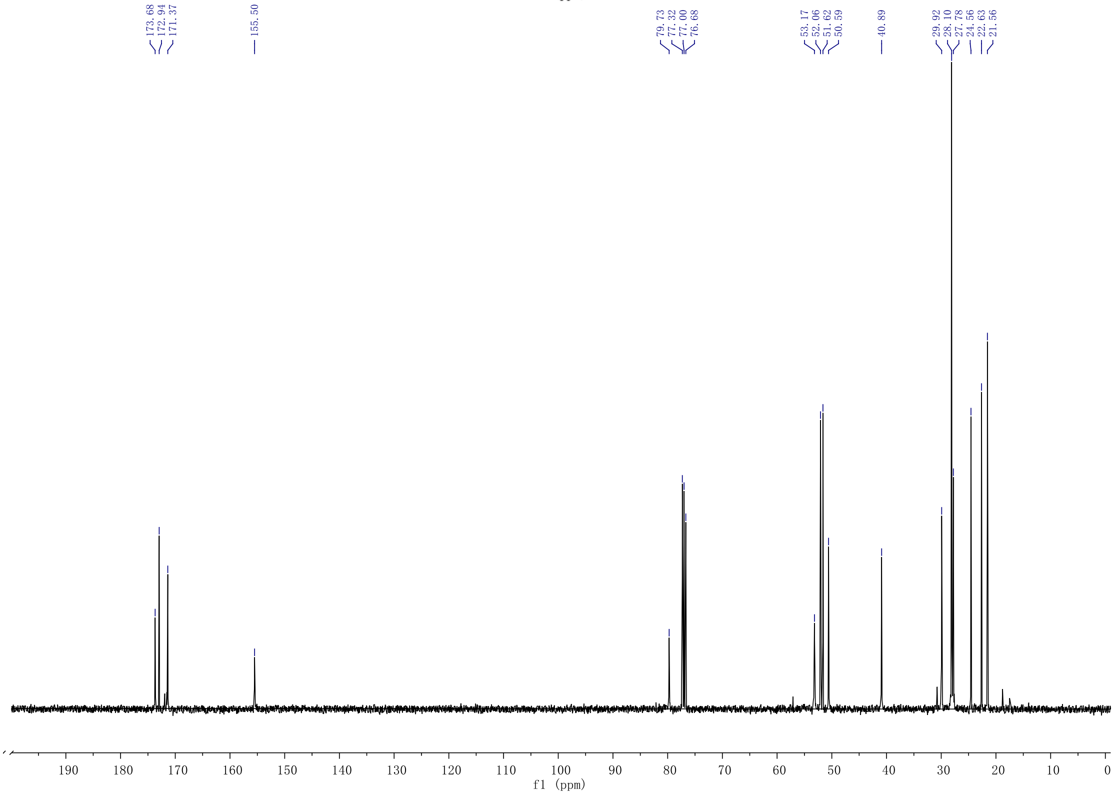
173.68
172.94
171.37
155.50
79.73
77.32
77.00
76.68
53.17
52.06
51.62
50.59
40.89
29.92
28.10
27.78
24.56
22.63
21.56
f1 (ppm)

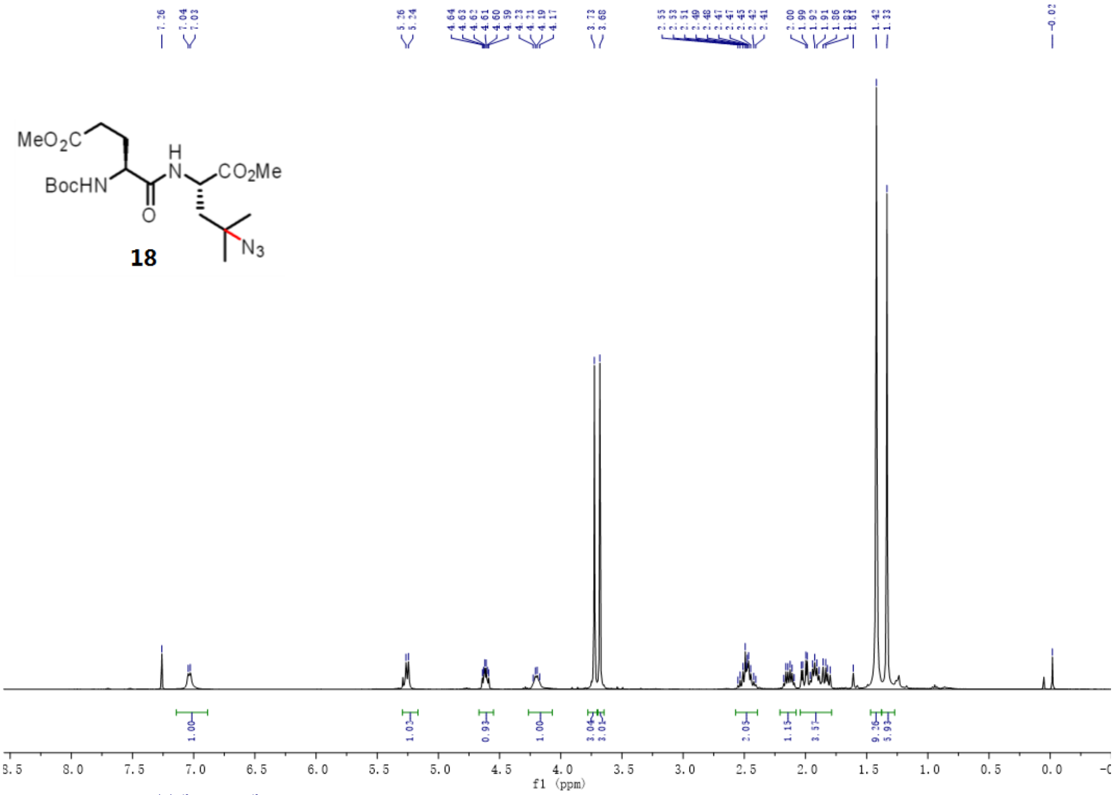

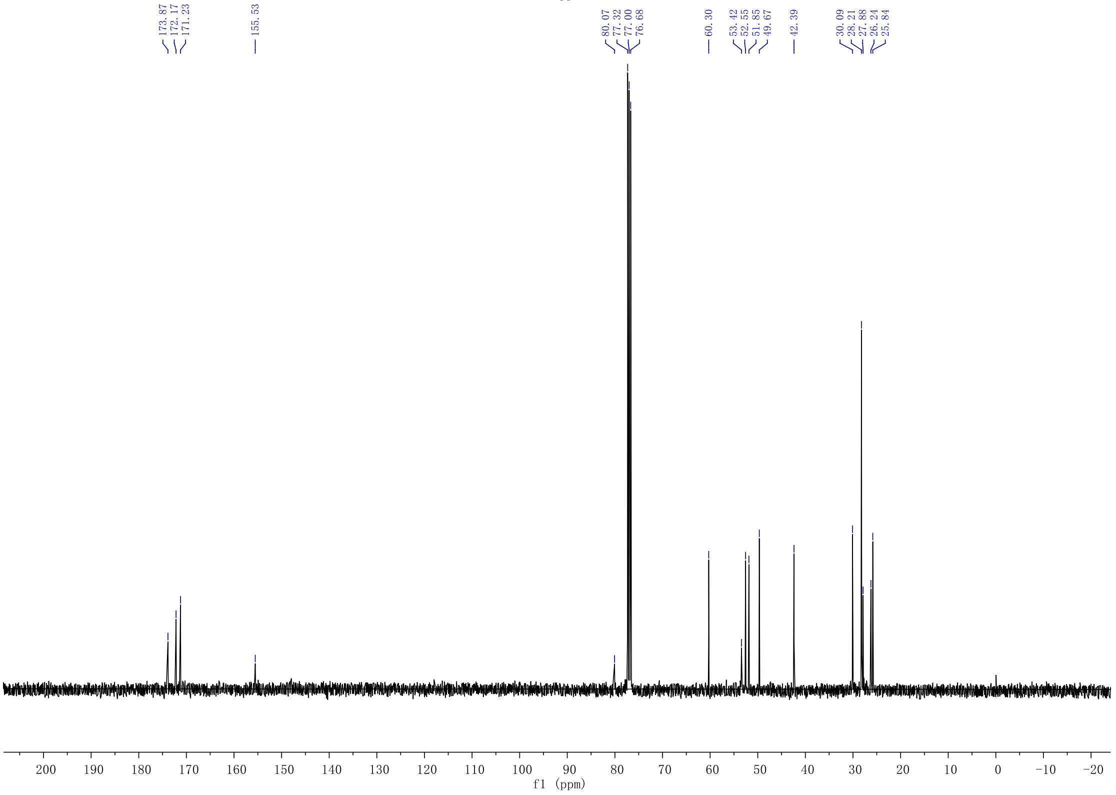

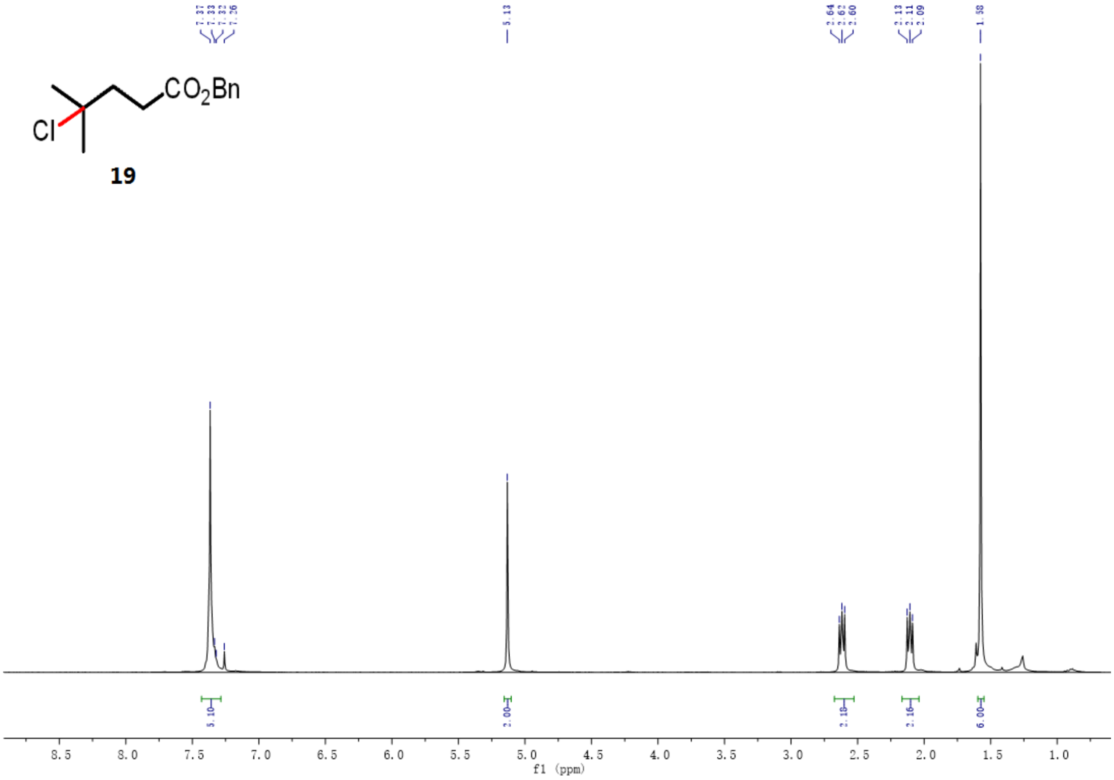
Cl
CO2Bn
19
7.37
7.33
7.32
7.26
5.13
2.64
2.62
2.60
2.13
2.11
2.09
1.58
5.10
2.00
2.18
2.16
6.00
8.5 8.0 7.5 7.0 6.5 6.0 5.5 5.0 4.5 4.0 3.5 3.0 2.5 2.0 1.5 1.0
f1 (ppm)

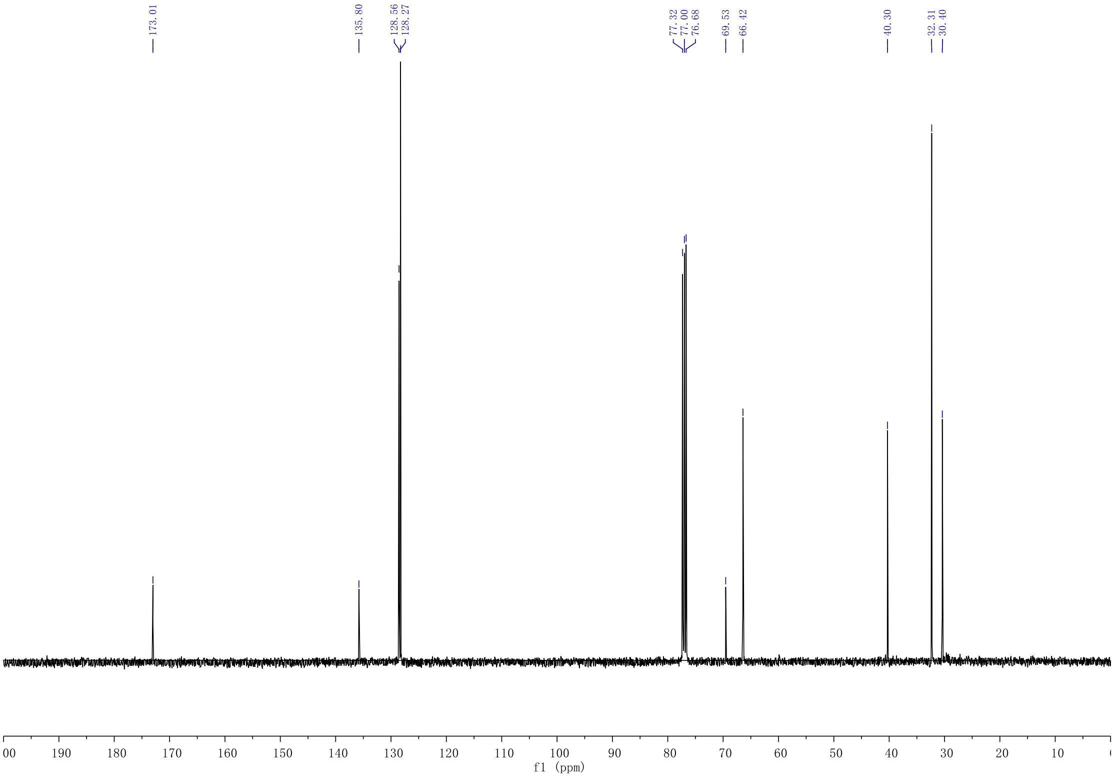
173.01
135.80
128.56
128.27
77.32
77.00
76.68
69.53
66.42
40.30
32.31
30.40
00 190 180 170 160 150 140 130 120 110 100 90 80 70 60 50 40 30 20 10
f1 (ppm)

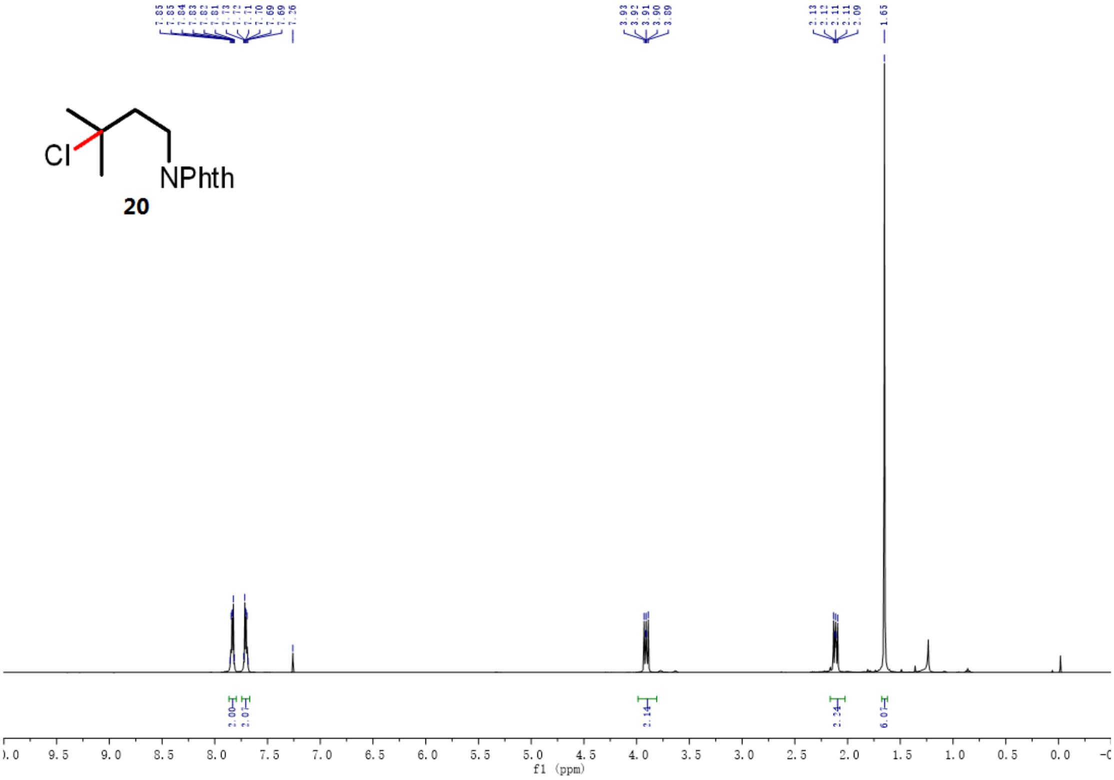
7.85
7.85
7.84
7.83
7.82
7.81
7.73
7.72
7.71
7.70
7.69
7.69
7.26
3.93
3.92
3.91
3.90
3.89
2.13
2.12
2.11
2.11
2.09
1.65
Cl
NPhth
20
2.00
2.07
2.14
2.34
6.07
f1 (ppm)

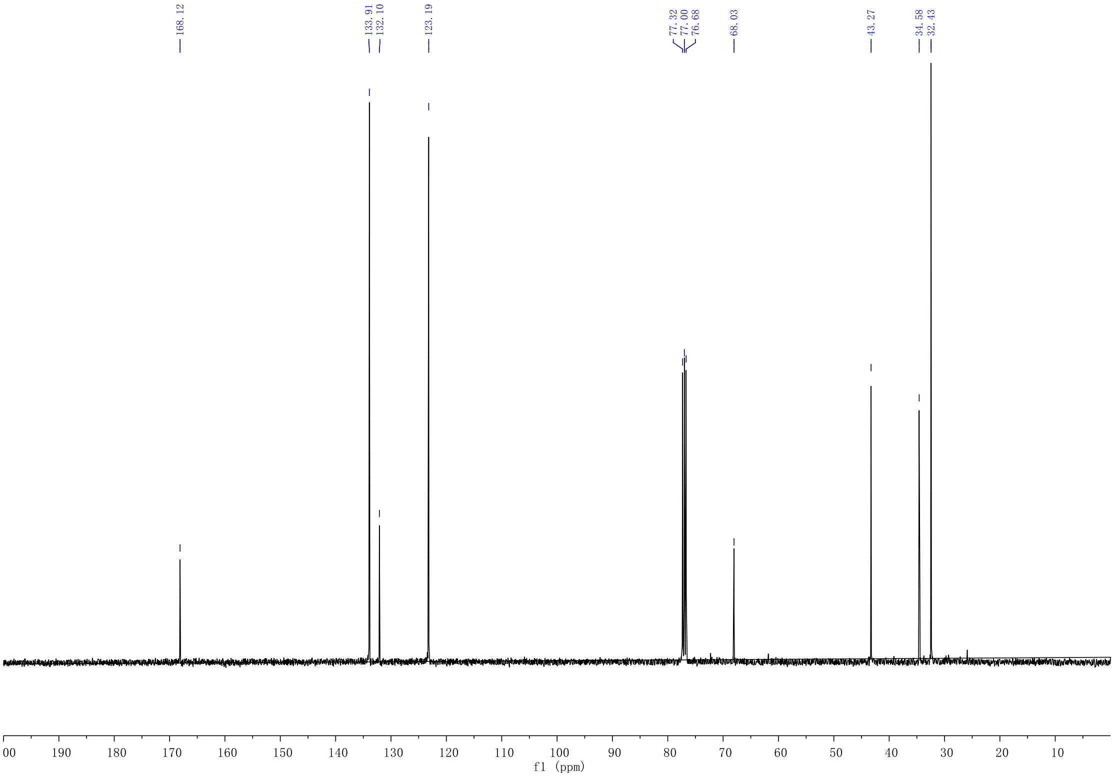
168.12
133.91
132.10
123.19
77.32
77.00
76.68
68.03
43.27
34.58
32.43
f1 (ppm)

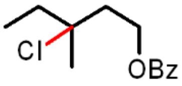

**21**

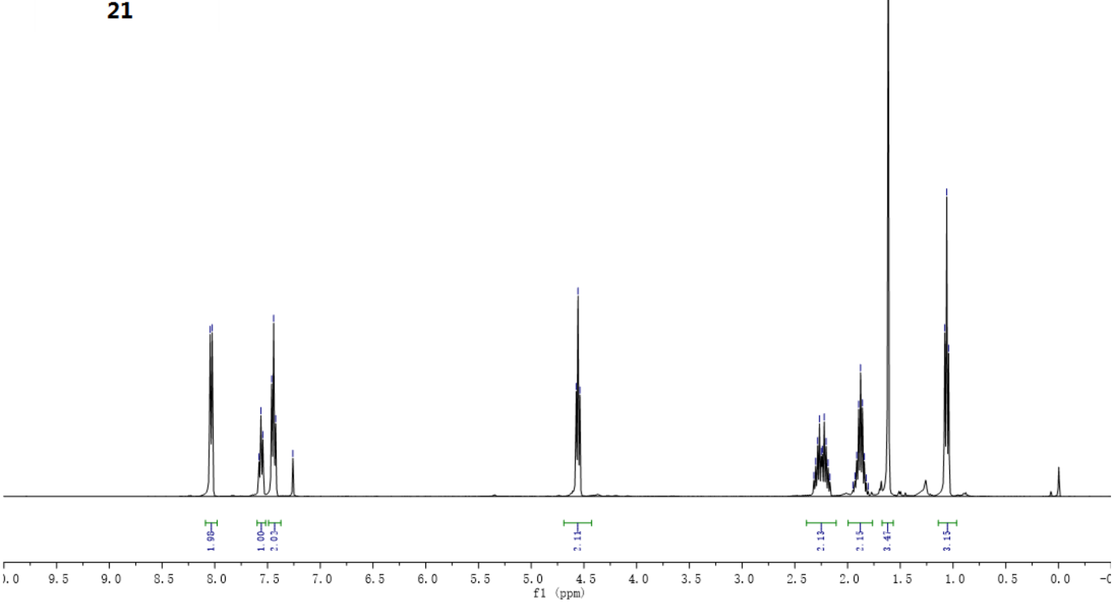
8.04
8.02
7.58
7.56
7.54
7.46
7.44
7.42
7.26
4.57
4.56
4.54
1.98
1.00
2.03
2.11
2.13
2.15
3.47
3.15
f1 (ppm)

VV

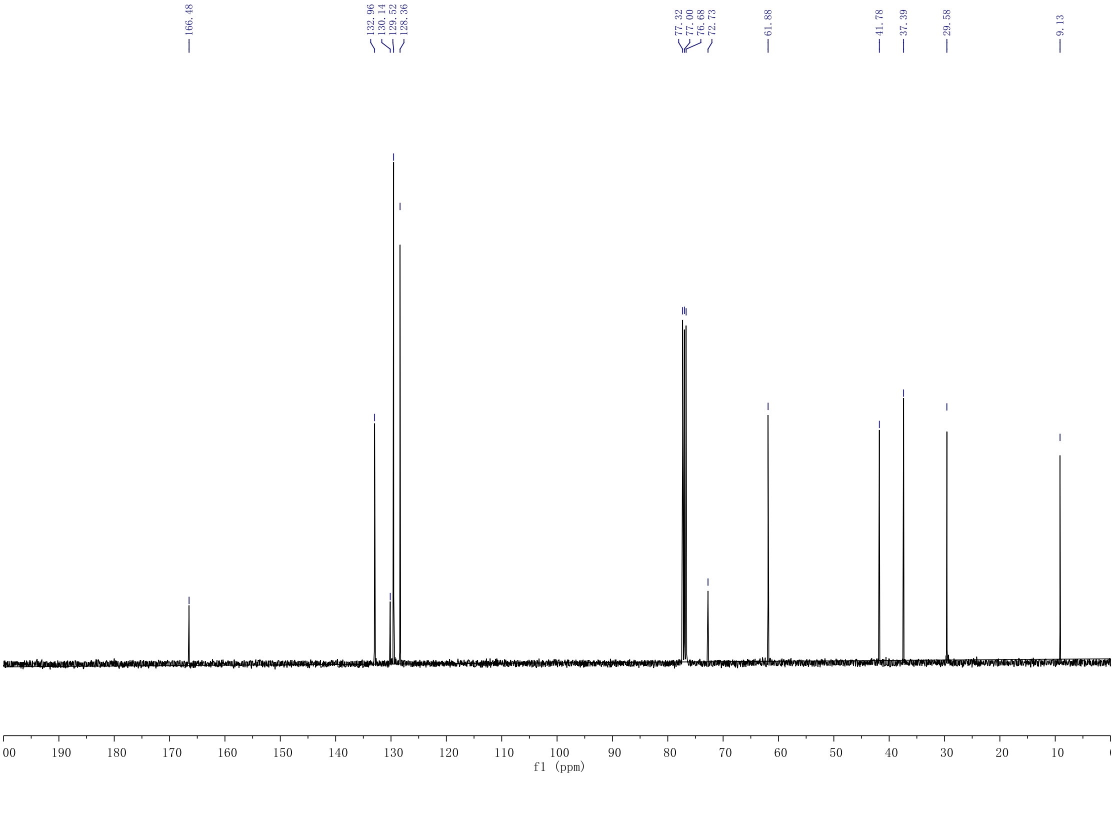
166.48
132.96
130.14
129.52
128.36
77.32
77.00
76.68
72.73
61.88
41.78
37.39
29.58
9.13
f1 (ppm)

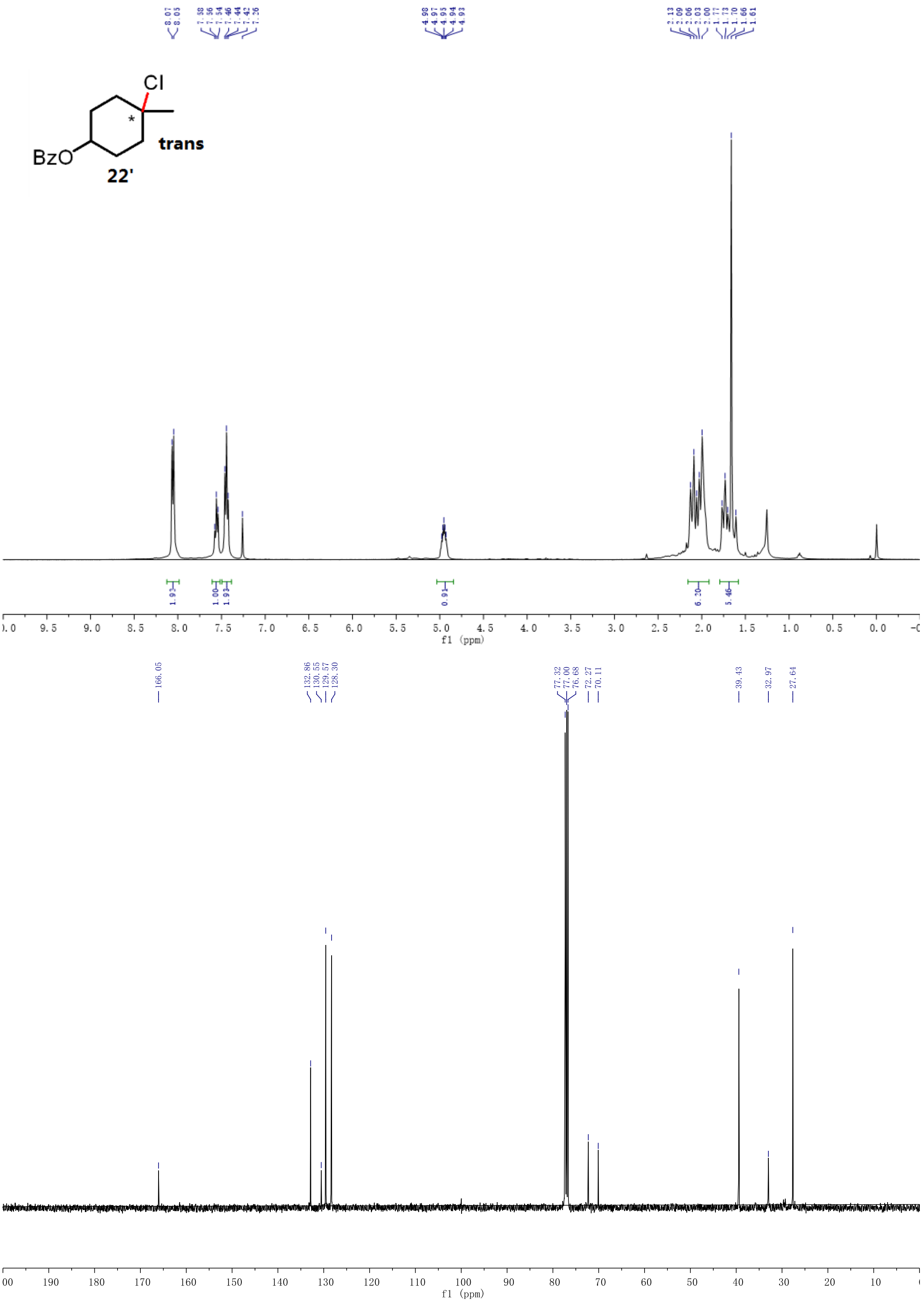

Cl
BzO
trans
22'
8.07
8.05
7.58
7.56
7.54
7.46
7.44
7.42
7.36
4.98
4.97
4.95
4.94
4.93
2.13
2.09
2.06
2.03
2.00
1.77
1.73
1.70
1.66
1.61
1.93
1.00
1.93
0.91
6.20
5.46
f1 (ppm)
166.05
132.86
130.55
129.57
128.30
77.32
77.00
76.68
72.27
70.11
39.43
32.97
27.64
f1 (ppm)

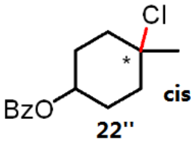

Cl
BzO
*
cis


22''

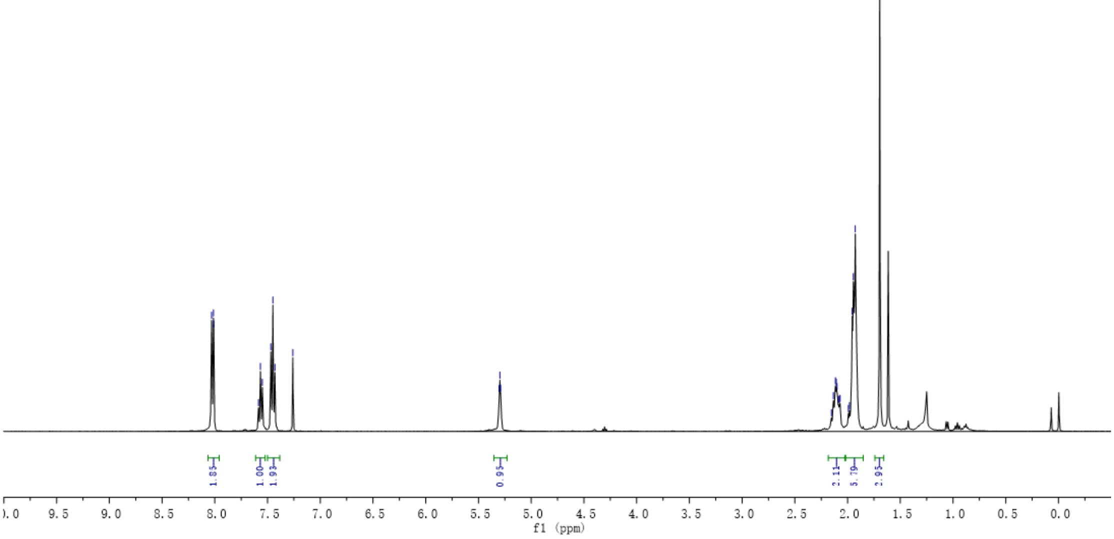

1.85
1.00
1.93
0.95
2.11
5.79
2.95
f1 (ppm)


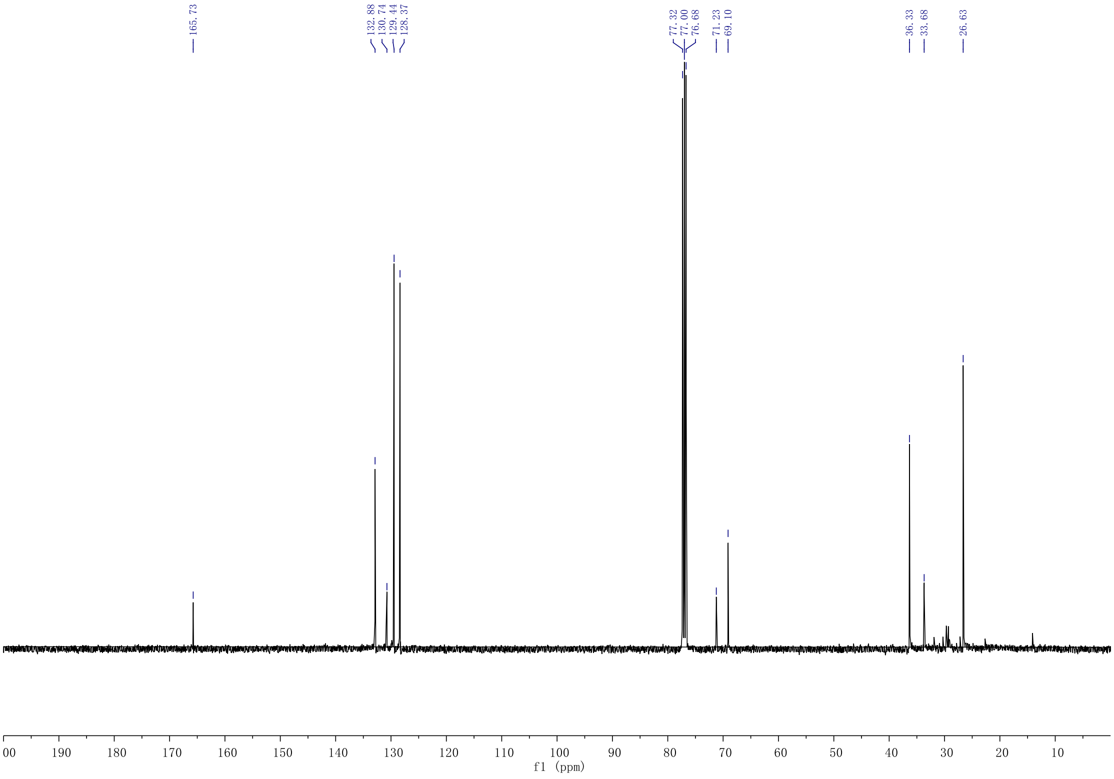

165.73
132.88
130.74
129.44
128.37
77.32
77.00
76.68
71.23
69.10
36.33
33.68
26.63
f1 (ppm)

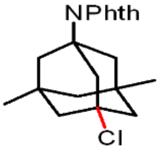
NPhth
Cl

**23**

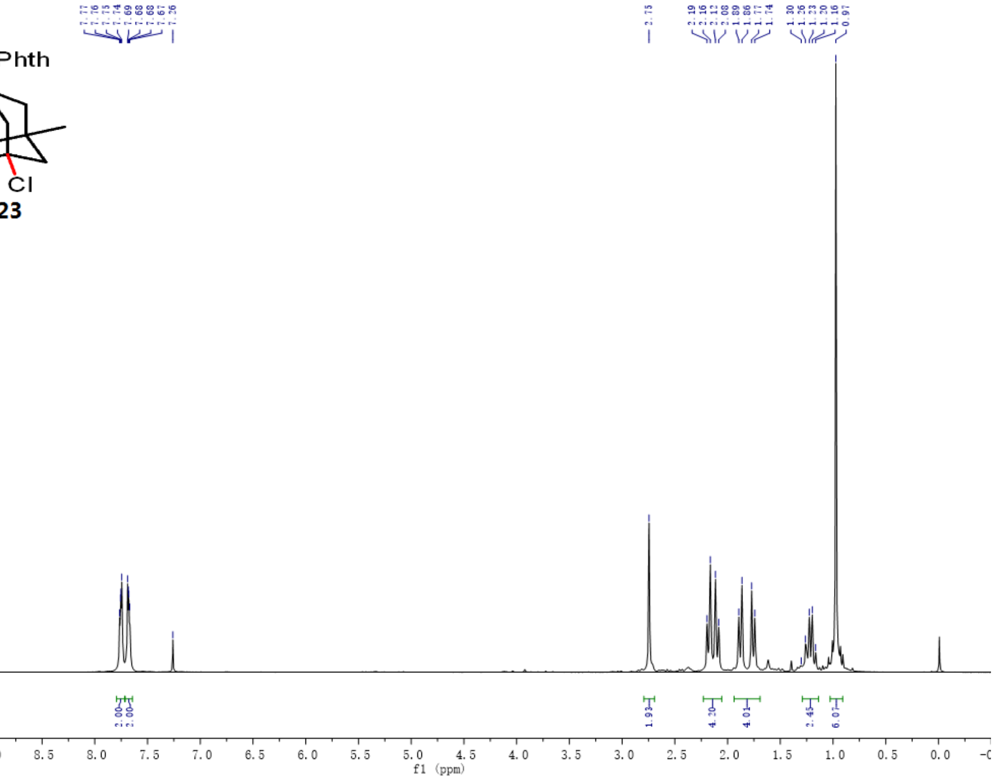
7.77
7.76
7.73
7.74
7.69
7.68
7.68
7.67
7.26
2.75
2.19
2.16
2.12
2.08
1.89
1.86
1.77
1.74
1.30
1.26
1.23
1.20
1.16
0.97
2.00
2.00
1.93
4.20
4.01
2.45
6.07
f1 (ppm)

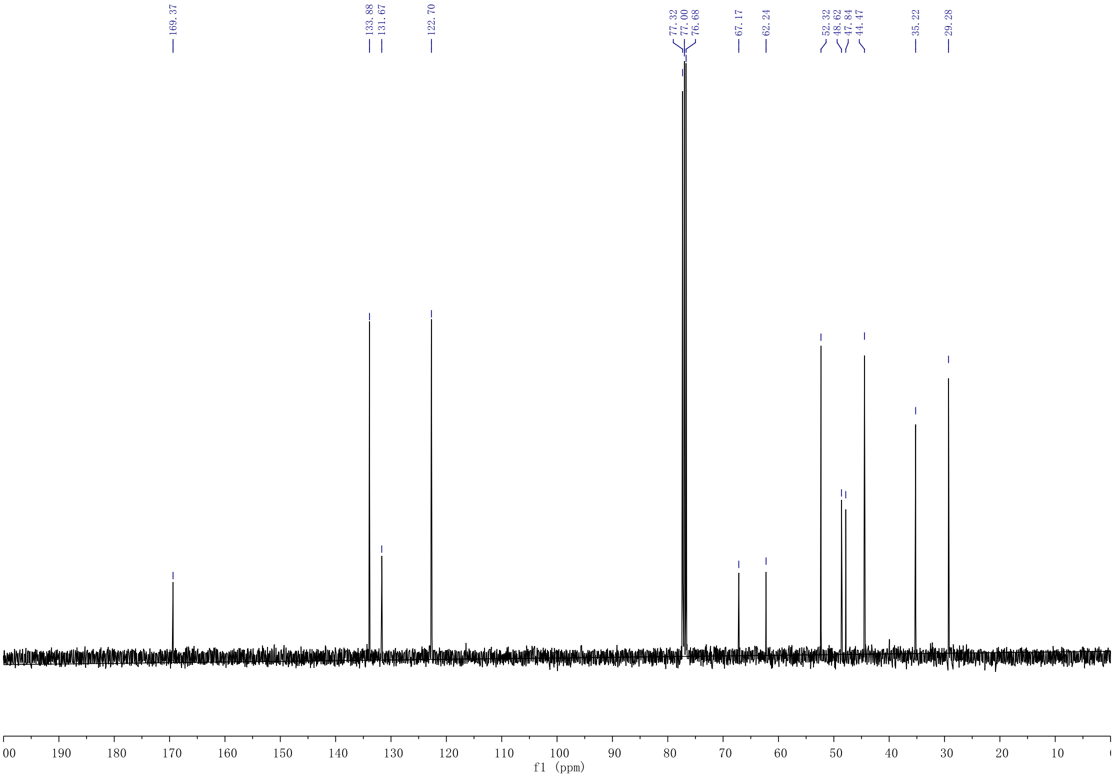
169.37
133.88
131.67
122.70
77.32
77.00
76.68
67.17
62.24
52.32
48.62
47.84
44.47
35.22
29.28
f1 (ppm)

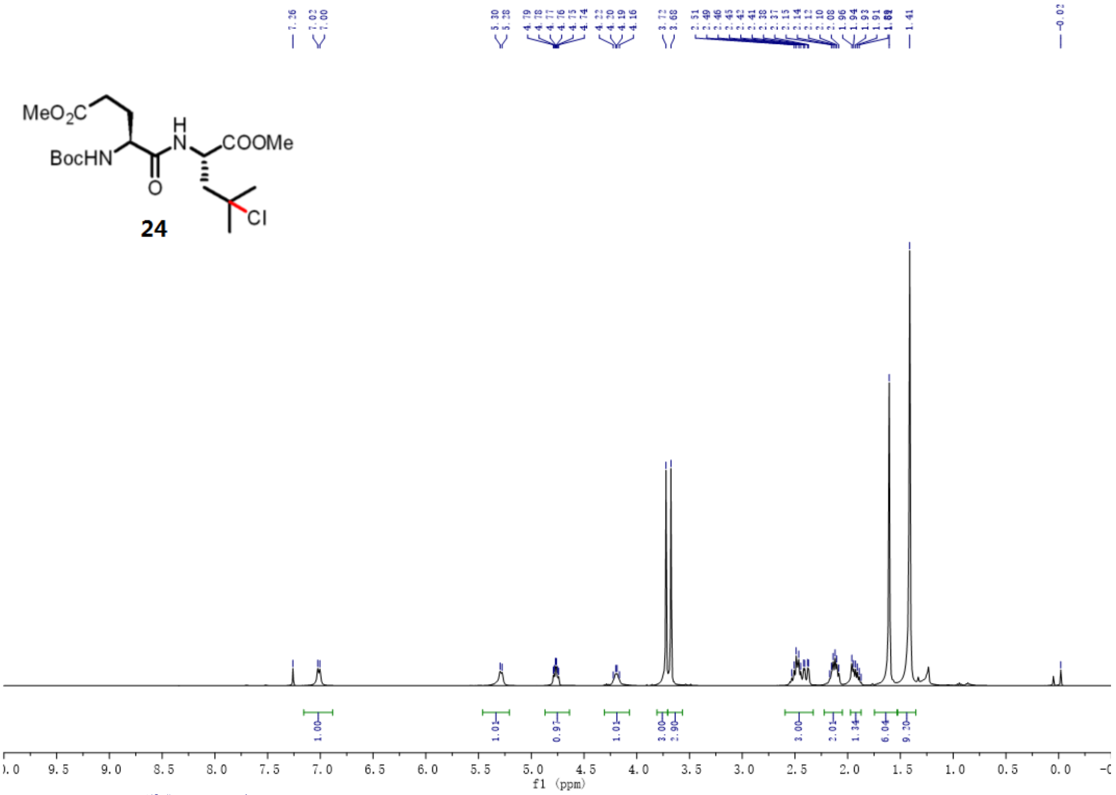

24

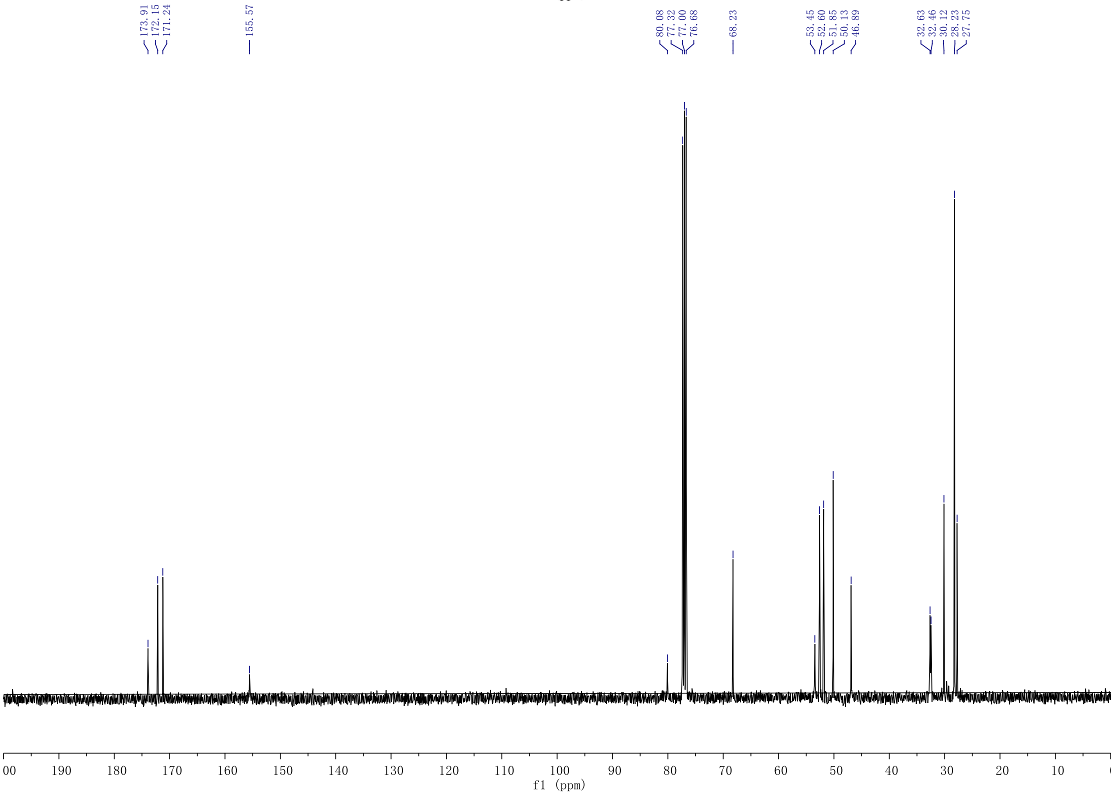

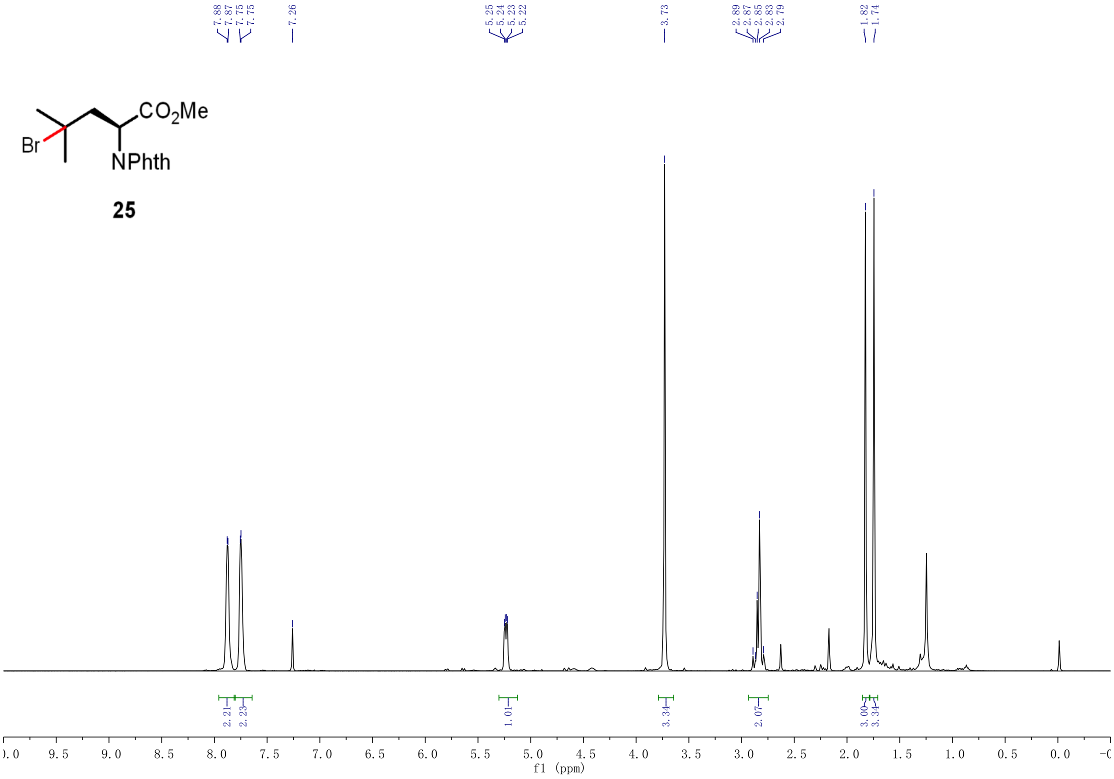


Br
$CO_2Me$
NPhth
25
7.88
7.87
7.75
7.75
7.26
5.25
5.24
5.23
5.22
3.73
2.89
2.87
2.85
2.83
2.79
1.82
1.74
2.21
2.23
1.01
3.34
2.07
3.00
3.34
f1 (ppm)


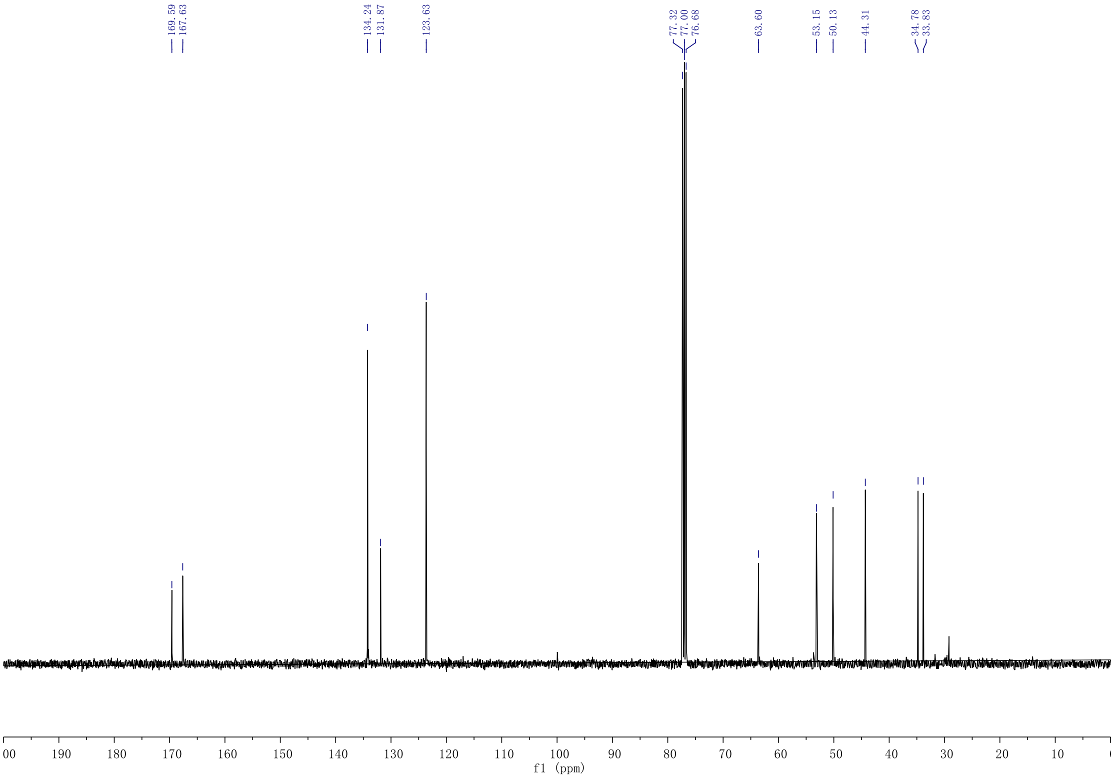

169.59
167.63
134.24
131.87
123.63
77.32
77.00
76.68
63.60
53.15
50.13
44.31
34.78
33.83
f1 (ppm)

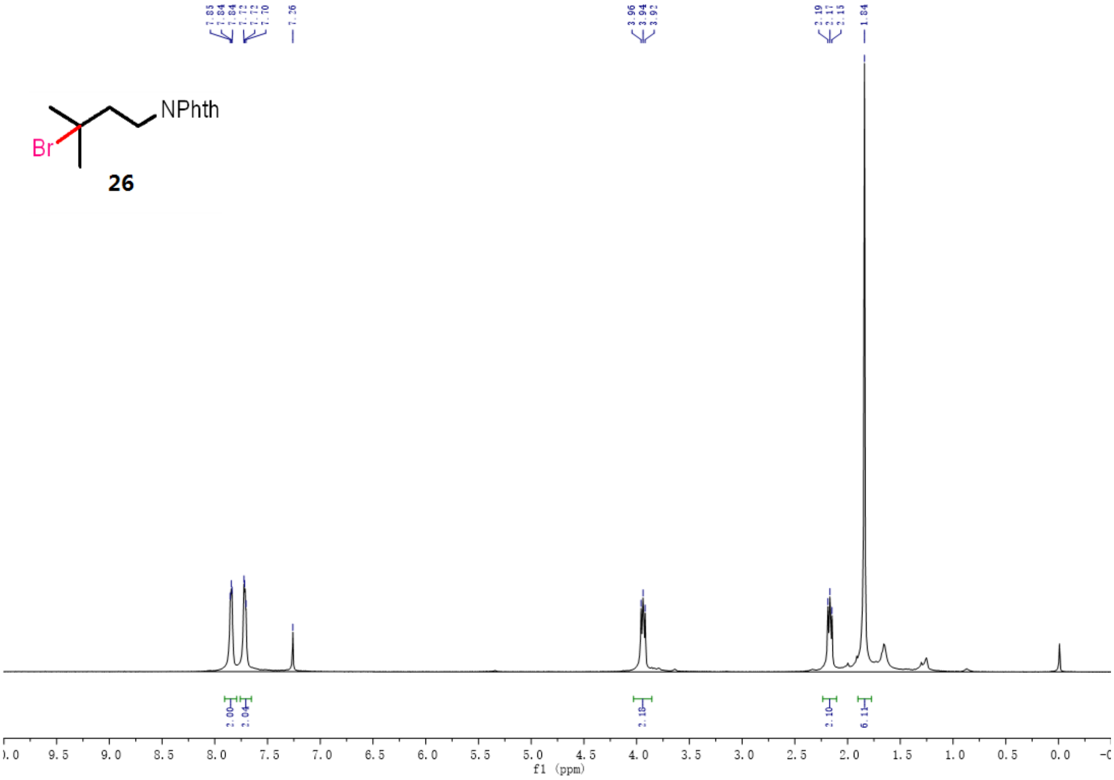
NPhth
Br
26
7.85
7.84
7.84
7.72
7.72
7.70
7.26
3.96
3.94
3.92
2.19
2.17
2.15
1.84
2.00
2.04
2.18
2.10
6.11
f1 (ppm)

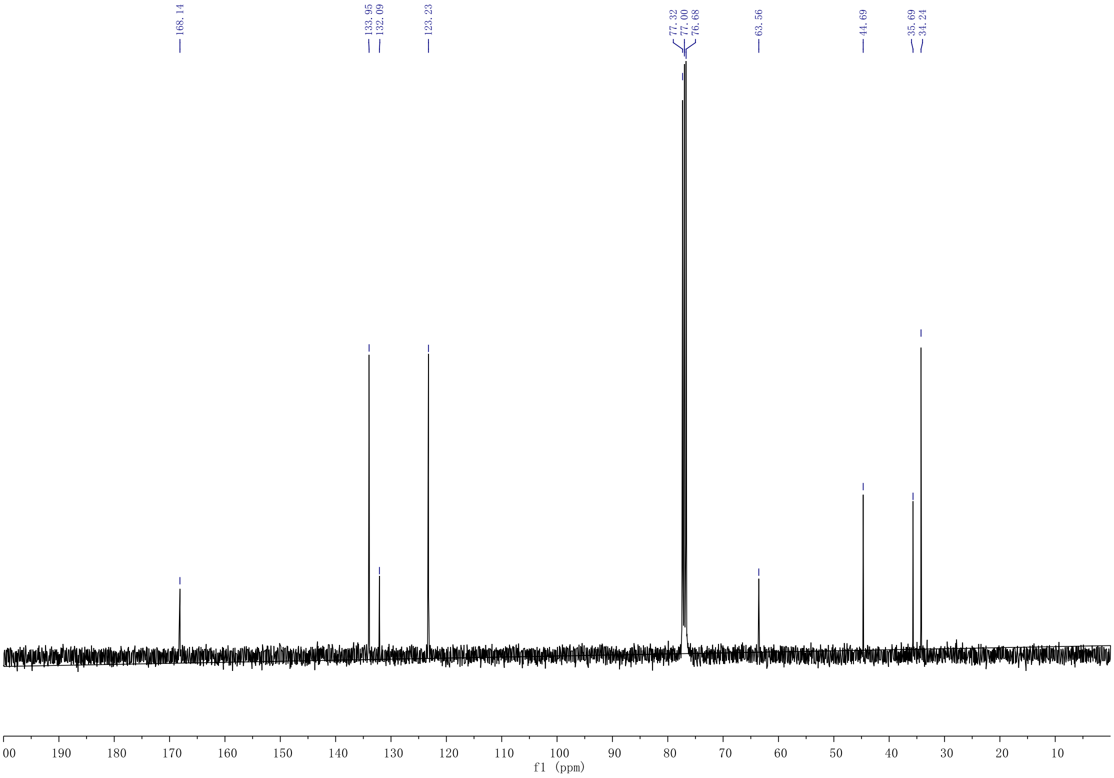
168.14
133.95
132.09
123.23
77.32
77.00
76.68
63.56
44.69
35.69
34.24
f1 (ppm)

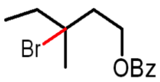
Br
OBz

**27**

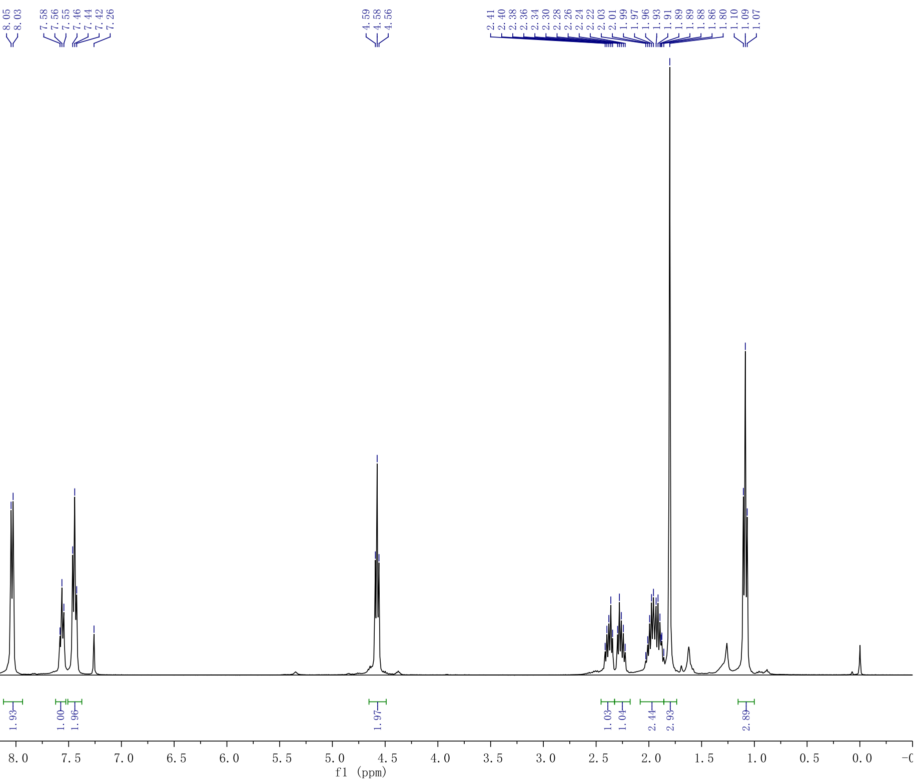
8.05
8.03
7.58
7.56
7.55
7.46
7.44
7.42
7.26
4.59
4.58
4.56
2.41
2.40
2.38
2.36
2.34
2.30
2.28
2.26
2.24
2.22
2.03
2.01
1.99
1.97
1.96
1.93
1.91
1.89
1.88
1.86
1.80
1.10
1.09
1.07
1.93
1.00
1.96
1.97
1.03
1.04
2.44
2.93
2.89
f1 (ppm)

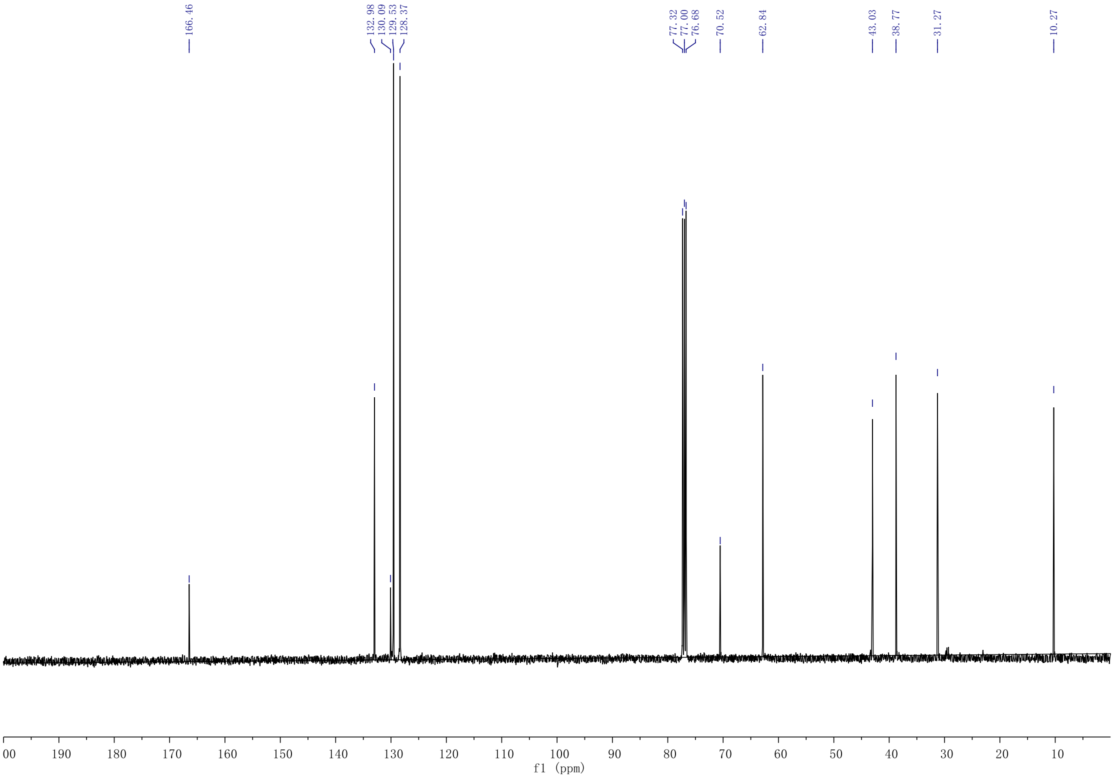
166.46
132.98
130.09
129.53
128.37
77.32
77.00
76.68
70.52
62.84
43.03
38.77
31.27
10.27
f1 (ppm)

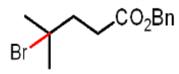
CO2Bn
Br

**28**

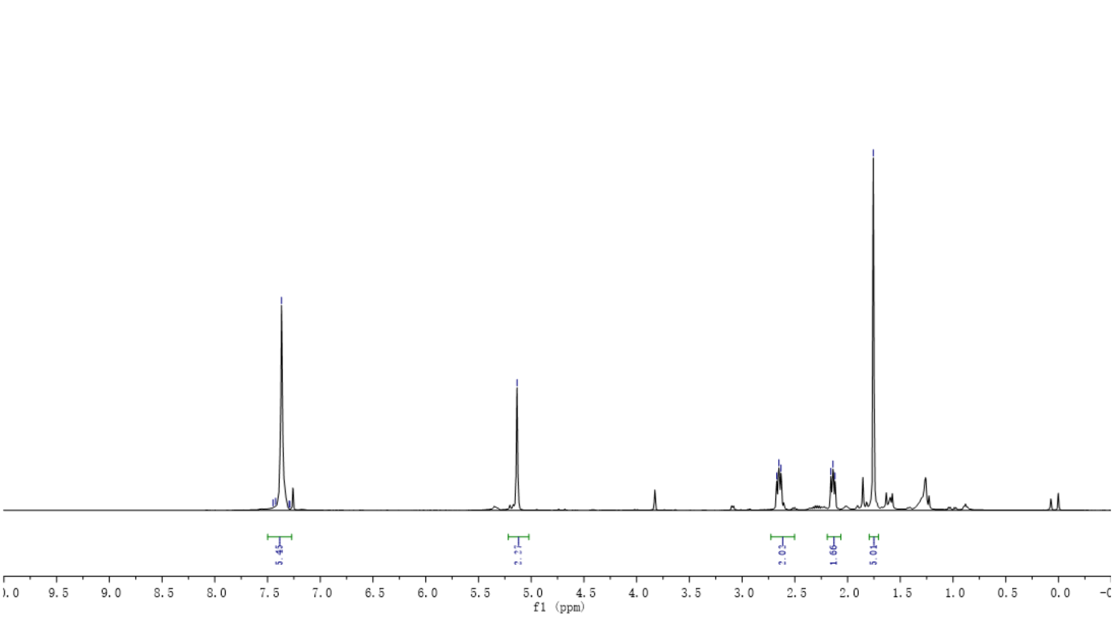
7.43
7.42
7.37
7.30
7.29
5.13
2.67
2.65
2.63
2.16
2.14
2.12
1.76
5.43
2.27
2.02
1.66
5.01
9.5
9.0
8.5
8.0
7.5
7.0
6.5
6.0
5.5
5.0
4.5
4.0
3.5
3.0
2.5
2.0
1.5
1.0
0.5
0.0
f1 (ppm)

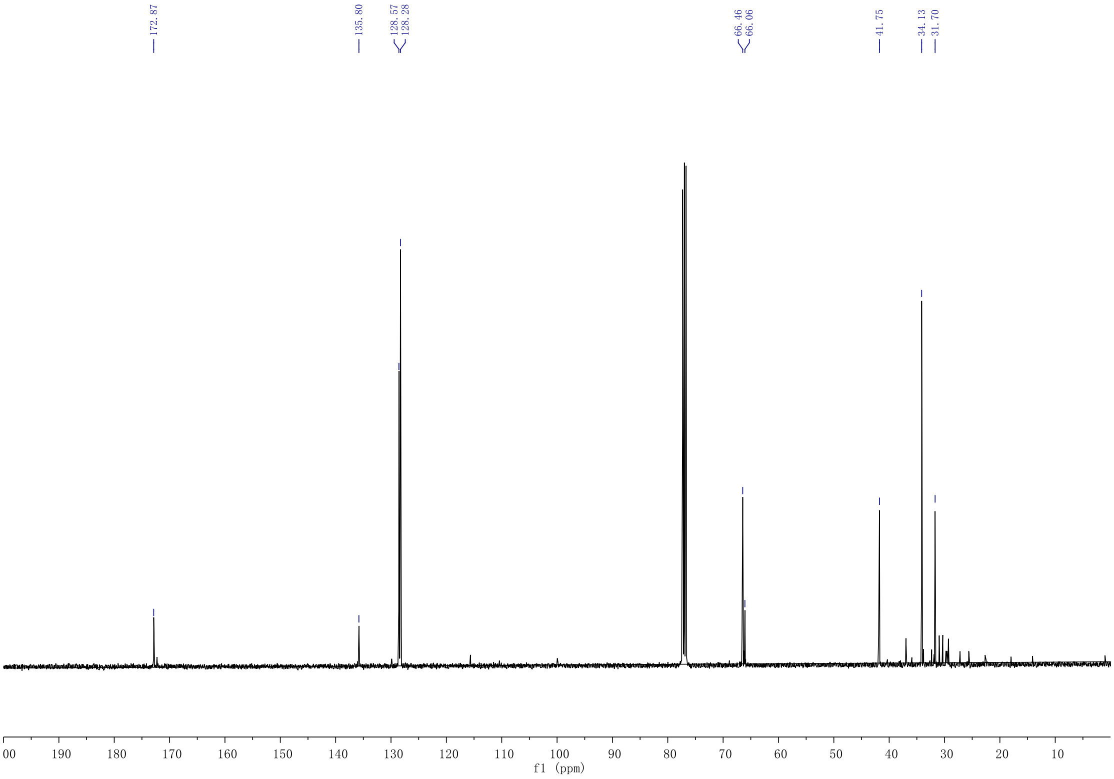
172.87
135.80
128.57
128.28
66.46
66.06
41.75
34.13
31.70
190
180
170
160
150
140
130
120
110
100
90
80
70
60
50
40
30
20
10
f1 (ppm)

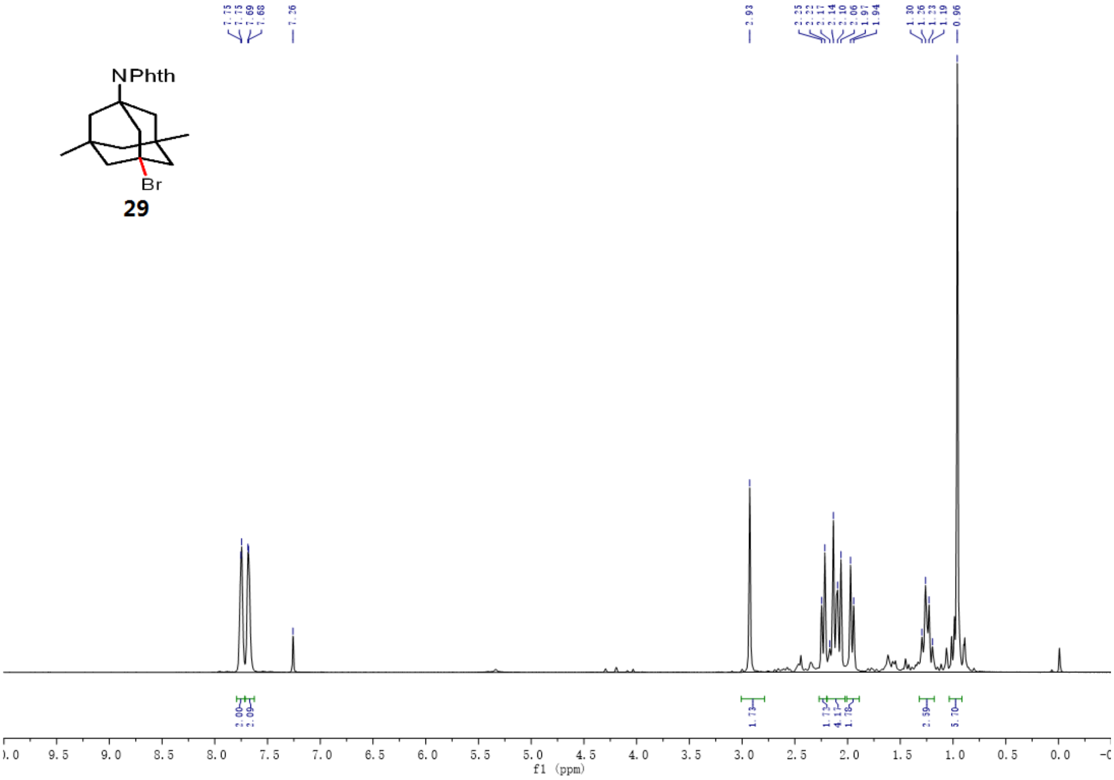
NPhth
Br

**29**

7.75
7.73
7.69
7.68
7.26
2.93
2.25
2.22
2.17
2.14
2.10
2.06
1.97
1.94
1.30
1.26
1.23
1.19
0.96
2.00
2.09
1.73
1.72
4.11
1.78
2.59
5.70
f1 (ppm)

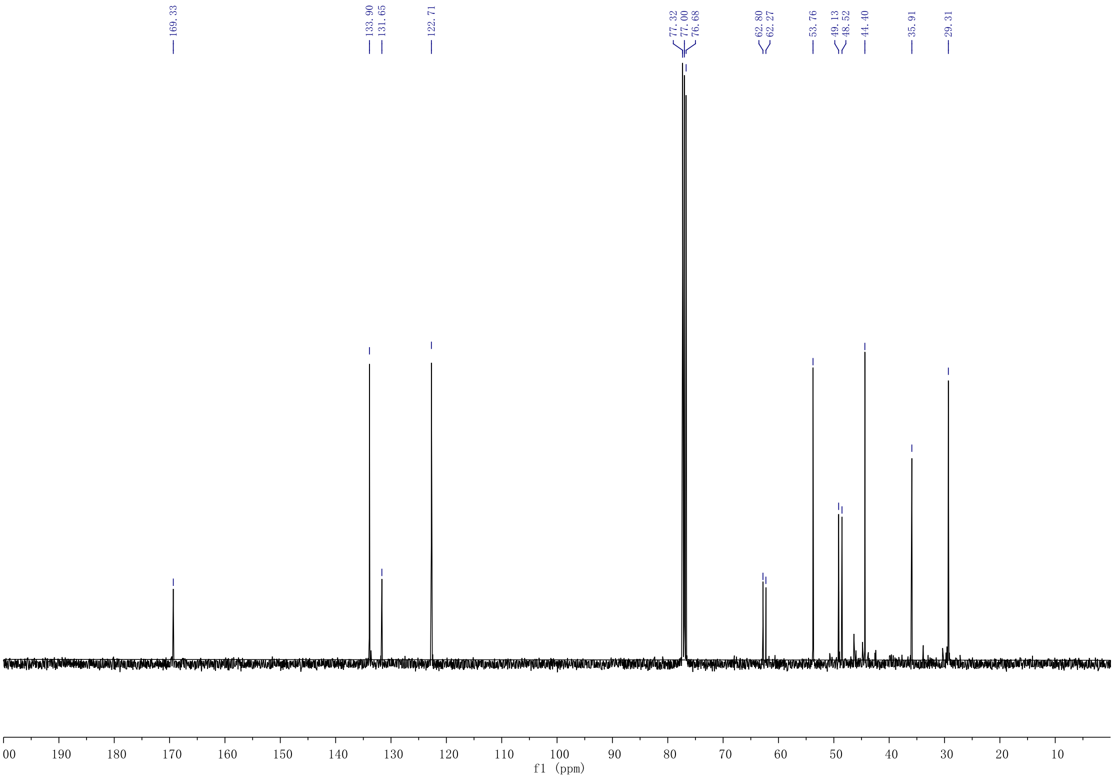
169.33
133.90
131.65
122.71
77.32
77.00
76.68
62.80
62.27
53.76
49.13
48.52
44.40
35.91
29.31
f1 (ppm)

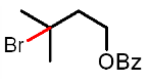
Br
OBz

**30**

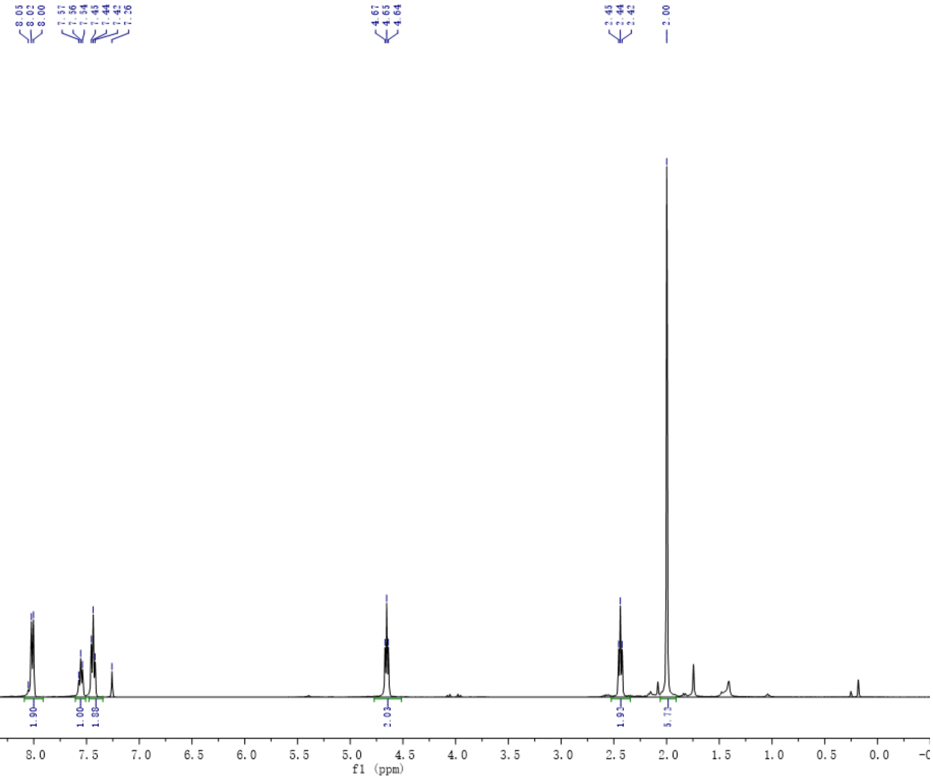
8.05
8.02
8.00
7.57
7.56
7.54
7.45
7.44
7.42
7.36
4.67
4.65
4.64
2.45
2.44
2.42
2.00
1.90
1.00
1.88
2.03
1.93
5.72
f1 (ppm)

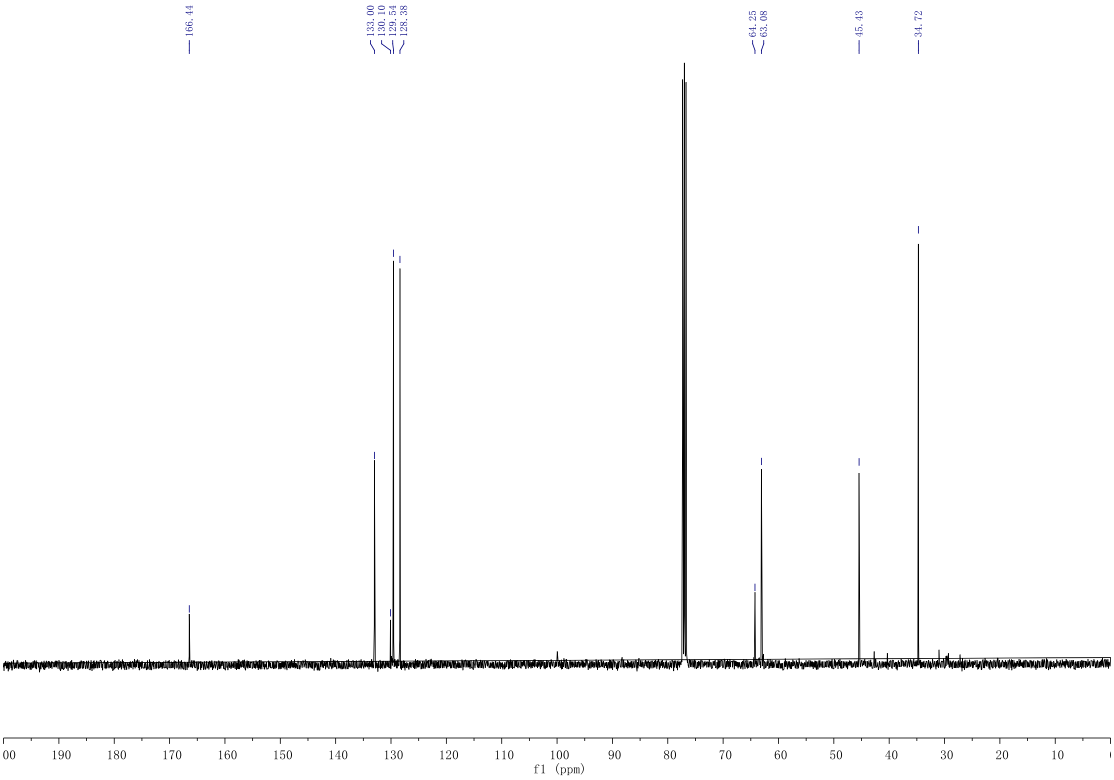
166.44
133.00
130.10
129.54
128.38
64.25
63.08
45.43
34.72
f1 (ppm)
